# Tomato receptor-like cytosolic kinase RIPK confers broad-spectrum disease resistance without yield penalties

**DOI:** 10.1093/hr/uhac207

**Published:** 2022-09-13

**Authors:** Ran Wang, Chenying Li, Qinghong Li, Yingfei Ai, Zeming Huang, Xun Sun, Jie Zhou, Yanhong Zhou, Yan Liang

**Affiliations:** Ministry of Agriculture Key Laboratory of Molecular Biology of Crop Pathogens and Insects, Department of Plant Protection, Zhejiang University, Hangzhou 310058, China; Ministry of Agriculture Key Laboratory of Molecular Biology of Crop Pathogens and Insects, Department of Plant Protection, Zhejiang University, Hangzhou 310058, China; Ministry of Agriculture Key Laboratory of Molecular Biology of Crop Pathogens and Insects, Department of Plant Protection, Zhejiang University, Hangzhou 310058, China; Ministry of Agriculture Key Laboratory of Molecular Biology of Crop Pathogens and Insects, Department of Plant Protection, Zhejiang University, Hangzhou 310058, China; Ministry of Agriculture Key Laboratory of Molecular Biology of Crop Pathogens and Insects, Department of Plant Protection, Zhejiang University, Hangzhou 310058, China; Ministry of Agriculture Key Laboratory of Molecular Biology of Crop Pathogens and Insects, Department of Plant Protection, Zhejiang University, Hangzhou 310058, China; Ministry of Agriculture Key Laboratory of Horticultural Plants Growth and Development, Department of Horticulture, Zhejiang University, Hangzhou 310058, China; Ministry of Agriculture Key Laboratory of Horticultural Plants Growth and Development, Department of Horticulture, Zhejiang University, Hangzhou 310058, China; Ministry of Agriculture Key Laboratory of Molecular Biology of Crop Pathogens and Insects, Department of Plant Protection, Zhejiang University, Hangzhou 310058, China

## Abstract

Production of reactive oxygen species (ROS) is an important immune response in plant multilayer defense mechanisms; however, direct modification of ROS homeostasis to breed plants with broad-spectrum resistance to disease has not yet been successful. In *Arabidopsis*, the receptor-like cytosolic kinase AtRIPK regulates broad-spectrum ROS signaling in multiple layers of the plant immune system. Upon treatment with immune elicitors, AtRIPK is activated and phosphorylates nicotinamide adenine dinucleotide phosphate (NADPH) oxidase, which leads to ROS production. In this study, we identified an AtRIPK ortholog in tomatoes and generated knockdown mutants using CRISPR/Cas9 technology. *Slripk* mutants displayed reduced ROS production in response to representative immune elicitors and were susceptible to pathogenic bacteria and fungi from different genera, including *Ralstonia solanacearum*, *Pectobacterium carotovorum*, *Botrytis cinerea*, and *Fusarium oxysporum*, which are leaf and root pathogens with hemibiotrophic and necrotrophic infection strategies. In contrast, transgenic tomato plants overexpressing *SlRIPK* are more resistant to these pathogens. Remarkably, the *slripk* mutants and *SlRIPK-*overexpressing transgenic plants did not exhibit significant growth retardation or yield loss. These results suggest that overexpression of *SlRIPK* confers broad-spectrum disease resistance without a yield penalty in tomato plants. Our findings suggest that modifying ROS homeostasis by altering the regulatory components of ROS production in plant immunity could contribute to engineering or breeding broad-spectrum disease-resistant crops without yield penalty.

## Introduction

Plant defenses are based on a multilayered system [1]. The first layer is based on pathogen/damage-associated molecular patterns (PAMPs/DAMPs), which are recognized by plasma membrane-localized pattern recognition receptors (PRRs) and stimulate PAMP/DAMP-triggered immunity (PTI/DTI). The second layer relies on cytosolic nucleotide-binding domain leucine-rich repeat (NLR) receptors, which detect polymorphic pathogen-secreted effectors, leading to effector-triggered immunity (ETI). PTI and ETI are mutually potentiated and induce similar immune responses, such as calcium influx, production of reactive oxygen species (ROS), activation of mitogen-activated protein (MAP) kinase, and expression of defense-related genes [2, 3]. However, compared with PTI, ETI is usually more robust and longer lasting. It also often causes hypersensitive cell death at the site of infection [4]. In addition to local responses, PTI and ETI can induce systemic immune responses in distal tissues, referred to as systemic acquired resistance (SAR) [5].

ROS are molecules that are chemically reactive, have microbicidal effects, and act as signals that trigger other immune responses [6, 7]. ROS signals in the immune system are primarily generated by NADPH oxidases (NOXs), which reduce oxygen to superoxide with NADPH as an electron donor [8, 9]. The superoxide is then converted into hydrogen peroxide (H_2_O_2_) by superoxide dismutase. Hydrogen peroxide is considered a ROS signal because it is a stable molecule that can be transported across the cell membrane [10, 11]. Plant NOXs belong to the respiratory burst oxidase homolog (RBOH) family, and in *Arabidopsis*, this family contains 10 members [12, 13]. RBOHD was identified as the primary enzyme responsible for the generation of ROS signals after pathogen infection in *Arabidopsis*, as *rbohD* mutants did not produce ROS signals after treatment with elicitors that induced PTI, ETI, and SAR [14, 15]. RBOHD regulation has been widely studied. It is activated by phosphorylation, binding of Ca^2+^ and phosphatic acid, persulfidation, and NADPH fueling, but is deactivated by nitrosylation and ubiquitination [15–18]. RBOHD is phosphorylated by various families of kinases, including receptor-like cytoplasmic kinases (RLCKs) (also known as AVRPPHB SUSCEPTIBLE1 (PBS1)-like kinases (PBLs)), MAP4 kinase SIK1, Ca^2+^-dependent protein kinases (CPKs), and cysteine-rich receptor-like protein kinase 2 (CRK2) [16, 19–21].

Among the RLCK-VII members studied to date, RPM1-INDUCED PROTEIN KINASE (RIPK) is the central kinase that regulates multilayered ROS production. *Ripk* mutants displayed decreased ROS production after treatment with several representative elicitors for PTI, ETI, and SAR. However, the activation of MAP kinase in response to these elicitors was not affected [18]. RIPK is activated after treatment with elicitors and then phosphorylates RBOHD, leading to its activation [18]. Simultaneously, RIPK phosphorylates NADP-malic enzyme 2 (NADP-ME2) to generate NADPH, which allows RBOHD to sustain ROS production [22]. In addition, RIPK is important for AvrRpm1-induced ETI responses because it phosphorylates the immune regulator RPM1-interacting protein 4 [23]. These studies suggest that RIPK may confer resistance by responding to various elicitors involved in different responses to biotic stressors in plants.

Plants encounter many types of pathogens in the natural environment; therefore, breeding crop varieties with broad-spectrum disease resistance is a major goal in agriculture [24]. Multiple strategies have been applied to achieve this goal, such as the modification of immune receptors and genome editing of susceptibility genes [24]. Increased immune responses enhance plant resistance against different types of pathogens; however, constitutive activation of immunity is costly and reduces crop yield [25, 26]. Therefore, it is crucial to trigger plant immune responses upon pathogen infection while keeping them inactive under normal growth conditions [27, 28]. ROS signaling plays a positive role in multilayer defense; thus, modulating it offers wide possibilities for broad-spectrum disease resistance. However, ROS are double-edged swords, and excessive ROS levels cause leaf senescence, which leads to a reduced yield [29]. Instead of directly modifying ROS-generating or ROS-scavenging enzymes to constitutively produce ROS, modifying genes that indirectly regulate ROS production could be an alternative method for improving plant defense after infection by pathogens.

Tomato (*Solanum lycopersicum*) is cultivated worldwide and is economically valuable. However, tomato plants are susceptible to many diseases during cultivation, and these diseases reduce the yield and quality of tomato fruits [30]. Bacterial wilt is one of the most severe tomato diseases and is caused by *Ralstonia solanacearum*, which has been classified as both a biotrophic and necrotrophic bacterium. *R. solanacearum* first attacks the roots of tomato plants through root wounds and then spreads through the xylem vessels, which leads to the rapid wilt and death of the infected plants [31, 32]. Soft rot disease is caused mainly by *Dickeya* and *Pectobacterium*, which are necrotrophic bacteria that usually attack both the leaves and fruits, and a characteristic symptom of infection is tissue maceration [33]. Leaf gray mold is caused by *Botrytis cinerea*, a necrotrophic fungus which infects not only tomatoes but also hundreds of other plant species. The typical symptom of gray mold is the presence of water-soaked spots along with a grayish fungus [34]. Tomato fusarium wilt is caused by the fungus *Fusarium oxysporum* f. sp. *lycopersici*, which invades roots and causes leaf yellowing and plant wilting [35]. Although many resistance genes for specific diseases have been identified thus far, it remains a challenge to breed a cultivar with resistance against both biotrophic and necrotrophic pathogens, and both root and leaf pathogens, without impairing yield.

In this study, we identified AtRIPK orthologs in tomatoes and generated *slripk* mutants using the CRISPR/Cas9 approach. The *slripk* mutants showed reduced ROS production after treatment with chitin, avirulent bacterium *Pseudomonas syringae* pv. *tomato* (*Pst*) DC3000 (*avrRpm1*), and pipecolic acid (pip), which are elicitors activating PTI, ETI, and SAR, respectively. In addition, we overexpressed *SlRIPK* in a wild-type (WT) tomato background (*SlRIPK-OE*). We found that *SlRIPK-OE* plants showed increased ROS production after treatment with the previously listed elicitors, and were more resistant to *R. solanacearum*, *Pectobacterium carotovorum* subsp*. carotovorum*, *B. cinerea*, and *F. oxysporum*. Notably, *SlRIPK-OE* plants showed the same growth and yield as control plants. Overall, these results suggested that SlRIPK-mediated ROS production confers broad-spectrum disease resistance to pathogens without any yield penalty.

## Results

### Identification of SlRIPK

**Figure 1:**
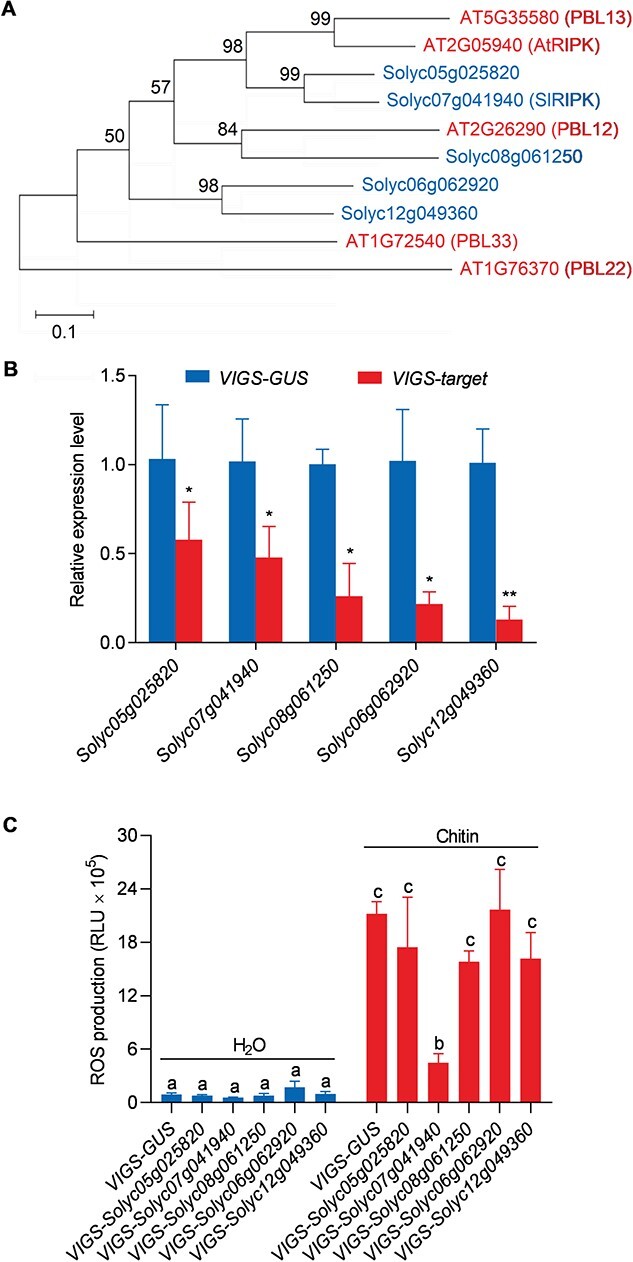
Chitin-induced ROS production in tomato leaves was reduced after silencing *Solyc07g041940* gene. **A** Phylogenetic tree of RIPK subgroups in *Arabidopsis thaliana* (AT) and *Solanum lycopersicum* (Solyc). A phylogenetic tree containing all PBL proteins is shown in Figure S1 (see online supplementary material). The phylogenetic tree was constructed using the maximum-likelihood method. The branches are labeled with their respective bootstrap values. Red represents *A. thaliana* and blue represents *S. lycopersicum*. **B** Silencing efficiency: *Solyc05g025820*, *Solyc06g062920*, *Solyc07g041940*, *Solyc08g061250*, and *Solyc12g049360* were silenced using virus-induced gene silencing (VIGS). The relative transcript levels of these genes in the leaves were determined using qRT-PCR analysis four weeks after infiltration with *Agrobacterium tumefaciens* carrying its cognate VIGS constructs, and VIGS-GUS was used as a negative control. Data are shown as the mean ± SD (*n* = 3). Asterisks indicate significant differences compared with the VIGS-GUS control (^*^P ≤ 0.05, ^**^P ≤ 0.01, *t*-test). **C** Chitin induced ROS production in tomato leaves after silencing the indicated genes. ROS levels were measured using a luminol-based chemiluminescent assay after treatment with chitin (20 μg/mL). Total ROS production within 30 min is shown. Data are shown as the mean ± SD (*n* = 8). Different letters above the bars indicate significant differences between the different genotypes (*P* ≤ 0.05, one-way ANOVA).

AtRIPK belongs to the RLCK-VII family and to the same group as PBL12 and PBL13. To identify the orthologs of *AtRIPK* in tomatoes, we downloaded the full-length amino acid sequences of 46 PBLs from *Arabidopsis* and 56 PBLs from *S. lycopersicum*, and then generated a phylogenetic tree. In this tree, two genes from *S. lycopersicum*, *Solyc05g025820* and *Solyc07g041940*, grouped into a subclade with *AtRIPK* and *PBL13* (Fig. 1A and Fig. S1, see online supplementary material). *Solyc08g061250* clustered with *PBL12* into one subgroup, and *Solyc06g062920* and *Solyc12g049360* arose before the divergence of the *RIPK* group and other *PBL* groups. Therefore, we selected these genes as candidate genes for *RIPK* in tomato plants. Next, we investigated whether these genes were involved in ROS production using virus-induced gene silencing (VIGS) approach. The tomato Zheza809 cultivar was used to perform VIGS experiments because of its high silencing efficiency [36]. The candidate genes were individually silenced by infiltrating cognate *VIGS* constructs into tomato leaves. *VIGS*-*GUS* (*β-GLUCURONIDASE*) was used as a negative control. After confirming the silencing effectiveness using quantitative reverse transcription PCR (qRT-PCR) (Fig. 1B), the plants were treated with chitin and the production of ROS in their leaves was measured using a luminol-based chemiluminescence assay (Fig. 1C). We found that leaves infiltrated with the *VIGS*-*Solyc07g041940* construct showed lower levels of ROS after chitin treatment when compared with the *VIGS*-*GUS* control, whereas plants infiltrated with other constructs did not exhibit significantly reduced ROS production (Fig. 1C). These results suggested that *Solyc07g041940* could be the ortholog of *AtRIPK* in tomato; therefore, we focused on *Solyc07g041940*, which is referred to as *SlRIPK* hereinafter.

**Figure 2:**
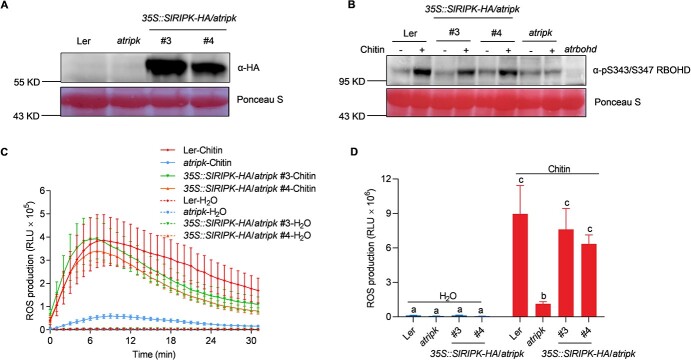
Ectopic expression of *SlRIPK* complements the ROS production in Arabidopsis *ripk* mutants. **A** Protein abundance of SlRIPK-HA in two independent *35S::SlRIPK-HA/atripk* transgenic *Arabidopsis* lines. Total proteins were extracted from 10-d-old seedlings and the abundance of SlRIPK-HA was detected by immunoblot analysis with an α-HA antibody, and Ponceau S staining of the membrane was used as a loading control. **B** Chitin induced phosphorylation of RBOHD in *35S::SlRIPK-HA*/*atripk* transgenic plants. The total protein content was extracted from 10-d-old seedlings 15 min after treatment with or without chitin (100 μg/mL). The phosphorylation of RBOHD was determined using an immunoblot analysis and an α-pS343/S347 RBOHD antibody. Ponceau S staining of the membrane was used as a loading control. **C, D** Chitin induced ROS production in *35S::SlRIPK-HA*/*atripk* transgenic leaves. ROS signals were monitored using a chemiluminescent assay after treatment with or without chitin (20 μg/ml). Line graphs were plotted with values recorded every minute (**C**), and the total ROS production within 30 min is shown in (**D**). Data are shown as the mean ± SD (*n* = 8). Different letters above the bars indicate significant differences between the different genotypes (*P* ≤ 0.05, one-way ANOVA). The experiment was repeated twice, and similar results were obtained.

To confirm that SlRIPK has the same function as AtRIPK in ROS production, we expressed *SlRIPK-HA* driven by the cauliflower mosaic virus 35S promoter in an *atripk* mutant background (*35S::SlRIPK*-*HA*/*atripk*). SlRIPK-HA protein abundance was detected using an immunoblot assay and an α-HA antibody in the two independent transgenic lines (Fig. 2A). The level of chitin-induced RBOHD phosphorylation and ROS production in both lines was higher than that in *atripk* mutants, suggesting that ectopic expression of SlRIPK-HA partially complemented the reduced ROS production in the *atripk* mutants (Fig. 2B–D). Overall, these results suggest that Solyc07g041940 (SlRIPK) is an ortholog of AtRIPK.

### *Slripk* mutants show reduced ROS signaling in PTI, ETI, and SAR

**Figure 3:**
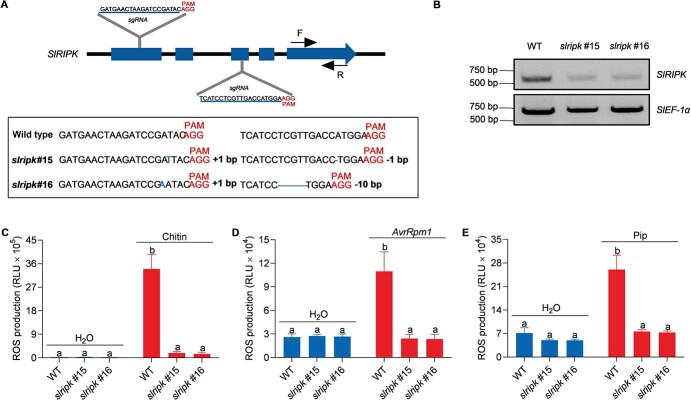
*Slripk* mutants show decreased ROS production. **A** Schematic representation of *SlRIPK*. Blue boxes and black lines represent the exons and introns, respectively. The gRNA target sequences for CRISPR/Cas9 are shown in black capital letters, and the PAM sites are indicated in red; mutations in *slripk* plants are labeled in blue. **B** Transcript levels of *SlRIPK* in *slripk* mutants. Primers were designed at the 3′ end of *SlRIPK*, as indicated with arrows in (**A**), and transcript levels of *SlRIPK* were detected using qRT-PCR. RNA was extracted from 4-week-old leaves and *SlEF-1α* (*Solyc06g005060*) was used as an internal control. **C, D, E***slripk* mutants showed decreased ROS production after treatment with 20 μg/mL chitin (**C**) and *Pseudomonas syringae* pv. *tomato* (*Pst*) DC3000 (*avrRpm1*) at an OD_600_ of 0.3 (**D**), and 1 mM of Pip (**E**). Data are shown as the mean ± SE (*n* = 8). Different letters above the bars indicate significant differences between the different genotypes (*P* ≤ 0.05, one-way ANOVA). The experiment was repeated three times, with similar results.

To further explore the biological function of SlRIPK, we generated tomato *slripk* mutants in a Micro-Tom background, which is used as a model cultivar for tomato genetic studies because of its small plant size and short life cycle [37]. The full-length coding sequences of *SlRIPK* from the Micro-Tom and Zheza809 cultivars were identical to those from the reference genome of Heinz (Fig. S2, see online supplementary material). The *slripk* mutant lines were generated using CRISPR/Cas9 technology. To this end, gRNA target sequences were designed in the first and third exons for simultaneous editing (Fig. S3A and B, see online supplementary material). Two independent *slripk* mutant lines, *slripk* #15 and *slripk* #16, were selected for further analysis (Fig. 3A). *slripk* #15 contained a one-base insertion in the first target sequence and a one-base deletion in the second target sequence. *Slripk* #16 contained a one-base insertion in the first target sequence and a ten-base deletion in the second target sequence (Fig. 3A). RT-PCR analysis confirmed the reduced transcript levels of *SlRIPK* in the *slripk* mutants compared with the WT (Fig. 3B).

We examined ROS production in *slripk* mutants after treatment with a range of representative immune elicitors, including chitin for PTI, *Pst* DC3000 (*avrRpm1*) for ETI, and pip for SAR. Our results indicated that the production of ROS triggered by these elicitors was significantly lower in the *slripk* mutants than in the WT plants (Fig. 3C–E). Taken together, these results suggest that SlRIPK plays roles in the production of ROS that are similar to those of AtRIPK during the induction of PTI, ETI, and SAR.

### *Slripk* mutants are susceptible to pathogens

**Figure 4:**
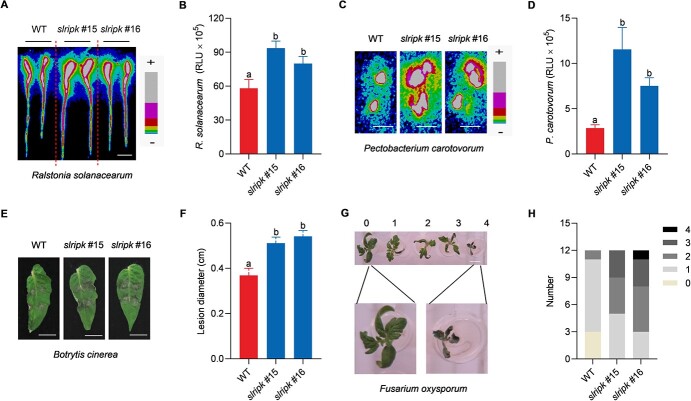
*Slripk* mutants are susceptible to pathogenic bacteria and fungi. **A** Representative image of tomato seedlings inoculated with *Ralstonia solanacearum*-LuxCDABE. The roots of 10-d-old WT and *slripk* mutants were soaked in a bacterial suspension (OD_600_ = 1.45) for 2 min, and luminescent signals were detected using a photon camera 4 d post-inoculation. +: Strong; −: Weak. Bar, 1 cm. **B** Growth of *R. solanacearum*-LuxCDABE. The experimental conditions were the same as those described in (**A**), and bacterial growth was quantified using relative light units (RLU) released during the expression of LuxCDABE. Data are shown as the mean ± SD (*n* = 10). Different letters above the bars indicate significant differences between the different genotypes (*P* ≤ 0.05, one-way ANOVA). All experiments were repeated twice, and similar results were obtained. **C** Representative images of tomato leaves inoculated with *Pectobacterium carotovorum-*LuxCDABE. Six-week-old leaves of WT and *slripk* mutants were spot-inoculated with bacteria (OD_600_ = 0.6), and luminescence signals were detected using a photon camera 12 h post-inoculation. +: Strong; −: Weak. Bars, 1 cm. **D** Growth of *P. carotovorum*-LuxCDABE. The experimental conditions were the same as those described in (**C**), and bacterial growth was quantified based on RLU released during the expression of LuxCDABE. Data are shown as the mean ± SD (*n* = 14). Different letters above the bars indicate significant differences between the different genotypes (*P* ≤ 0.05, one-way ANOVA). All experiments were repeated twice, and similar results were obtained. **E** Representative images of the leaves inoculated with *Botrytis cinerea*. Six-week-old leaves of WT and *slripk* mutants were spot-inoculated with 2.5 μL of spore suspension (1 × 10^5^ spores/mL). Images were taken 3 d post-inoculation. Bars, 1 cm. **F** Lesion diameter of leaves inoculated with *B. cinerea*. The experimental conditions were identical to those described in (**E**). Data are shown as the mean ± SD (n = 10). Different letters above the bars indicate significant differences between the different genotypes (*P* ≤ 0.05, one-way ANOVA). All experiments were repeated twice, and similar results were obtained. **G** Representative images of seedlings inoculated with *Fusarium oxysporum*. Ten-day-old tomato seedlings were inoculated with 1 × 10^7^ spores/mL *F. oxysporum* using a hydroponic method. Disease severity was rated as follows: 0, no symptoms; 1, cotyledons began to show signs of wilting; 2, cotyledons completely withered; 3, true leaves began to wither and wilt; and 4, the whole plant wilted and died. Bars, 1 cm. **H** Disease severity of seedlings inoculated with *F. oxysporum*. The experimental conditions were identical to those described in (**G**). The disease severity of seedlings from the WT and *slripk* mutants was recorded 1 d post-inoculation. All experiments were repeated twice and yielded similar results.

We determined the susceptibility of *slripk* mutants to *R. solanacearum* using a bioluminescent strain expressing LuxCDABE, which allows bacterial growth to be quantified by measuring light intensity [38]. The tomato seedlings were inoculated with *R. solanacearum*-LuxCDABE, and the light intensity was measured 4 d after inoculation. Compared with the WT, *slripk* mutants showed significantly higher signals after inoculation with *R. solanacearum*-LuxCDABE (Fig. 4A and B). Similarly, when we exposed the tomato seedlings to *P. carotovorum*-LuxCDABE, we found that the light signals in *slripk* mutants were significantly higher than those in the WT plants, suggesting that *slripk* mutants are very susceptible to *P. carotovorum*-LuxCDABE infection (Fig. 4C and D). This is consistent with the function of *Arabidopsis RIPK* in terms of its positive role in resistance to *P. carotovorum* [18]. Taken together, our results suggest that *SlRIPK* confers resistance to two tomato bacterial pathogens, *R. solanacearum* and *P. carotovorum*.

We then examined whether SlRIPK plays a positive role in resistance to two tomato fungal pathogens, *B. cinerea* and *F. oxysporum*. Detached leaves of WT and *slripk* mutants were spot-inoculated with *B. cinerea*, and disease severity was determined and compared by measuring lesion diameter. Compared to the WT plants, the lesion diameters in *slripk* mutants were 25% larger (Fig. 4E and F), suggesting that *slripk* mutants were more susceptible to *B. cinerea*. In addition, seedlings from WT plants and the *slripk* mutants were inoculated with *F. oxysporum* using a hydroponic method, and disease severity was scored 1 d post-inoculation (Fig. 4G). The number of plants with severe symptoms was much higher in the case of *slripk* mutants than in the case of WT plants; in other words, *slripk* mutants wilted more severely than the WT plants (Fig. 4H). Taken together, these results suggest that *slripk* mutants are not only more susceptible to shoot and root bacterial diseases but also to shoot and root fungal diseases.

### Overexpression of SlRIPK shows increased resistance to pathogens

**Figure 5:**
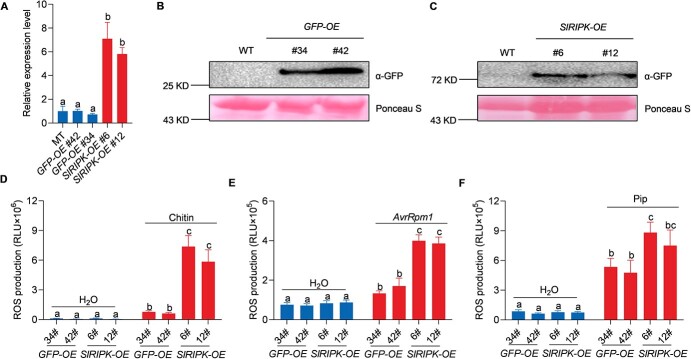
*SlRIPK* overexpression transgenic plants show increased ROS production after elicitor treatment. **A** Relative expression levels of *SlRIPK* in Micro-Tom (WT), *35S::GFP* (*GFP-OE*), and *35S::SlRIPK-GFP* (*SlRIPK-OE*) transgenic plants. RNA was extracted from 4-week-old leaves, and gene expression was quantified using qRT-PCR. Data are presented as the mean ± SE (*n* = 3). Different letters above the bars indicate significant differences between the different genotypes (*P* ≤ 0.05, one-way ANOVA). **B, C** Protein levels in *GFP-OE* and *SlRIPK-OE* transgenic lines. Total protein was extracted from 4-week-old leaves. Free GFP and SlRIPK-GFP were detected using an immunoblot analysis and an α-GFP antibody. Ponceau S staining of the membrane served as the loading control. **D, E, F***SlRIPK-OE* transgenic plants showed increased ROS production after treatment with 20 μg/mL chitin (**D**), *Pseudomonas syringae* pv. *tomato* (*Pst*) DC3000 (*avrRpm1*) at an OD_600_ of 0.3 (**E**), and 1 mM of Pip (**F**). Data are shown as the mean ± SE (*n* = 8). Different letters above the bars indicate significant differences between different genotypes (*P* ≤ 0.05, one-way ANOVA). The experiment was repeated three times, with similar results.

The above-mentioned results suggest that SlRIPK might regulate ROS signaling in multiple layers of plant immunity, thus conferring broad-spectrum disease resistance. Therefore, we generated transgenic plants that overexpressed *SlRIPK*. To this end, we fused *SlRIPK* coding sequences with the *GREEN FLUORESCENCE PROTEIN* (*GFP*) gene and expressed them under the control of 35S promoter in a Micro-Tom background, hereinafter referred to as *SlRIPK-OE* (Fig. S3C and D). Plants overexpressing *GFP* alone (*GFP-OE*) were used as a negative control. The transcript levels of *SlRIPK* in the two independent *SlRIPK-OE* lines were significantly higher than those in the *GFP-OE* plants (Fig. 5A). The SlRIPK-GFP or GFP proteins were detected using an immunoblot assay with an α-GFP antibody, and the size of the SlRIPK-GFP band was the same as we predicted (75 kDa) (Fig. 5B and C). SlRIPK-GFP proteins were mainly expressed in the peripheral regions of the leaf cells (Fig. S4, see online supplementary material). These results suggested that SlRIPK was correctly fused to GFP and overexpressed in *SlRIPK-OE* transgenic plants. Moreover, *SlRIPK-OE* transgenic plants displayed increased ROS production after treatment with chitin, *Pst* DC3000 (*avrRpm1*), and pip (Fig. 5D–F). Taken together, these results suggest that SlRIPK overexpression enhances ROS signaling in PTI, ETI, and SAR.

In *Arabidopsis*, ROS signaling in plant immunity is primarily mediated by AtRBOHD. Phylogenetic analysis indicated that two genes from *S. lycopersicum*, *SlRBOHB* (*Solyc03g117980*) and *SlRBOHD* (*Solyc06g068680*), clustered with *AtRBOHD* into a single subclade (Fig. S5A). Silencing *SlRBOHB* in tomato has been shown to reduce flg22-induced ROS production [39]. Because AtRIPK associated with AtRBOHD in the absence of elicitors [18], we detected the interaction between SlRIPK and SlRBOHB without elicitor treatment. Split-luciferase and bimolecular fluorescence complementation assays were performed in *Nicotiana benthamiana* (Fig. S5, see online supplementary material). Strong interactions were observed when SlRIPK was co-expressed with SlRBOHB, but not with the CONSTITUTIVE EXPRESSOR OF PATHOGENESIS-RELATED GENES5 (CPR5). We used CPR5 as a negative control in our previous studies to determine how RIPK and NADP-ME2 interacted [22]. We validated the expression of fusion proteins using immunoblot analysis with α-luciferase and α-GFP antibodies (Fig. S5, see online supplementary material). Taken together, these results suggest that it is possible that a conserved module of SlRIPK-SlRBOHB regulates ROS signaling in tomatoes.

**Figure 6:**
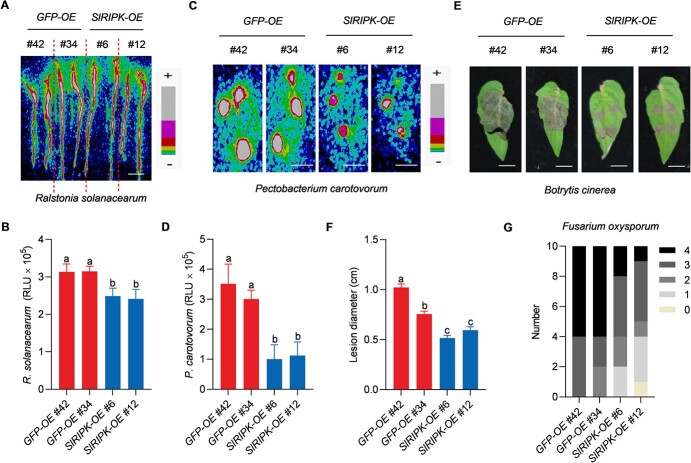
Overexpression of SlRIPK confers resistance to tomato against a range of pathogens. **A** Representative image of tomato seedlings inoculated with *Ralstonia solanacearum*-LuxCDABE. The roots of 10-d-old *SlRIPK-OE* (3*5S::SlRIPK-GFP*) and *GFP-OE* (*35S::GFP*) transgenic plants were soaked in a bacterial suspension (OD_600_ = 1.45) for 2 min, and luminescent signals were detected using a photon camera 3 d post-inoculation. +: Strong; −: Weak. Bar, 1 cm. **B** Growth of *R. solanacearum*-LuxCDABE. The experimental conditions were the same as those described in (**A**), and bacterial growth was quantified based on the RLU released during the expression of LuxCDABE. Data are shown as the mean ± SD (*n* = 10). Different letters above the bars indicate significant differences between the different genotypes (*P* ≤ 0.05, one-way ANOVA). All experiments were repeated twice, and similar results were obtained. **C** Representative images of tomato leaves inoculated with *Pectobacterium carotovorum-*LuxCDABE. Six-week-old leaves from *SlRIPK-OE* and *GFP-OE* plants were spot-inoculated with bacteria (OD_600_ = 0.6), and luminescence signals were detected using a photon camera 12 h post-inoculation. +: Strong; −: Weak. Bar, 1 cm. **D** Growth of *P. carotovorum*-LuxCDABE. The experimental conditions were the same as those described in (**C**), and bacterial growth was quantified based on the RLU released during the expression of LuxCDABE. Data are shown as the mean ± SD (*n* = 14). Different letters above the bars indicate significant differences between the different genotypes (*P* ≤ 0.05, one-way ANOVA). All experiments were repeated twice, and similar results were obtained. **E** Representative images of the leaves inoculated with *Botrytis cinerea*. Six-week-old leaves from *SlRIPK-OE* and control plants were spot-inoculated with 5 μL spore suspension (1 × 10^5^ spores/mL). Images were taken 3 d post-inoculation. Bars, 1 cm. **F** Lesion diameter of leaves inoculated with *B. cinerea*. The experimental conditions were the same as those described in (**E**). Data are shown as the mean ± SD (*n* = 10). Different letters above the bars indicate significant differences between the different genotypes (*P* ≤ 0.05, one-way ANOVA). All experiments were repeated twice, and similar results were obtained. **G** Disease severity of seedlings inoculated with *F. oxysporum*. Ten-day-old tomato seedlings were inoculated with 1 × 10^8^ spores/mL *F. oxysporum* using a hydroponic method. Disease severity was rated as shown in Fig. 4G. All experiments were repeated twice, and similar results were obtained.

We then inoculated *SlRIPK-OE* and *GFP-OE* plants with *R. solanacearum*-LuxCDABE and *P. carotovorum*-LuxCDABE, respectively. We found that plants from the *SlRIPK-OE* transgenic lines showed reduced light intensity when compared to *GFP-OE* plants (Fig. 6A–D), suggesting that overexpression of *SlRIPK* enhanced plant resistance to *R. solanacearum* and *P. carotovorum*. In addition, overexpression of *SlRIPK* consistently enhanced resistance to *B. cinerea* and *F. oxysporum* (Fig. 6E–G). Furthermore, transcript levels of *SlRIPK* were detected by qRT-PCR in transgenic plants after pathogen infection. Our results showed that the *SlRIPK* expression was significantly upregulated in the *SlRIPK-OE* plants after infection with all the tested pathogens, including *R. solanacearum*, *P. carotovorum*, *B. cinerea*, and *F. oxysporum* (Fig. S6, see online supplementary material). In contrast, *SlRIPK* expression in *GFP-OE* plants was only upregulated after *B. cinerea* infection, but the increase of *SlRIPK* expression was smaller in *GFP-OE* plants than in *SlRIPK-OE* plants (Fig. S6C, see online supplementary material). Overall, overexpression of *SlRIPK* confers resistance to two bacterial and two fungal pathogens, including shoot and root pathogens.

### Mutation and overexpression of *SlRIPK* does not alter tomato yield

To further investigate whether SlRIPK overexpression has an impact on tomato growth and yield, we cultivated WT and *slripk* mutants under the same conditions and observed their growth and development throughout the seedling, vegetative, flowering, and fruit ripening stages. Tomato seedlings were vertically grown on agar media, and we found that the *slripk* mutants were healthy and had no significant growth retardation*,* although they seemed to have shorter lateral roots than the WT plants (Fig. S7A, see online supplementary material). After 10 d, the tomato seedlings were transferred to pots so they could continue to grow. For the rest of their lives, we did not find any significant differences between the *slripk* mutants and the WT, including leaf size, flowering, and fruit ripening time (Fig. S7B–D). In addition, the mutation of *SlRIPK* did not affect yield components, such as fruit number per plant or fruit size, as indicated by measurements of length, width, and fresh weight per fruit (Fig. S7E–I). These results suggest that the *SlRIPK* mutation does not impair tomato yield.

**Figure 7:**
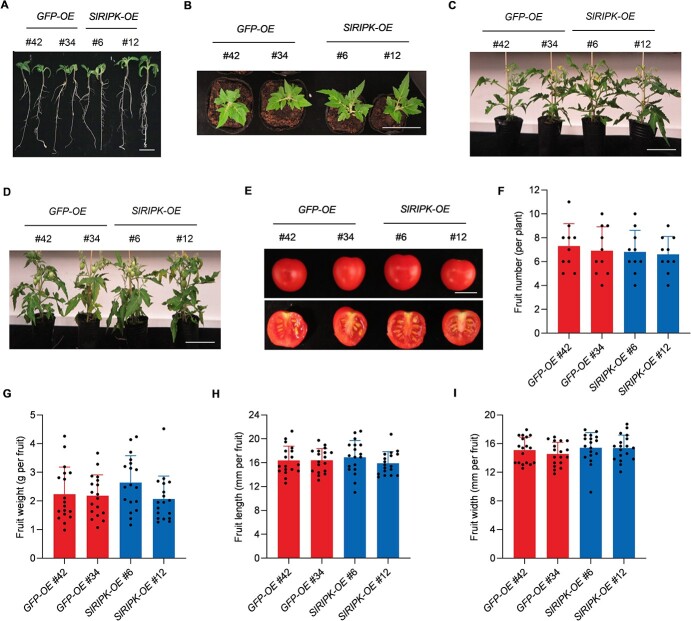
Overexpression of SlRIPK does not reduce the tomato yield. **A** Representative image of 10-d-old seedlings. *SlRIPK-OE* (3*5S::SlRIPK-GFP*) and *GFP-OE* (*35S::GFP*) transgenic plants were grown on agar medium. Bar, 1 cm. **B** Representative image of one-month-old tomato plants. Ten-day-old *GFP-OE* and *SlRIPK-OE* transgenic seedlings were transferred from agar media to pots and grown for another 20 d. Bar, 10 cm. **C** Representative images of two-month-old tomato plants. Bar, 10 cm. **D** Representative image of three-month-old tomato plants. Bar, 10 cm. **E** Representative image of four-month-old red-ripened fruits. Bar, 1 cm. **F** Number of fruits per plant. The number of red, ripened fruits from four-month-old plants was counted. Data are presented as the mean ± SD (n = 10). **G** Fruit weight. Data are presented as the mean ± SD (*n* = 18). **H** Fruit length. Data are presented as the mean ± SD (*n* = 18). **I** Fruit width. Data are presented as the mean ± SD (*n* = 18).

We then examined whether the overexpression of *SlRIPK* impairs the growth and yield of tomato plants. When *SlRIPK-OE* transgenic seedlings were grown on agar media, they were markedly stronger than control seedlings (Fig. 7A). During their early vegetative stages, or less than one month after the plants were transferred to the pots, the *SlRIPK-OE* transgenic plants were still slightly larger than the control plants (Fig. 7B). However, the flowering and fruit ripening times of *SlRIPK-OE* plants were similar to those of *GFP-OE* plants (Fig. 7C and D). Moreover, the yield of SlRIPK-OE plants was not significantly different from that of *GFP-OE* plants (Fig. 7E–I). These results suggest that *SlRIPK* overexpression confers broad-spectrum resistance to a variety of diseases without causing yield loss.

## Discussion

Microbial diseases are major factors limiting crop production. Improving host broad-spectrum disease resistance against a range of pathogen species is one of the most economical and environmentally friendly approaches to solve this problem [24, 40, 41]. However, the identification of genes that can confer broad-spectrum disease resistance without yield penalties has been difficult [28]. In this study, we identified tomato SlRIPK, a central protein for the production of ROS, and found that *SlRIPK* overexpression confers resistance to a variety of pathogens without any yield penalty. Overall, our results suggest a strategy for improving broad-spectrum disease resistance by modifying the regulatory mechanisms for the production of ROS.

One of the main strategies for improving broad-spectrum disease resistance is enhancing the plant immune system. Because PRRs recognize relatively conserved molecules from microbes, PRR-mediated resistance mechanisms have strong potential for the generation of broad-spectrum resistant cultivars in plants [24]. The intragenic overexpression of *Arabidopsis* EF-TU RECEPTOR (EFR), a PRR that recognizes the bacterial elongation factor (EF) Tu, in *N. benthamiana*, *Medicago truncatula,* tomato, potato, and rice has been known to activate defenses against various bacterial pathogens [42, 43]. Moreover, the enhancement of SAR provides a good strategy for breeding strains with broad-spectrum disease resistance. For example, overexpression of NONEXPRESSER of PR GENES1 (NPR1), a well-known positive regulator of SAR, enhances resistance to many pathogens [44]. NLR*-*mediated resistance is usually considered race-specific; however, eight *NLR* genes have been cloned to show broad-spectrum resistance in rice [45].

Although the enhancement of immunity conferred enhanced disease resistance, in many cases it also resulted in fitness costs with reduced growth and lower yield. Growth and immunity are often negatively regulated. In other words, the constitutive expression of immune responsive genes can impede plant growth and environmental fitness [25, 27, 28]. For example, overexpression of a rice NPR1 homolog confers disease resistance; however, rice plants that overexpress this protein show growth retardation and spontaneous cell death [46]. In this study, we found that tomato RIPK conferred disease resistance without a growth penalty. A possible explanation for this phenomenon is that the role of RIPK in ROS is not activated under normal growth conditions but is only activated upon pathogen attack. Furthermore, consistent with the role of *Arabidopsis* RIPK in root development [47], we found that tomato SlRIPK also positively regulated root and seedling growth but did not seem to function in the reproductive organs. Overall, this study provides a possible mechanism to ameliorate the trade-off between growth and defense by expressing inactive PAMP signaling components, which are activated only upon infection.

RIPK is an important member of the RLCK-VII family, and many members of this family have been shown to play a role in plant immunity. Botrytis-induced kinase 1 (BIK1), the most well-studied member of this family, was initially identified because of its role in the defense against *Botrytis* [48]. BIK1 is also activated by PRRs to mediate PTI signal transduction [49–51]. Notably, overexpression of BROAD-SPECTRUM RESISTANCE 1 (BSR1; OsRLCK278), a rice RLCK-VII member, confers resistance to several diseases, including rice blast caused by *Magnaporthe oryzae*, brown spot caused by the necrotrophic fungus *Cochliobolus miyabeanus*, bacterial leaf blight caused by *Xanthomonas oryzae* pv. *oryzae*, and seedling rot caused by the necrotrophic bacterium *Burkholderia glumae* [52–54]. Although it remains unknown whether BSR1 regulates broad-spectrum ROS production, knocking out BSR1 significantly suppresses ROS production triggered by chitin, lipopolysaccharides, and peptidoglycans [54]. In this study, we found that overexpression of SlRIPK in tomato confers resistance to four pathogens, including two bacterial and two fungal species. Phylogenetic analysis indicated that SlRIPK and BSR did not cluster into a single subgroup; therefore, whether tomato BSR or rice OsRIPK could confer broad-spectrum disease resistance remains to be studied.

ROS signals are important for growth and defense. However, high ROS levels cause oxidative stress, which can accelerate cell death and organ senescence. Therefore, ROS homeostasis must be tightly controlled by enzymatic and non-enzymatic mechanisms, including ascorbate peroxidase, catalase, glutathione peroxidase, and the ascorbate-glutathione system. Because continuous accumulation of ROS is toxic to plants, directly increasing ROS levels by decreasing the activity of the ROS-scavenging system is not a good strategy. However, the indirect inhibition of the ROS-scavenging system by pathogen-induced regulatory components has recently been applied to several broad-spectrum genes. The transcription factor BSR-D1 targets peroxidase in rice, leading to increased levels of ROS after pathogen infection and enhanced resistance to different races of blast [55]. ROD1, a C2 domain Ca^2+^ sensor, inhibits catalase B to increase ROS levels, which confers broad-spectrum disease resistance to multiple bacterial and fungal pathogens [56]. Rice methyl esterase-like (OsMESL) regulates thioredoxin OsTrxm to promote ROS production, and *osmesl* mutants show significant resistance to *X. oryzae* pv. *oryzae*, *Rhizoctonia solani* and *M. oryzae* [57]. Overall, these studies suggest that it is possible to breed broad-spectrum disease-resistant crops by modifying their levels of ROS. In this study, we developed another method to enhance ROS signaling. Upon pathogenic infection, RIPK is phosphorylated and activates RBOHD to produce ROS signals. Therefore, overexpression of *SlRIPK* allows for a transient increase in ROS signaling in response to pathogen infection, while relatively low levels of ROS are maintained under normal plant growth conditions. This provides a strategy for modifying the levels of ROS by enhancing the regulatory components of ROS production. In addition, ROS signals play roles in conferring resistance to different classes of pathogens, including bacteria, oomycetes, and fungi. It is conceivable that overexpression of SlRIPK may also confer resistance to other oomycetes, viruses, or even insects.

In conclusion, our study highlights a novel strategy for breeding durable, disease-resistant crop species. In addition, overexpression of RIPK can be achieved without significant yield penalties and may even provide growth benefits.

## Materials and methods

### Plant materials and growth conditions

The *Arabidopsis ripk-1* mutant was used in this study as previously described [18]. *S. lycopersicum* cv. Zheza809 (Zhejiang Academy of Agricultural Sciences, China) was used for VIGS experiments [36], and *S. lycopersicum* cv. Micro-Tom (Biogle GeneTech, Hangzhou, China) was used to generate stable transgenic tomato plants [37], including *slripk* mutants and *SlRIPK-*overexpressing plants. *Arabidopsis* and tomato plants were grown in separate plant growth rooms at 22°C and 25°C, respectively, with a 16 h photoperiod.

### Plasmid construction and generation of transgenic plants

The sequences of the primers used for plasmid construction are listed in Table S1 (see online supplementary material) and all other information about the PCR conditions used for the amplification of DNA fragments is listed in Table S2 (see online supplementary material). This information includes the purpose of the PCR, the template and primers used during the PCR, the size of the expected PCR product, and the sequences of the amplified DNA fragments. Most plasmids used in this study were generated using a gateway system. The amplified DNA fragments were cloned into the pDONR-Zeo plasmid using BP cloning (Thermo Fisher Scientific, Waltham, MA, USA) and confirmed by sequencing. The inserts were cloned into the destination plasmids using LR cloning (Thermo Fisher Scientific). Plasmid pTRV2 was used for the VIGS experiments, and gene silencing assays were performed as previously described [36]. pGWB14 was used for the ectopic expression of *SlRIPK* in an *atripk* background; thus, the*SlRIPK* coding sequence was fused to the *HA* tag and expressed under the control of the cauliflower mosaic virus 35S promoter in the pGWB14 vector [58]. pGWB5 was used for the overexpressionof *SlRIPK* in tomatoes; thus, the *SlRIPK* coding sequence was fused to the *GFP* tag and expressed under the control of the 35S promoter (Figure S3D, see online supplementary material).BGK012 (Biogle GeneTech) was used to generate the tomato *slripk* mutants using CRISPR/Cas9 gene-editing technology (Figure S3B, see online supplementary material). gRNAs were designed on the website (http://cbi.hzau.edu.cn/CRISPR2/) and those located on exons and near the 5′ end were selected for cloning into the BGK012 vector, which contains the AtU6 promoter for gRNA expression and the hygromycin resistance gene for the selection of positive transformants. *Agrobacterium tumefaciens* strain GV3101 was used for plant transformation. *Arabidopsis* transgenic plants were generated using the floral dip method [59]. Stable transgenic tomatoes were generated using the *Agrobacterium*-mediated cotyledon tissue culture method [37]. Briefly, 8-d-old cotyledon explants were soaked in the bacterial suspension (OD_600_ = 1) for 5 min and then dried on sterilized Whatman paper. After the explants were incubated in the co-cultivation medium (4.54 g/L Murashige and Skoog (MS) salts, 30 g/L sucrose, 100 mg/L phaseomannite, 200 mg/L KH_2_PO_4_, 12.5 μg/L 2, 4-D, 25 μg/L kinetin, 1.3 mg/L thiamine hydrochloride and 5.2 g/L agar, at pH 5.5) for 3 d, they were transferred to the selection medium (4.44 g/L MS salts, 20 g/L sucrose, 100 mg/L phaseomannite, 1.3 mg/L thiamine hydrochloride, 0.1 mg/L zeatin, 4 μg/L Timentin, 1.2 μg/L hygromycin, 7.4 g/L agar, at pH 6). When shoots developed from the explants, they were transferred to the rooting medium (4.44 g/L MS salts, 30 g/L sucrose, 4 μg/L Timentin, 1.2 μg/L hygromycin, 4 g/L agar, pH 6). Two independent lines were selected for each construct, and at least ten homozygous T3 individuals from each line were used for the disease assays.

### Phylogenetic analysis

The amino acid sequences used in this study were downloaded from the National Center for Biotechnology Information (NCBI) database and are listed in Table S3 (see online supplementary material). Multiple sequence alignments were performed using the Clustal W program, and a phylogenetic tree was constructed using an approximation of the maximum-likelihood method.

### RNA isolation and quantitative real-time PCR (qRT-PCR)

Total RNA was extracted using the RNAprep Pure Plant Kit (Tiangen Biotech, Beijing, China) for *Arabidopsis* and Easy Plant RNA Extraction Kit (Easy-Do Biotech, Hangzhou, China) for tomato plants. cDNA was synthesized using HiScript II Reverse Transcriptase (Vazyme Biotech, Nanjing, China). Finally, an Applied Biosystems Plus Real-Time PCR System (ABI, Foster City, CA, USA) was used to perform qRT-PCR using SYBR Green Master Mix (Vazyme Biotech). The relative gene expression levels were calculated using the 2^-∆∆Ct^ method. *ELONGATION FACTOR1α* (*EF1α, Solyc06g00506*) was used as an internal control. All the primers used for qRT-PCR are listed in Table S1 (see online supplementary material).

### Chemiluminescence assay for ROS detection

ROS production in leaf disks was monitored using a luminol-based chemiluminescence assay as previously described [18]. Leaf disks were incubated for 12 h in water under light conditions, and then the levels of ROS were measured using a photon camera (HRPCS5, Photek, East Sussex, UK) after treatment with chitin and pip (Merck KGaA, Darmstadt, Germany). When bacteria were used for the luminol-based assay, the bacterial cells were freshly diluted to the desired concentration as previously described [60].

### Protein extraction and immunoblot analysis

Protein extraction buffer containing 50 mM Tris–HCl (pH 7.5), 150 mM NaCl, 2% Triton X-100, 1 mM phenylmethylsulfonyl fluoride, and 1× protease inhibitor cocktail (Merck KGaA) was used for total protein extraction. Phosphorylated RBOHD was detected using an immunoblot analysis and an α-pS343/347 RBOHD antibody as previously described [18]. Other proteins fused with tags were detected using α-HA (Merck KGaA), α-GFP (Miltenyi Biotec, Bergisch Gladbach, Germany), and α-luciferase (Merck KGaA) antibodies. The loading controls were Ponceau S membrane staining and α-HSP70.

### Confocal microscopy

A confocal microscope (LSM 800; ZEISS, Oberkochen, Germany) was used to detect the subcellular localization of GFP and SlRIPK-GFP proteins in four-week-old leaves from transgenic tomatoes. GFP was excited using a 488 nm filter.

### Split-luciferase assay (SLC) and bimolecular fluorescence complementation assay (BIFC)

This assay was performed as previously described [18]. *Agrobacterium* strain GV3101 containing the corresponding plasmid was injected into *N. benthamiana* leaves and incubated for 24 h. For the SLC assay, 0.5 mM luciferin was brushed onto the leaves and a camera (HRPCS5; Photek) was used to record the subsequent signals for 10 min. For the BIFC assay, a confocal laser-scanning microscope (LSM 800; ZEISS) was used to obtain the images.

### Disease assays

Inoculation with *P. carotovorum*-LuxCDABE was performed as previously described with some modifications [22]. Briefly, the fourth or fifth true leaves of 4-week-old tomato plants were detached and needles were used to make small holes in them. Then, 5 μL bacterial droplets (OD_600_ = 0.6) were added to each hole. Light signals were measured using a photon camera (HRPCS5, Photek) 12 h after inoculation.

For inoculation with *R. solanacearum-*LuxCDABE [38], 10-d-old tomato seedlings were used. Each seedling was placed in a sterile 4 mL tube with its root immersed in the bacterial suspension (OD_600_ = 1.45). Two minutes after inoculation, the seedlings were exposed to air for 5 min, transferred to a new tube, and then incubated with sterile water. Light signals were detected using a photon camera 3 to 4 d post-inoculation.

For infections with *B. cinerea* strain B05.10, the fourth to fifth true leaves of 6-week-old plants were detached and placed in a tray covered with pre-wet filter paper underneath. Each leaf was inoculated with four 2.5 or 5 μL droplets of spore suspension (1 × 10^5^ spores/mL). The tray was covered with plastic film and incubated at 22°C. The size of the infected area was measured using the ImageJ software (https://imagej.nih.gov/ij/) 3 d after inoculation.

Ten-day-old tomato seedlings were infected with *F. oxysporum* f. sp. *Lycopersici* 4287. Each seedling was placed in a sterile 4 mL tube with its roots immersed in a fungal suspension. Disease severity was scored 3 d after inoculation using the following categories: 0, no symptoms; 1, cotyledons began to show signs of wilting; 2, cotyledons completely withered; 3, true leaves began to wither and wilt; and 4, the whole plant wilted and died.

## Acknowledgements

We thank Prof. Yanni Yin (Zhejiang University, China) for providing the *Fusarium* strain and Prof. Dayong Li (Zhejiang University, China) for providing *Botrytis cinerea*. This work was supported by the National Key Research and Development Program of China (2018YFD1000800), National Natural Science Foundation of China (31770263 and 31970279), and the Key Research and Development Program of Zhejiang Province (2021C02064-7, 2021C02009, and 2022C02016).

## Author contributions

R.W. and Y.L. conceived and designed the study. R.W. conducted most of the experiments and analysed the data. C.L., Q.L., and Y.A. helped with pathogen inoculation, X.S. helped with the VIGS experiment, Z.H. helped with phylogenetic analysis, and J.Z. and Y.Z. helped with tomato transformation. R.W. and Y.L. prepared the manuscript. All authors have read and approved the final manuscript.

## Data availability

The data and materials used to support the findings of this study are available from the corresponding author upon request.

## Conflict of interests

The authors declare that they have no conflicts of interest.

## Supplementary data

Supplementary data is available at *Horticulture Research* online.

## Supplementary Material

Web_Material_uhac207
