# Supplementary material for: Tomato receptor-like cytosolic kinase RIPK confers broad-spectrum disease resistance without yield penalties
Source: Hortic Res. 2022 Sep 13;9:uhac207. doi: 10.1093/hr/uhac207 (PMC9715573; doi:10.1093/hr/uhac207)
Supplement: Web_Material_uhac207 [file web_material_uhac207.zip › 3_Supp-rev-submitted-clean.docx]

# Tomato receptor-like cytosolic kinase RIPK confers broad-spectrum disease resistance without yield penalties

**Supplemental information**


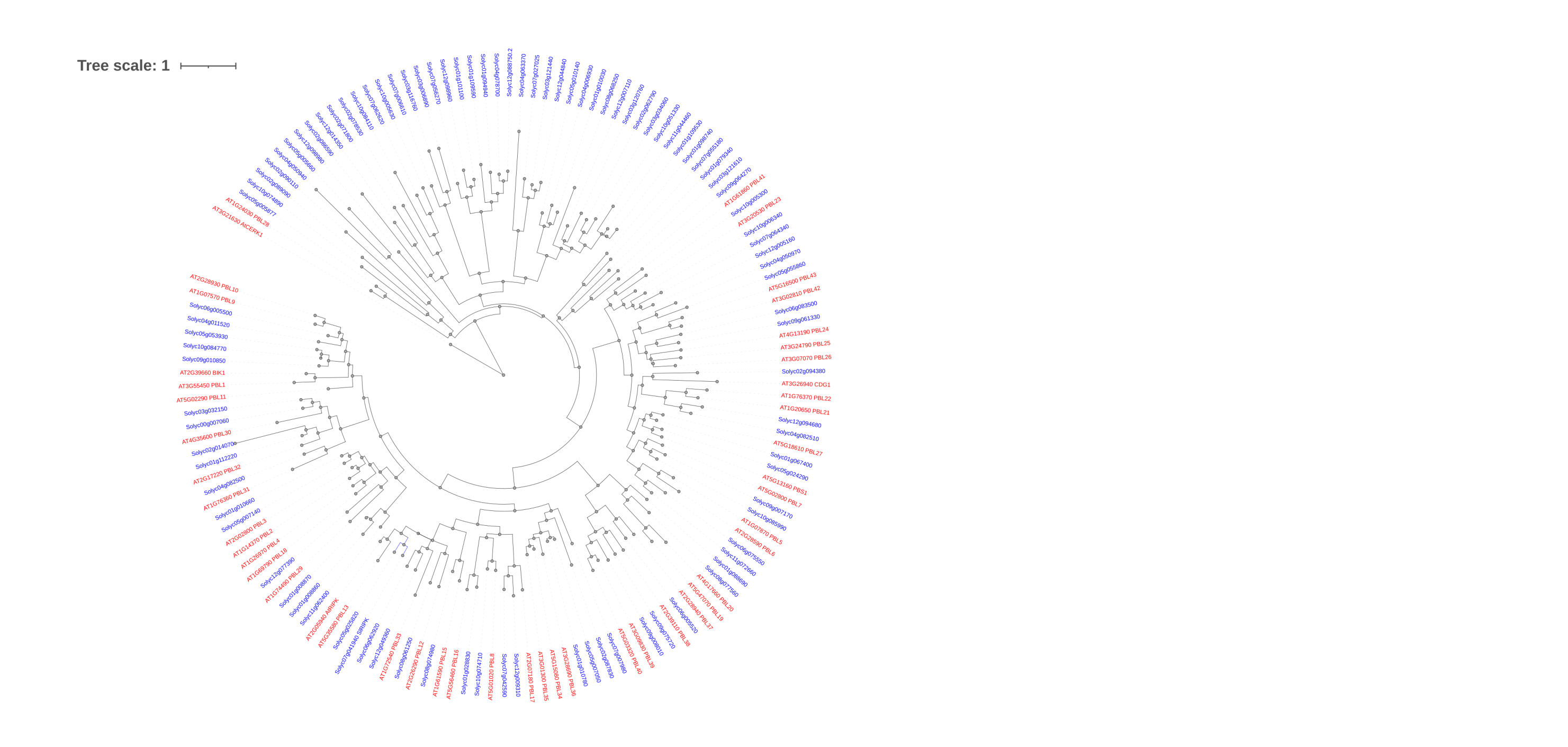


**Supplementary Figure 1. Phylogenetic tree of PBL proteins.** Sequences of PBL proteins from *Arabidopsis thaliana* (AT) and *Solanum lycopersicum* (Solyc) were downloaded from the NCBI database. Alignments and phylogenetic analysis were performed using the MEGA X software with the approximately maximum-likelihood method. The red color represents *A. thaliana*, the blue color represents *S. lycopersicum*.


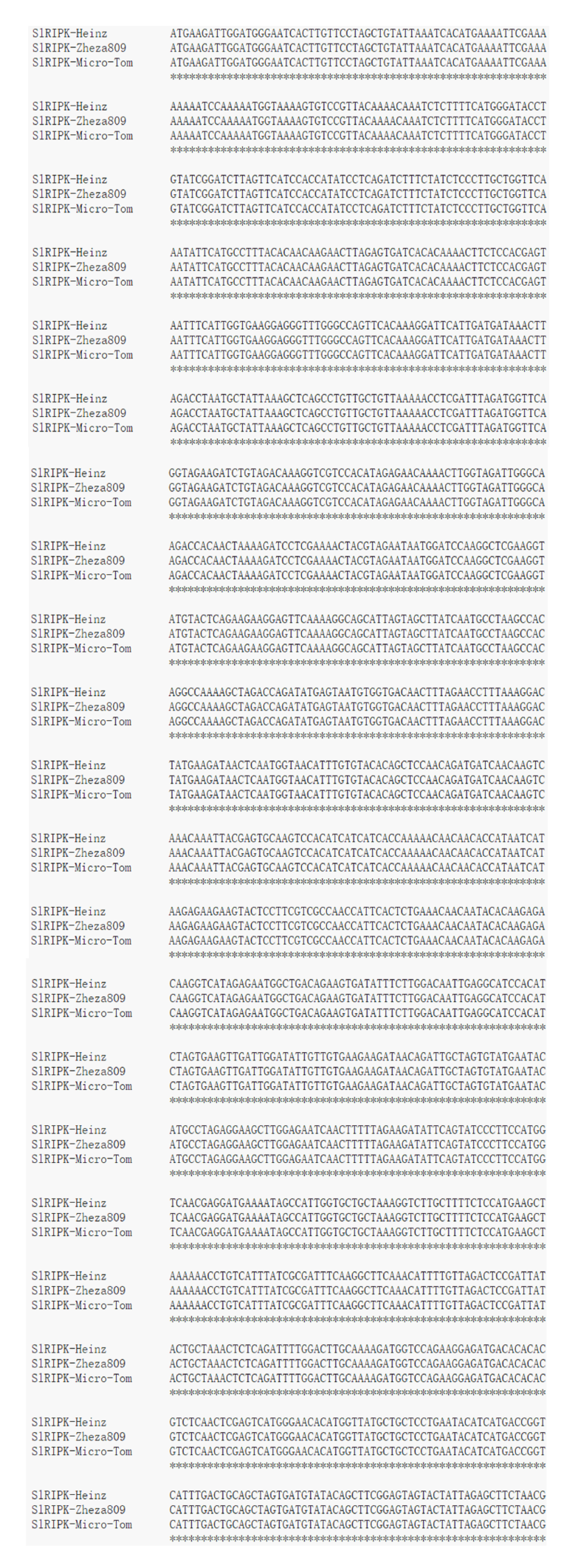


**Supplementary Figure 2.** **Sequence alignment of *SlRIPK* from different tomato cultivars**

The full length *SlRIPK* coding sequences from Micro-Tom and Zheza809 were determined by direct sequencing of PCR products. Heinz genome reference sequences were downloaded from the website (https://phytozome-next.jgi.doe.gov/), and the alignment was performed on the website (https://www.genome.jp/tools-bin/clustalw).


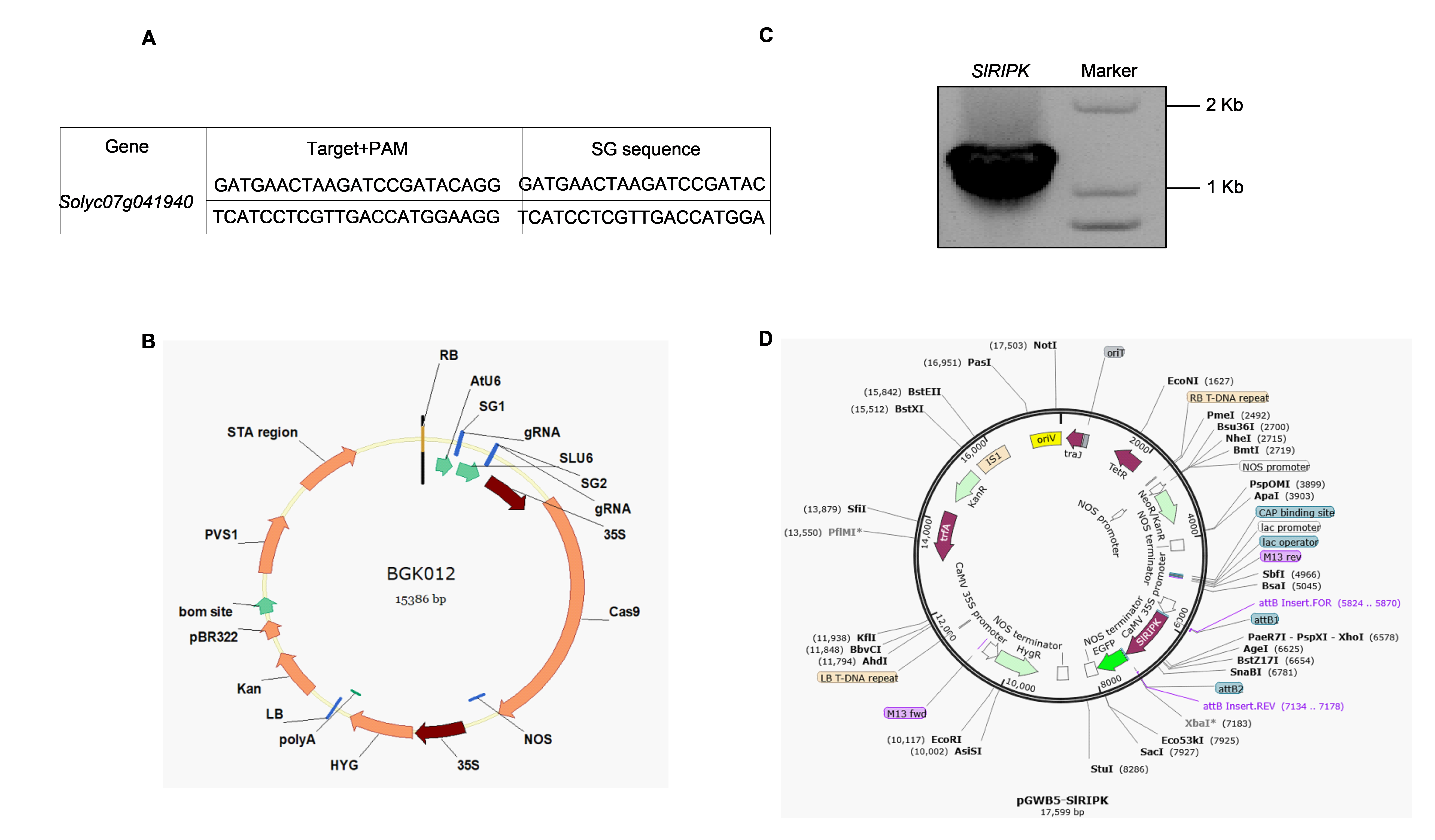


**Supplementary Figure 3.** **Information for plasmid construction**

1. The sequences of guide RNAs (gRNAs) of *SlRIPK* for the CRISPR/Cas9. gRNAs were designed using the website (<http://cbi.hzau.edu.cn/CRISPR2/>), and those are located on exons and near the 5’ end were selected.
2. The plasmid map for *CRISPR/Cas9-SlRIPK-gRNA*. The important elements including *AtU6* promoter, *gRNA*, *Cas9* and *hygromycin resistant gene* (*HYG*) are labelled on the map.
3. The PCR amplification products. Full length *SlRIPK* coding sequences (1302 bp) were amplified by PCR, and then the PCR products were detected by an agarose gel.
4. The plasmid map for *pGWB5-SlRIPK*. The important elements including *cauliflower mosaic virus* *35S* promoter, *SlRIPK,* and *green fluorescence protein* (*GFP*) gene are labelled on the map.


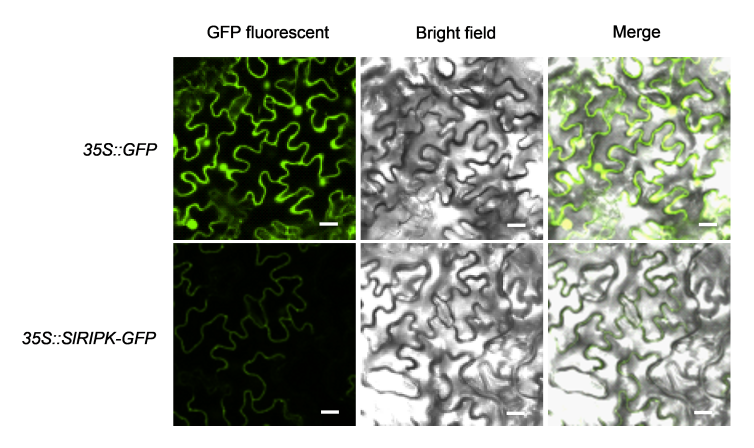


**Supplementary Figure 4.** **SlRIPK localizes to the cell peripheral region**

Green fluorescence was observed in 4-w-old *35S::GFP* and *35S::SlRIPK-GFP* transgenic tomato leaves under a confocal microscope. Bars, 10 µm.


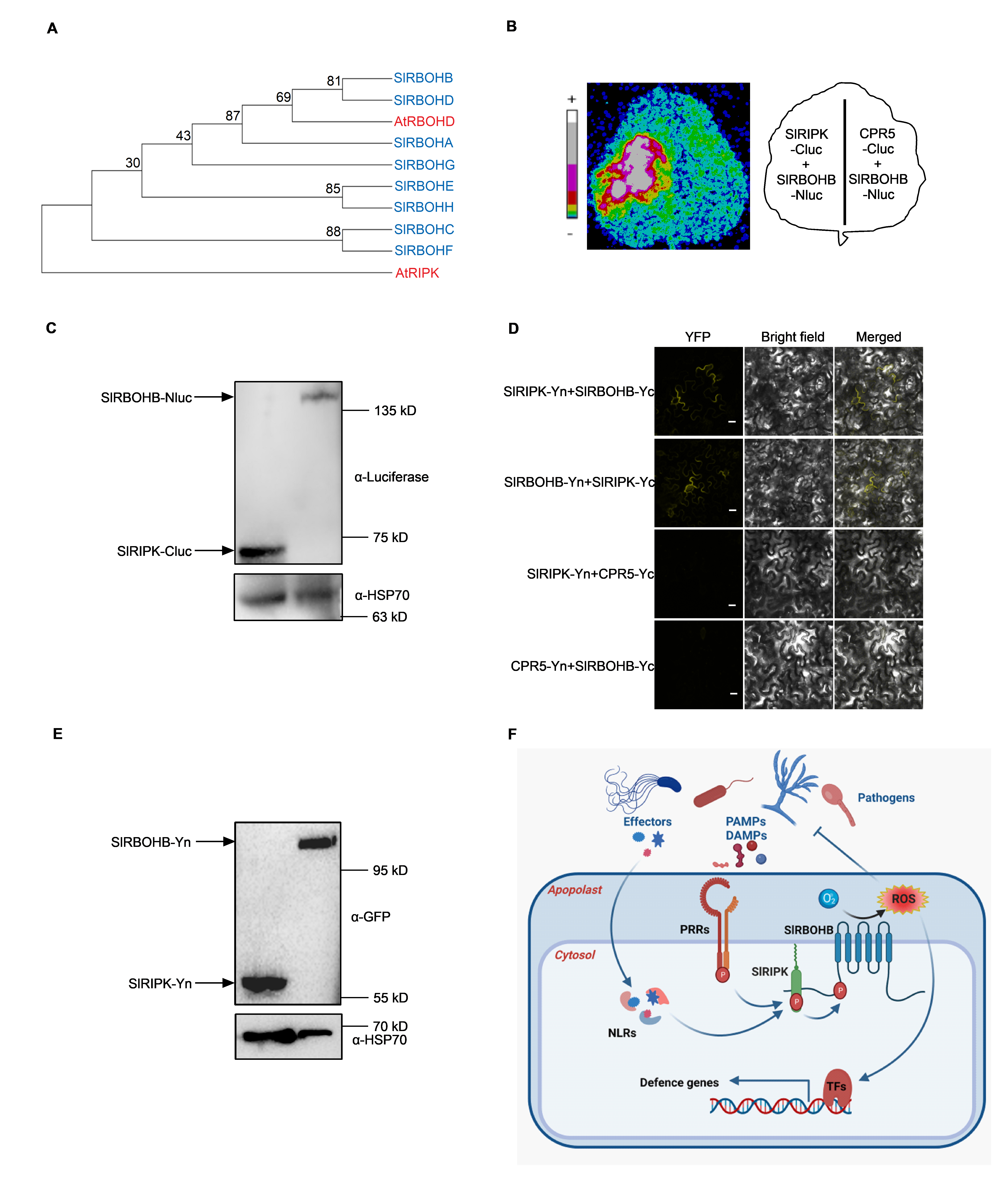


**Supplementary Figure 5. SlRIPK interacts with SlRBOHB**

1. Phylogenetic tree of RBOHD subgroups in *Arabidopsis thaliana* (AT) and *Solanum lycopersicum* (Solyc). The phylogenetic tree was constructed using the maximum likelihood method. The branches are labelled with their respective bootstrap values. Red represents *A. thaliana*, and blue represents *S. lycopersicum*.

**(B, C)** The interaction between SlRIPK and SlRBOHB determined by a split-luciferase complementation assay. SlRIPK and SlRBOHB were fused to the N- or C-terminal portion of luciferase (luc). The fusion proteins were co-expressed in *Nicotiana benthamiana* leaves **(B)**, and their expression was detected by immunoblot analysis using an α-luciferase antibody **(C)**. HSP70 served as a loading control. CPR5-Cluc was used as a negative control and its expression was detected in our previous study (Wu et al., 2022). Images were obtained with a photon camera 3 d after infiltration **(B)**.

**(D, E)** The interaction between SlRIPK and SlRBOHB determined by a bimolecular fluorescence complementation assay. SlRIPK and SlRBOHB were fused to the N- or C-terminal portion of the yellow fluorescence protein (YFP). The fusion proteins were co-expressed in *N. benthamiana* leaves (**D**), and their expression was detected by immunoblot analysis using an α-GFP antibody **(E)**. HSP70 served as a loading control. CPR5-cYFP was used as a negative control and its expression was detected in our previous study (Wu et al., 2022). Images were obtained using a confocal laser scanning microscope 3 d after infiltration **(D)**. Bars, 20 μm.

**(F)** Schematic model. Upon treatment with immune elicitors, SlRIPK activates SlRBOHB to induce the ROS production which increases the plant disease resistance to a range of pathogens.


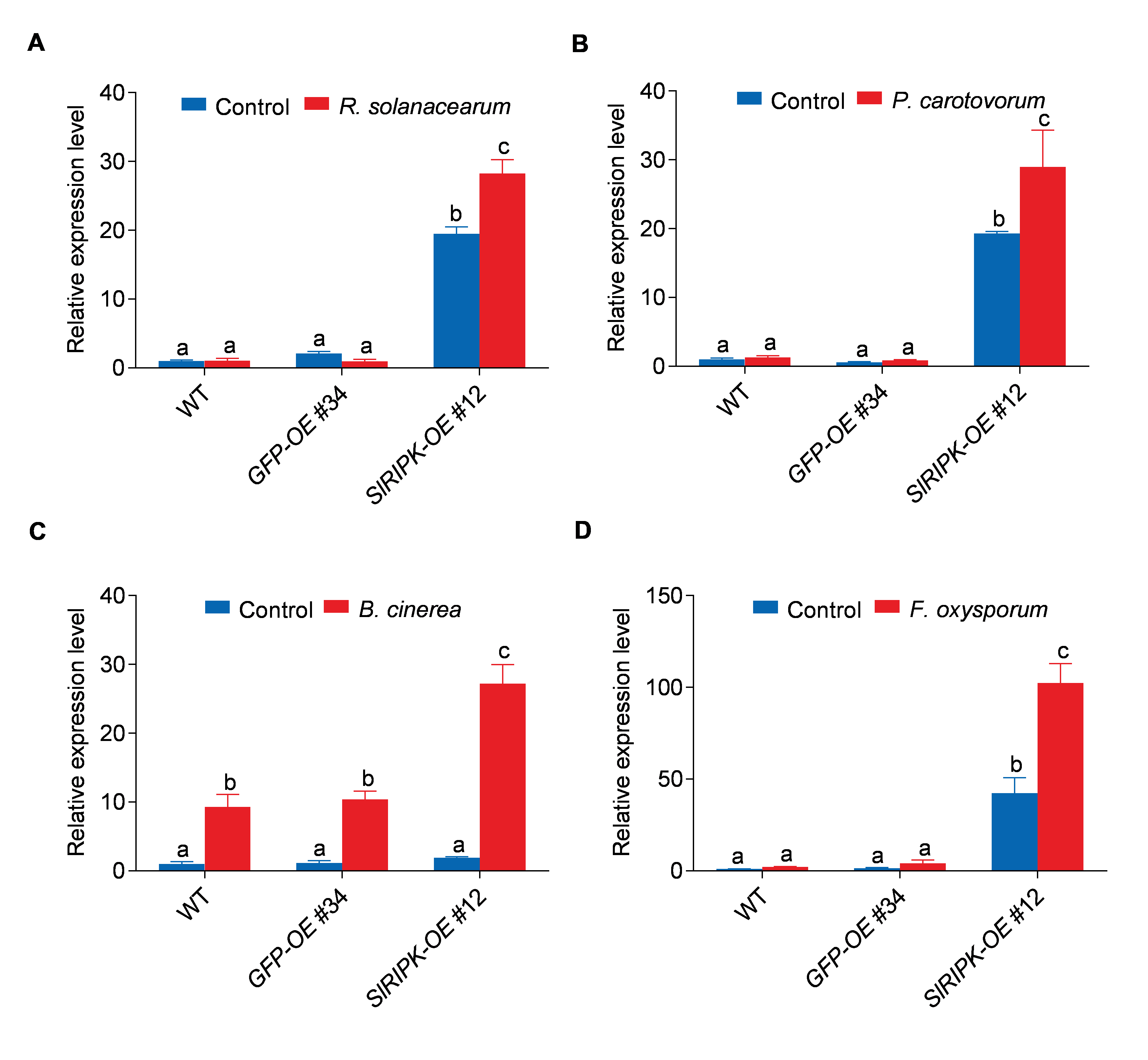


**Supplementary Figure 6. The transcription levels of *SlRIPK* are significantly upregulated in *SlRIPK* overexpression transgenic plants in response to pathogen infection**

1. The relative expression levels of *SlRIPK* after infection with *Ralstonia solanacearum*. RNA was extracted from 4-w-old leaves 2 d after infection with or without *R. solanacearum* (OD_600_ = 0.1) using a root-soaking method.
2. The relative expression levels of *SlRIPK* after infection with *Pectobacterium carotovorum*. RNA was extracted from 4-w-old leaves 3 h after infection with or without *P. carotovorum* (OD_600_ = 0.6).
3. The relative expression levels of *SlRIPK* after infection with *Botrytis cinerea*. RNA was extracted from 4-w-old leaves 12 h after infection with *Botrytis cinerea* spores (1 × 10^5^ spores/mL) or spore suspension solution.
4. The relative expression levels of *SlRIPK* after infection with *Fusarium oxysporum*. RNA was extracted from 10-d-old seedlings 12h after infection with or without *Fusarium oxysporum* (1 × 10^8^ spores/mL).

Gene expression was quantified by qRT-PCR. Data are presented as mean ± SE (n = 3). Different letters above the bars indicate significant differences between different genotypes (*P* ≤  0.05, one-way ANOVA).


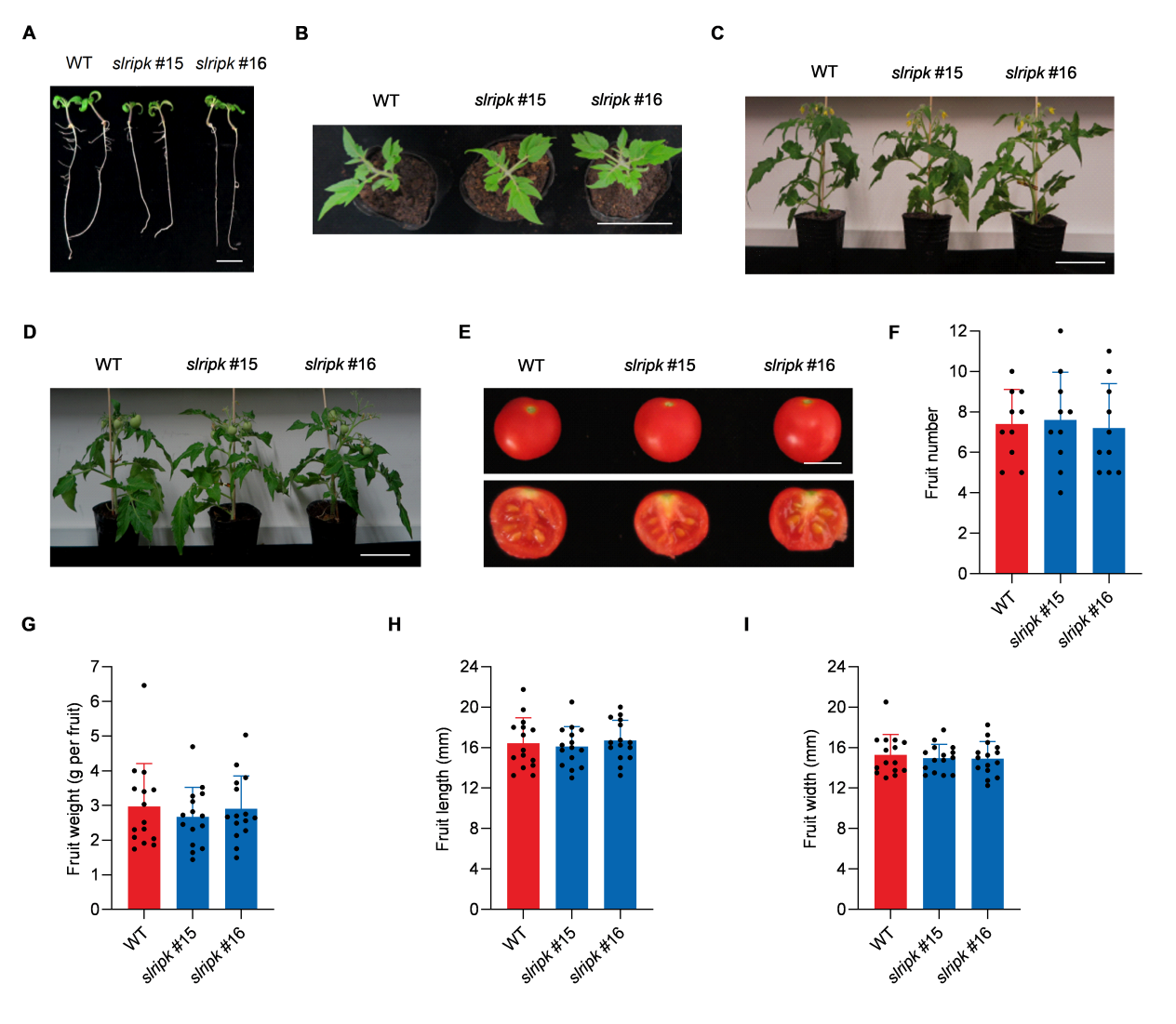


**Supplementary Figure 7. *slripk* mutants show normal growth and reproduction**

1. Representative image of 10-d-old seedlings. Wild-type (WT) and *slripk* mutants were grown on agar medium. Bar, 1 cm.
2. Representative image of one-month-old tomato plants. Ten-day-old WT and *slripk* seedlings from agar media were transferred to pots and grown for another twenty days. Bar, 10 cm.
3. Representative image of two-month-old tomato plants. Bar, 10 cm.
4. Representative image of three-month-old tomato plants. Bar, 10 cm
5. Representative image of four-month-old red-ripened fruits. Bar, 1 cm.
6. Number of fruits per plant. Number of red ripen fruits was measured from four-month-old plants. Data are presented as the mean ± SD (n = 10).
7. Fruit weight. Data are presented as the mean ± SD (n = 15).
8. Fruit length. Data are presented as the mean ± SD (n = 15).
9. Fruit width. Data are presented as the mean ± SD (n = 15).

**Supplementary Table 1.** **Sequences of primers used in this study**

| **Primers** | **Sequences (5'-3')** |
| --- | --- |
| *SlRIPK*-target 1_F | ATGAAGATTGGATGGGAATCAC |
| *SlRIPK*-target 1_R | AATGAATCCTTTGTGAACTGGCC |
| *SlRIPK*-target 2_F | GTTTAGTTACAAAGCAACTATCGG |
| *SlRIPK*-target 2_R | CCATGTGTTCCCATGACTCGAG |
| Cas9(2614)_F | GAAGAGGTCGTGAAGAAGAT |
| Cas9(2614)_R | CGTGGTGGTAGTTGTTGAT |
| hygromycin_F | GATGGACGACACCGTCAGTG |
| hygromycin_R | tgccctcggacgagtgctgg |
| pGWB5-EGFP_F | GACGTGAACGGCCACAAGTT |
| pGWB5-EGFP_R | TTGTAGTTGCCGTCGTCCTT |
| *SlRIPK*-RT-PCR_F | gtcatttgactgcagctagtga |
| *SlRIPK*-RT-PCR_R | GGATCTCTTGAAACCATTTTGC |
| *SlEF-1α*-RT-PCR_F | CGTGAGCGTGGTATCACCATT |
| *SlEF-1α*-RT-PCR_R | GGTAGACCTCTCAATCATGTT |
| *Solyc05g025820*-VIGS-GW_F | GGGGACAAGTTTGTACAAAAAAGCAGGCTACGCTAGACCGGATATGACTACA |
| *Solyc05g025820*-VIGS-GW_R | GGGGACCACTTTGTACAAGAAAGCTGGGTCTTCTTATGTAGACTTTTTGTATTGG |
| *SlRIPK*-VIGS-GW_F | GGGGACAAGTTTGTACAAAAAAGCAGGCTACTAGTAGCTTATCAATGCCTAAGCC |
| *SlRIPK*-VIGS-GW_R | GGGGACCACTTTGTACAAGAAAGCTGGGTCCCATTTTGCAATGGTGAATT |
| *Solyc08g061250*-VIGS-GW_F | GGGGACAAGTTTGTACAAAAAAGCAGGCTACTCTGTTGGAACTGCTAACTG |
| *Solyc08g061250*-VIGS-GW_R | GGGGACCACTTTGTACAAGAAAGCTGGGTCTCAATGTCTTCGATAGAGAG |
| *Solyc06g062920*-VIGS-GW_F | GGGGACAAGTTTGTACAAAAAAGCAGGCTACATGGCTACTTGCGGAATTGACTGG |
| *Solyc06g062920*-VIGS-GW_R | GGGGACCACTTTGTACAAGAAAGCTGGGTCCCCCTTGTGAACGGGTCCAA |
| *Solyc12g049360*-VIGS-GW_F | GGGGACAAGTTTGTACAAAAAAGCAGGCTACTGTGGTCAAGACATTGGAAC |
| *Solyc12g049360*-VIGS-GW_R | GGGGACCACTTTGTACAAGAAAGCTGGGTCTTAAATATTTGTAGCAGACTTTTG |
| *Solyc05g025820*-VIGS-qRT-PCR_F | GTAGCTTACCAATGCTTAAGCC |
| *Solyc05g025820*-VIGS-qRT-PCR_R | GGACTTCGTTTGTGTTGATCAA |
| *SlRIPK*-VIGS-qRT-PCR_F | AACAAATTACGAGTGCAAGTCC |
| *SlRIPK*-VIGS-qRT-PCR_R | AATAATGTGGACGTTGCGTAAC |
| *Solyc08g061250*-VIGS-qRT-PCR_F | GTTATCGGCTGATGAACTCTCT |
| *Solyc08g061250*-VIGS-qRT-PCR_R | CTTGTGACCTTGTAGACCATCT |
| *Solyc06g062920*-VIGS-qRT-PCR_F | GAGTCGGACACAGGTATAAACA |
| *Solyc06g062920*-VIGS-qRT-PCR_R | CCTTTCTTCTATTCGTTGCGAT |
| *Solyc12g049360*-VIGS-qRT-PCR_F | CCGAGTACATGATGACTGGTTA |
| *Solyc12g049360*-VIGS-qRT-PCR_R | ATTATTCGTTCGAGCTTGTGTG |
| *SlEF-1α*-qRT-PCR_F | GGAACTTGAGAAGGAGCCTAAG |
| *SlEF-1α*-q RT-PCR_R | gacaacaccgacagcaacagtc |
| *SlRIPK*-CDS-GW_F | GGGGACAAGTTTGTACAAAAAAGCAGGCTACATGAAGATTGGATGGGAATC |
| *SlRIPK*-CDS-GW_R | GGGGACCACTTTGTACAAGAAAGCTGGGTCGGATCTCTTGAAACCATTTT |

**Supplementary Table 2. Information for PCR used in this study**

| **Purpose of the PCR** | **Primers of the PCR** | **Sequences of the PCR product (5'-3')** | **Size of the PCR product** |
| --- | --- | --- | --- |
| Identification of *slripk* individuals with mutation in the first target  (genomic DNA as template) | *SlRIPK*-target1_F/R | atgaagattggatgggaatcacttgttcctagctgtattaaatcacatgaaaattcgaaaaaaaatccaaaaatggtaaaagtgtccgttacaaaacaaatctcttttcatgggatacctgtatcggatcttagttcatccaccatatcctcagatctttctatctcccttgctggttcaaatattcatgcctttacacaacaagaacttagagtgatcacacaaaacttctccacgagtaatttcattggtgaaggagggtttgggccagttcacaaaggattcatt | 288 bp |
| Identification of *slripk* individuals with mutation in the second target  (genomic DNA as template) | *SlRIPK*-target2_F/R | gtttagttacaaagcaactatcggttttgtacgtagaaagtcaaaatatatataatactgaattttttaaaaataatttgcaggatattcagtatcccttccatggtcaacgaggatgaaaatagccattggtgctgctaaaggtcttgcttttctccatgaagctaaaaaacctgtcatttatcgcgatttcaaggcttcaaacattttgttagactccgtaagtagtaatttcaatatatagatagatattgtgctatatatattttgagtaaattaaaaccctaattttgttgaacaggattatactgctaaactctcagattttggacttgcaaaagatggtccagaaggagatgacacacacgtctcaactcgagtcatgggaacacatgg | 396 bp |
| Identification of positive transformants of *slripk* by amplifying the *Cas9* fragment  (genomic DNA as template) | Cas9 (2614)_F/R | gaagaggtcgtgaagaagatgaagaactactggcggcagctgctgaacgccaagctgattacccagagaaagttcgacaatctgaccaaggccgagagaggcggcctgagcgaactggataaggccggcttcatcaagagacagctggtggaaacccggcagatcacaaagcacgtggcacagatcctggactcccggatgaacactaagtacgacgagaatgacaagctgatccgggaagtgaaagtgatcaccctgaagtccaagctggtgtccgatttccggaaggatttccagttttacaaagtgcgcgagatcaacaactaccaccacg | 334 bp |
| Identification of positive transformants by amplifying the hygromycin fragment  (genomic DNA as template) | hygromycin_F/R | gatggacgacaccgtcagtgcgtccgtcgcgcaggctctcgatgagctgatgctttgggccgaggactgccccgaagtccggcacctcgtgcacgcggatttcggctccaacaatgtcctgacggacaatggccgcataacagcggtcattgactggagcgaggcgatgttcggggattcccaatacgaggtcgccaacatcttcttctggaggccgtggttggcttgtatggagcagcagacgcgctacttcgagcggaggcatccggagcttgcaggatcgccacgactccgggcgtatatgctccgcattggtcttgaccaactctatcagagcttggttgacggcaatttcgatgatgcagcttgggcgcagggtcgatgcgacgcaatcgtccgatccggagccgggactgtcgggcgtacacaaatcgcccgcagaagcgcggccgtctggaccgatggctgtgtagaagtactcgccgatagtggaaaccgacgccccagcactcgtccgagggca | 523 bp |
| Identification of positive transformants of *RIPK-OE* by amplifying the *GFP* fragment  (genomic DNA as template) | pGWB5-EGFP_F/R | gacgtgaacggccacaagttcagcgtgtccggcgagggcgagggcgatgccacctacggcaagctgaccctgaagttcatctgcaccaccggcaagctgcccgtgccctggcccaccctcgtgaccaccttcacctacggcgtgcagtgcttcagccgctaccccgaccacatgaagcagcacgacttcttcaagtccgccatgcccgaaggctacgtccaggagcgcaccatcttcttcaaggacgacggcaactacaa | 260 bp |
| Determination of transcript levels of *SlRIPK* by RT-PCR  (cDNA as template) | *SlRIPK*-RT-PCR_F/R | gtcatttgactgcagctagtgatgtatacagcttcggagtagtactattagagcttctaacgggtagaagatctgtagacaaaggtcgtccacatagagaacaaaacttggtagattgggcaagaccacaactaaaagatcctcgaaaactacgtagaataatggatccaaggctcgaaggtatgtactcagaagaaggagttcaaaaggcagcattagtagcttatcaatgcctaagccacaggccaaaagctagaccagatatgagtaatgtggtgacaactttagaacctttaaaggactatgaagataactcaatggtaacatttgtgtacacagctccaacagatgatcaacaagtcaaacaaattacgagtgcaagtccacatcatcatcaccaaaaacaacaacaccataatcataagagaagaagtactccttcgtcgccaaccattcactctgaaacaacaatacacaagagattaactccaaattcaccattgcaaaatggtttcaagagatcc | 524 bp |
| Determination of reference gene level of *SlEF-1α* by RT-PCR  (cDNA as template) | *SlEF-1α*-RT-PCR_F/R | cgtgagcgtggtatcaccattgatattgctttgtggaagtttgagaccactaagtactactgcactgttattgatgcccccggccacagggatttcatcaagaacatgatcactggtacctctcaggctgactgtgctgttctcattattgactccactactggtggttttgaagctggtatctccaaagatggtcagacccgtgaacatgcattgcttgctttcacccttggtgtcaagcaaatgatctgctgctgtaacaagatggatgctaccacccccaagtactccaaggctaggtatgatgaaatcgtgaaggaagtttcttcctacctcaagaaggttggttacaaccctgacaaaatcccctttgttccaatctctggttttgaaggagacaacatgattgagaggtctacc | 420 bp |
| Amplification of the fragment of *Solyc05g025820* for VIGS  (cDNA as template) | *Solyc05g025820*-VIGS-GW_F/R | GCTAGACCGGATATGACTACAGTTGTCAAAACACTTGAAACATTGAAGGATTATAAGGATATTTCAACAATGACATTTGTCTATATAGCTCCAGTACTTGATCAACACAAACGAAGTCCTCAAAGGGAATTACTCAACAAAACAAATACCAATACAAAAAGTCTACATAAGAA | 173 bp |
| Amplification of the fragment of *SlRIPK* for VIGS  (cDNA as template) | *SlRIPK*-VIGS-GW_F/R | tagtagcttatcaatgcctaagccacaggccaaaagctagaccagatatgagtaatgtggtgacaactttagaacctttaaaggactatgaagataactcaatggtaacatttgtgtacacagctccaacagatgatcaacaagtcaaacaaattacgagtgcaagtccacatcatcatcaccaaaaacaacaacaccataatcataagagaagaagtactccttcgtcgccaaccattcactctgaaacaacaatacacaagagattaactccaaattcaccattgcaaaatgg | 295 bp |
| Amplification of the fragment of *Solyc08g061250* for VIGS  (cDNA as template) | *Solyc08g061250*-VIGS-GW_F/R | TCTGTTGGAACTGCTAACTGGTAAAAGGTCTTTGGATAAGTCGAGAAGAGAAGGAGAACATAATTTAGTTGAATGGTTAAGGCCTTATCTAAGAGATCCAAAGAGAATTGCTCGTGTGATGGATCGAAGACTTGAAGATGAATACCCTATGAAAGGGGCACAAACTGCAGCATTAGTAGCATACAAATGTTTAAATCACTATCCTAAGCCTAGGCCTACAATGGATGATGTAGTCAAGATTCTCGAAACTCTTCAAGATGAAAACAACAACATCGACACTTCAATCAGTGATCCAATGATAACGATGACATTGAGTAGTGATTTTAGCAGTGGAAGTGAGCAAAATGAGGATACTGCAGCACCAGAAAGGAATAGAAATAACAAGTACTTGAATAATGAGAGAAATCAAGGTTATGGCTGGAAACATCGACTCAATAGACAACGAATGGTGGCATCATACTCAGATACAGCTCTCTATCGAAGACATTGA | 490 bp |
| Amplification of the fragment of *Solyc06g062920* for VIGS  (cDNA as template) | *Solyc06g062920*-VIGS-GW_F/R | ATGGCTACTTGCGGAATTGACTGGAAATCTGTTCTGCCAAACTGTTTTAAGGGCAATAATGTTCGTTCGGAGGCGAAGGTGATGGAGAACAGTAAACAGATGAATTCTGATCATCATAGATTAGCTTTTTCTGATATAAGTACTGATTCTAGATCAGTACTTATATCATTGGATGACCTTTCATCGAACGCTGTCATTGGTTCAAATCTTCATGTATTCACATATGAGGAACTTAAACTCATCACTAGTGATTTCTCCTCAGCTAATTTTCTCGGTAAAGGTGGATTTGGACCCGTTCACAAGGGG | 306 bp |
| Amplification of the fragment of *Solyc12g049360* for VIGS  (cDNA as template) | *Solyc12g049360*-VIGS-GW_F/R | TGTGGTCAAGACATTGGAACCTATTTTGGACTTGAAAGATATACCAATTGGCTCATTTGTTTATGTGGTTCCCTCATTTGACTCTAAAAGTGGTTTGAAGACAAAAGGGAATGAAGAAAATAAGATGCATATAATAAGTGATAAAAATCATGACAAAGAAAATGCAAGGGAAATGAATCAACAAAAGTCTGCTACAAATATTTAA | 205 bp |
| qRT-PCR for *Solyc05g025820*  (cDNA as template) | *Solyc05g025820*-VIGS-qRT-PCR_F/R | GTAGCTTACCAATGCTTAAGCCATAGGCCAAAAGCTAGACCGGATATGACTACAGTTGTCAAAACACTTGAAACATTGAAGGATTATAAGGATATTTCAACAATGACATTTGTCTATATAGCTCCAGTACTTGATCAACACAAACGAAGTCC | 152 bp |
| qRT-PCR for *SlRIPK*  (cDNA as template) | *SlRIPK*-VIGS-qRT-PCR_F/R | AACAAATTACGAGTGCAAGTCCACATCATCATCACCAAAAACAACAACACCATAATCATAAGAGAAGAAGTACTCCTTCGTCGCCAACCATTCACTCTGAAACAACAATACACAAGAGATTAACTCCAAATTCACCATTGCAAAATGGTTTCAAGAGATCCTAGATGAAATAAATTATTATCAAATTGTATATGATATTTAGATAGAGAATCGTATTAATTCGTTACGCAACGTCCACATTATT | 244 bp |
| qRT-PCR for *Solyc08g061250*  (cDNA as template) | *Solyc08g061250*-VIGS-qRT-PCR_F/R | GTTATCGGCTGATGAACTCTCTAATTCATTCATTGGATCAAGCCTAATAAACTTTACATTTACTGATCTTAGAGAGGTAACACATAATTTTTCTTCTGCTAATTTTCTTGGTGAAGGAGGATTTGGACCTGTGTACAAGGGATTCGTCGATGACAAAGTTAGACCTGGATTGAAAGCTCAGGTTGTGGCTGTTAAGGTGCTGGATACAGATGGTCTACAAGGTCACAAG | 229 bp |
| qRT-PCR for *Solyc06g062920*  (cDNA as template) | *Solyc06g062920*-VIGS-qRT-PCR_F/R | GAGTCGGACACAGGTATAAACACAGGCTAAAGACTGATGCTTCTGTTTACTCAGATACTCATTTGTATCACAAAACTGTAAAGCATGAAAGAACAAACAAACTAAATTCTTATTGATCAATCGCAACGAATAGAAGAAAGG | 141 bp |
| qRT-PCR for *Solyc12g049360*  (cDNA as template) | *Solyc12g049360*-VIGS-qRT-PCR_F/R | CCGAGTACATGATGACTGGTTATTTGACAACTAGGAGCGATGTATATAGTTATGGAGTTGTCTTGTTAGAATTACTAACAGGAAGACAGGCGATTGACAAAAAACGTGGTAGTAGGGAGCAAAACTTGGTGGAATGGGCTAAGCCGTTCCTCCGAGATTCACACAAGCTCGAACGAATAAT | 181 bp |
| qRT-PCR for *SlEF-1α*  (cDNA as template) | *SlEF-1α*-qRT-PCR_F/R | ggaacttgagaaggagcctaagttcttgaagaacggtgatgctggtatggttaagatgattcccaccaagcccatggttgttgagacctttgctgaataccctccattgggtcgttttgctgtgagggacatgaggcagactgttgctgtcggtgttgtc | 160 bp |
| Construction of plasmid *pGWB14/5-SlRIPK*  (cDNA as template) | *SlRIPK*-CDS-GW_F/R | atgaagattggatgggaatcacttgttcctagctgtattaaatcacatgaaaattcgaaaaaaaatccaaaaatggtaaaagtgtccgttacaaaacaaatctcttttcatgggatacctgtatcggatcttagttcatccaccatatcctcagatctttctatctcccttgctggttcaaatattcatgcctttacacaacaagaacttagagtgatcacacaaaacttctccacgagtaatttcattggtgaaggagggtttgggccagttcacaaaggattcattgatgataaacttagacctaatgctattaaagctcagcctgttgctgttaaaaacctcgatttagatggttcacaaggtcatagagaatggctgacagaagtgatatttcttggacaattgaggcatccacatctagtgaagttgattggatattgttgtgaagaagataacagattgctagtgtatgaatacatgcctagaggaagcttggagaatcaactttttagaagatattcagtatcccttccatggtcaacgaggatgaaaatagccattggtgctgctaaaggtcttgcttttctccatgaagctaaaaaacctgtcatttatcgcgatttcaaggcttcaaacattttgttagactccgattatactgctaaactctcagattttggacttgcaaaagatggtccagaaggagatgacacacacgtctcaactcgagtcatgggaacacatggttatgctgctcctgaatacatcatgaccggtcatttgactgcagctagtgatgtatacagcttcggagtagtactattagagcttctaacgggtagaagatctgtagacaaaggtcgtccacatagagaacaaaacttggtagattgggcaagaccacaactaaaagatcctcgaaaactacgtagaataatggatccaaggctcgaaggtatgtactcagaagaaggagttcaaaaggcagcattagtagcttatcaatgcctaagccacaggccaaaagctagaccagatatgagtaatgtggtgacaactttagaacctttaaaggactatgaagataactcaatggtaacatttgtgtacacagctccaacagatgatcaacaagtcaaacaaattacgagtgcaagtccacatcatcatcaccaaaaacaacaacaccataatcataagagaagaagtactccttcgtcgccaaccattcactctgaaacaacaatacacaagagattaactccaaattcaccattgcaaaatggtttcaagagatcc | 1302 bp |

**Supplementary Table 3. The amino acid sequences for phylogenetic analysis**

| **Gene name** | **Amino acid sequences** |
| --- | --- |
| AT3G55450 PBL1 | MGSCLSSRVLSTFSTILHLPFFFFFFFFPFFGVLSILTVVNAFVCCADKSSSGLDDLHLSSCKSSSSATAHKTEGEILSSTTVKSFSFNELKLATRNFRSDSVVGEGGFGCVFRGWLDETTLTPTKSSSGLVIAVKRLNPDGFQGHREWLTEINYLGQLSHPNLVKLIGYCLEDEQRLLVYEFMHKGSLENHLFANGNKDFKPLSWILRIKVALDAAKGLAFLHSDPVKVIYRDIKASNILLDSDFNAKLSDFGLARDGPMGEQSYVSTRVMGTFGYAAPEYVSTGHLNARSDVYSFGVVLLELLCGRQALDHNRPAKEQNLVDWARPYLTSRRKVLLIVDTRLNSQYKPEGAVRLASIAVQCLSFEPKSRPTMDQVVRALVQLQDSVVKPANVDPLKVKDTKKLVGLKTEDKYQRNGLNKKTVGL |
| AT1G14370 PBL2 | MGNCLDSSAKVDNSNHSPHANSASSGSKVSSKTSRSTGPSGLSTTSYSTDSSFGPLPTLRTEGEILSSPNLKAFTFNELKNATKNFRQDNLLGEGGFGCVFKGWIDQTSLTASRPGSGIVVAVKQLKPEGFQGHKEWLTEVNYLGQLSHPNLVLLVGYCAEGENRLLVYEFMPKGSLENHLFRRGAQPLTWAIRMKVAVGAAKGLTFLHEAKSQVIYRDFKAANILLDADFNAKLSDFGLAKAGPTGDNTHVSTKVIGTHGYAAPEYVATGRLTAKSDVYSFGVVLLELISGRRAMDNSNGGNEYSLVDWATPYLGDKRKLFRIMDTKLGGQYPQKGAFTAANLALQCLNPDAKLRPKMSEVLVTLEQLESVAKPGTKHTQMESPRFHHSSVMQKSPVRYSHDRPLLHMTPGASPLPSYTQSPRVR |
| AT2G02800 PBL3 | MGNCLDSSAKVDSSSHSPHANSASLSSRVSSKTSRSTVPSSLSINSYSSVESLPTPRTEGEILSSPNLKAFTFNELKNATRNFRPDSLLGEGGFGYVFKGWIDGTTLTASKPGSGIVVAVKKLKTEGYQGHKEWLTEVNYLGQLSHPNLVKLVGYCVEGENRLLVYEFMPKGSLENHLFRRGAQPLTWAIRMKVAIGAAKGLTFLHDAKSQVIYRDFKAANILLDAEFNSKLSDFGLAKAGPTGDKTHVSTQVMGTHGYAAPEYVATGRLTAKSDVYSFGVVLLELLSGRRAVDKSKVGMEQSLVDWATPYLGDKRKLFRIMDTRLGGQYPQKGAYTAASLALQCLNPDAKLRPKMSEVLAKLDQLESTKPGTGVGNRQAQIDSPRGSNGSIVQKSPRRYSYDRPLLHITPGASPLPTHNHSPRVR |
| AT1G26970 PBL4 | MGNCFGFSAKVGNRESPYRGSSRISAKRSQSSRLSSLTIQSSSYNDDTSVASLQTPRSEGELLASPTLKAFTFNELKTATRNFRPDSVIGEGGFGYVYKGWIDERTLSPSKPGSGMVVAVKKLKEEGFQGHRQWLAEVDCLGRLHHMNLVKLIGYCSKGDHIRLLVYEYMPKGSLENHLFRRGAEPIPWRTRIKVAIGAARGLAFLHEAQVIYRDFKASNILLDSEFNAKLSDFGLAKVGPTGDRTHVSTQVMGTQGYAAPEYVATGRITAKSDVYSFGVVLLELLSGRLTVDKTKVGVERNLVDWAIPYLGDKRKVFRIMDTKLGGQYPHKGACLTANTALQCLNQEPKLRPKMSDVLSTLEELEMTLKSGSISNSVMKLTSSSSSFTAKQRVRTPVADPVLSSRRCRRVR |
| AT1G07870 PBL5 | MGCFGCSKKSSKRSETNKDTVINRKIVGGTTSVAKSDKRDDQTQPSSDSTKVSPYRDVNNEGGVGKEDQLSLDVKGLNLNDQVTGKKAQTFTFQELAEATGNFRSDCFLGEGGFGKVFKGTIEKLDQVVAIKQLDRNGVQGIREFVVEVLTLSLADHPNLVKLIGFCAEGDQRLLVYEYMPQGSLEDHLHVLPSGKKPLDWNTRMKIAAGAARGLEYLHDRMTPPVIYRDLKCSNILLGEDYQPKLSDFGLAKVGPSGDKTHVSTRVMGTYGYCAPDYAMTGQLTFKSDIYSFGVVLLELITGRKAIDNTKTRKDQNLVGWARPLFKDRRNFPKMVDPLLQGQYPVRGLYQALAISAMCVQEQPTMRPVVSDVVLALNFLASSKYDPNSPSSSSGKNPSFHRDRDDEEKRPHLVKETECEGRTGNTRRGIFPVSEYWIHAIPVIVFLCFFTLWIFSHSVSVMNDGEIMSIHRLEKSMAVRNESHVSLAILASSAVSPASSALVAVVSTNQNLTTFHNATQSQQNATQSVNKAKQPHAV |
| AT2G28590 PBL6 | MGCFGRTPKSNKRSDTKTTKNNDFTPKKLTVNANRDKLTQPSSDCLKVSICGDVSKEIVTKKDQLALDAKDTNVEDEVIVKKAQTFTFEELSVSTGNFKSDCFLGEGGFGKVYKGFIEKINQVVAIKQLDRNGAQGIREFVVEVLTLSLADHPNLVKLIGFCAEGVQRLLVYEYMPLGSLDNHLHDLPSGKNPLAWNTRMKIAAGAARGLEYLHDTMKPPVIYRDLKCSNILIDEGYHAKLSDFGLAKVGPRGSETHVSTRVMGTYGYCAPDYALTGQLTFKSDVYSFGVVLLELITGRKAYDNTRTRNHQSLVEWANPLFKDRKNFKKMVDPLLEGDYPVRGLYQALAIAAMCVQEQPSMRPVIADVVMALDHLASSKYDRSHRQKQDNVTETKVDEEKTLTTESNVCVEEKQEIKICSDQAT |
| AT5G02800 PBL7 | MGWIPCSGKSSGRNKTRRNGDHKLDRKSSDCSVSTSEKSRAKSSLSESKSKGSDHIVAQTFTFSELATATRNFRKECLIGEGGFGRVYKGYLASTSQTAAIKQLDHNGLQGNREFLVEVLMLSLLHHPNLVNLIGYCADGDQRLLVYEYMPLGSLEDHLHDISPGKQPLDWNTRMKIAAGAAKGLEYLHDKTMPPVIYRDLKCSNILLDDDYFPKLSDFGLAKLGPVGDKSHVSTRVMGTYGYCAPEYAMTGQLTLKSDVYSFGVVLLEIITGRKAIDSSRSTGEQNLVAWARPLFKDRRKFSQMADPMLQGQYPPRGLYQALAVAAMCVQEQPNLRPLIADVVTALSYLASQKFDPLAQPVQGSLFAPGTPPRSKRV |
| AT5G01020 PBL8 | MGNCGTRDEAAVFTPQAQAQQLQKKHSRSVSDLSDPSTPRFRDDSRTPISYAQVIPFTLFELETITKSFRPDYILGEGGFGTVYKGYIDDNLRVGLKSLPVAVKVLNKEGLQGHREWLTEVNFLGQLRHPNLVKLIGYCCEDDHRLLVYEFMLRGSLENHLFRKTTAPLSWSRRMMIALGAAKGLAFLHNAERPVIYRDFKTSNILLDSDYTAKLSDFGLAKAGPQGDETHVSTRVMGTYGYAAPEYVMTGHLTARSDVYSFGVVLLEMLTGRKSVDKTRPSKEQNLVDWARPKLNDKRKLLQIIDPRLENQYSVRAAQKACSLAYYCLSQNPKARPLMSDVVETLEPLQCTGDALIPCATTTAGAAFAMGGVPDYRMHRRFAKNVGPGAICRSPNPNYSPGGPAACRVR |
| AT1G07570 PBL9 | MLFPITNYEPSRRNLLASVPLLFCQGASTKYDAKDIGSLGSKASSVSVRPSPRTEGEILQSPNLKSFSFAELKSATRNFRPDSVLGEGGFGCVFKGWIDEKSLTASRPGTGLVIAVKKLNQDGWQGHQEWLAEVNYLGQFSHRHLVKLIGYCLEDEHRLLVYEFMPRGSLENHLFRRGLYFQPLSWKLRLKVALGAAKGLAFLHSSETRVIYRDFKTSNILLDSEYNAKLSDFGLAKDGPIGDKSHVSTRVMGTHGYAAPEYLATGHLTTKSDVYSFGVVLLELLSGRRAVDKNRPSGERNLVEWAKPYLVNKRKIFRVIDNRLQDQYSMEEACKVATLSLRCLTTEIKLRPNMSEVVSHLEHIQSLNAAIGGNMDKTDRRMRRRSDSVVSKKVNAGFARQTAVGSTVVAYPRPSASPLYV |
| AT2G28930 PBL10 | MKMGFVEKVKSNVFLYANYVFGCCIGASPKYMSSEANDSLGSKSSSVSIRTNPRTEGEILQSPNLKSFTFAELKAATRNFRPDSVLGEGGFGSVFKGWIDEQTLTASKPGTGVVIAVKKLNQDGWQGHQEWLAEVNYLGQFSHPNLVKLIGYCLEDEHRLLVYEFMPRGSLENHLFRRGSYFQPLSWTLRLKVALGAAKGLAFLHNAETSVIYRDFKTSNILLDSEYNAKLSDFGLAKDGPTGDKSHVSTRIMGTYGYAAPEYLATGHLTTKSDVYSYGVVLLEVLSGRRAVDKNRPPGEQKLVEWARPLLANKRKLFRVIDNRLQDQYSMEEACKVATLALRCLTFEIKLRPNMNEVVSHLEHIQTLNEAGGRNIDMVQRRMRRRSDSVAINQKPNAGFARQTAVGVIATAYPRPSDSPLFV |
| AT5G02290 PBL11 | MGGCFSNRIKTDIASSTWLSSKFLSRDGSKGSSTASFSYMPRTEGEILQNANLKNFSLSELKSATRNFRPDSVVGEGGFGCVFKGWIDESSLAPSKPGTGIVIAVKRLNQEGFQGHREWLAEINYLGQLDHPNLVKLIGYCLEEEHRLLVYEFMTRGSLENHLFRRGTFYQPLSWNTRVRMALGAARGLAFLHNAQPQVIYRDFKASNILLDSNYNAKLSDFGLARDGPMGDNSHVSTRVMGTQGYAAPEYLATGHLSVKSDVYSFGVVLLELLSGRRAIDKNQPVGEHNLVDWARPYLTNKRRLLRVMDPRLQGQYSLTRALKIAVLALDCISIDAKSRPTMNEIVKTMEELHIQKEASKEQQNPQISIDNIINKSPQAVNYPRPSIM |
| AT2G26290 PBL12 | MAVFKKKKTSLTSLFLGCYKAKNASKYEGGEKAVMKIRTCPAFKRLSLSDISDPSSPMSVMDDLSHSFTSQKLRLFTLSELRVITHNFSRSNMLGEGGFGPVYKGFIDDKVKPGIEAQPVAVKALDLHGHQGHREWLAEILFLGQLSNKHLVKLIGFCCEEEQRVLVYEYMPRGSLENQLFRRNSLAMAWGIRMKIALGAAKGLAFLHEAEKPVIYRDFKTSNILLDSDYNAKLSDFGLAKDGPEGEHTHVTTRVMGTQGYAAPEYIMTGHLTTMNDVYSFGVVLLELITGKRSMDNTRTRREQSLVEWARPMLRDQRKLERIIDPRLANQHKTEAAQVAASLAYKCLSQHPKYRPTMCEVVKVLESIQEVDIRKHDGNNNKEGKKFVDINKFRHHRKGKRRVNIAYSDSLVYKESKAKQNDGI |
| AT5G35580 PBL13 | MVLCFQDPDNIYSPKKTKKDDGERVITKQKSFLGLSILDISNPSSTTLSEDLSISLAGSDLHVFTQAELRVITQSFSSSNFLGEGGFGPVHKGFIDDKLRPGLKAQPVAVKLLDLDGLQGHREFMTEVMCLGKLKHPNLVKLIGYCCEEAHRLLVYEFMPRGSLESQLFRRCSLPLPWTTRLNIAYEAAKGLQFLHEAEKPIIYRDFKASNILLDSDYTAKLSDFGLAKDGPQGDDTHVSTRVMGTQGYAAPEYIMTGHLTAKSDVYSFGVVLLELLTGRKSVDIARSSRKETLVEWARPMLNDARKLGRIMDPRLEDQYSETGARKAATLAYQCLRYRPKTRPDISTVVSVLQDIKDYKDDIPIGIFTYTVPTKPRREVKETSLQNFDKPRRETKVTSLQNFDKTRREVKDTSLQNFDKTRREVKETSLQNFDKTRREVKETSLQNFDKPRNVSTTDNHQKFRSPAHTARNHRITLRNGYNSPMRNEAGGERY |
| AT2G05940 AtRIPK | MAVKKKVSWRSLIVGCLGDPETLMASSKKPKRKNDVIKKQSSFQRLSILDMSNPSSNTLSEDLSISLAGSDLHVFTLAELKVITQSFSSTNFLGEGGFGPVHKGFIDDKLRPGLKAQPVAVKLLDLEGLQGHREWLTEVMFLGQLKHKNLVKLIGYCCEEEHRTLVYEFMPRGSLENQLFRRYSASLPWSTRMKIAHGAATGLQFLHEAENPVIYRDFKASNILLDSDYTAKLSDFGLAKDGPEGDDTHVSTRVMGTQGYAAPEYIMTGHLTARSDVYSFGVVLLELLTGRRSVDKKRSSREQNLVDWARPMLNDPRKLSRIMDPRLEGQYSETGARKAATLAYQCLSHRPKNRPCMSAVVSILNDLKDYNDIPMGTFTYTVPNTPDNKEDDGRVGNKPRKSSHHHHHQQQQSNHPRSSPSPTTKSPSPTAKSPRNSTENHRRTLRNGVNSPLRSEAGGERY |
| AT1G61590 PBL15 | MRDSSTTASTKSSPLWKPFASNCCSVDDQTVFGNLSRCRPSRSEFSKNHLGPLPSFRRLSFADLSRSSSARINEDLAQTLGADLVDFQMCELKMITQSFSGNYLLGEGGFGKVYKGYVDDYLRQSLKAQPVAVKLLDIEGLQGHREWLSEVIFLGQLKHPNLVKLIGYCCEEEERVLIYEFMPRGSLENHLFRRISLSLPWATRLKIAVAAAKGLAFLHDLESPIIYRDFKTSNILLDSDFTAKLSDFGLAKMGPEGSKSHVTTRVMGTYGYAAPEYVSTGHLTTKSDVYSYGVVLLELLTGRRATEKSRPKNQQNIIDWSKPYLTSSRRLRCVMDPRLAGQYSVKAAKDTALLALQCVSPNPKDRPKMLAVVEALESLIHYKDMAVSSGHWPLSPKSQGGKVSPKVRGDHRSGRKSAPGSLRS |
| AT5G56460 PBL16 | MGNCWCRFEPLNHRVSANAKSESPKEQSPTVEDKHIKEVQKLPSNPKEVEDLRRDSAANPLIAFTYEELKNITSNFRQDRVLGGGGFGSVYKGFIKEDLGDQEVPEPLPVAVKVHDGDNSFQGHREWLAEVIFLGQLSHPNLVKLIGYCCEDNHRVLIYEYMARGSVENNLFSRVLLPLSWAIRMKIAFGAAKGLAFLHEAKKPVIYRDFKTSNILLDMDYNAKLSDFGLAKDGPVGDKSHVSTRIMGTYGYAAPEYIMTGHLTPGSDVYSFGVVLLELLTGRKSLDKSRPTREQNLIDWALPLLKEKKKVLNIVDPKMNCEYPVKAVQKAAMLAYHCLNRNPKARPLMRDIVDSLEPLQATEEEALLVPPVQKAVITIIDEMPKNGLKKVEELKKVEEVKKVIEDAC |
| AT2G07180 PBL17 | MGICFSAEDQHYQFSQQQNYPKKTTPGKKAAVYLMKSDCEDVVGKVSGSGSGGGGLPLAPKNIKDLQSNPGYENVDIFTYEEMKIATKQFRPDYILGEGGFGVVYKGVIDESVRVGFKSTKVAIKELNPEGFQGDREWLAEVNYLGQLSHPNLVKLIGYCCEDDHRLLVYEYMAMGSLEKHLFRRVGCTLTWTKRMKIALDAAKGLAFLHGAERSIIYRDLKTANILLDEGYNAKLSDFGLAKDGPRGDQTHVSTRVMGTYGYAAPEYVMTGHLTSRSDVYGFGVLLLEMLLGKRAMDKSRACREHNLVEWARPLLNHNKKLLRIIDPRMDGQYGTKALMKVAGLAYQCLSQNPKGRPLMNHVVEVLETLKDDGDAQEEVMTNLHSRGKSVTLYEASSDSQGTRDGNGQRRRRPESGRSKSEAAVDTEKYVSTLSEPDTTKI |
| AT1G69790 PBL18 | MGNCLDSSARVGNRESTFGGSSRISRKPNQSSRLSSLTIPSYSNNSFTTSSWSNLTPRSEGELLPSPTLKAFTFNELKTATRNFKPNSMIGEGGFGCVYKGWIGERSLSPSKPGSGMVVAVKKLKSEGFQGHKEWLTEVHYLGRLHHMNLVKLIGYCLEGEKRLLVYEYMPKGSLENHLFRRGAEPIPWKTRMKVAFSAARGLSFLHEAKVIYRDFKASNILLDVDFNAKLSDFGLAKAGPTGDRTHVTTQVIGTQGYAAPEYIATGRLTSKSDVYSFGVVLLELLSGRPTLDKSKVGVERNLVDWAIPYLVDRRKVFRIMDTKLGGQYPHKGACAAANIALRCLNTEPKLRPDMADVLSTLQQLETSSKKMGSTQNIVMSPSSHMS |
| AT5G47070 PBL19 | MNCLFLFKSKKPRKQQKDNNKNKRKGKELLQNSAPELTNRSETSSFNLQTPRSLPSPRSIKDLYTEREQNLRVFSYEELSKATYVFSRKLVIGEGGFGIVYKGKILSNGDSSDPPLVVAIKKLNRQGLQGHKQWLAEVQFLGVVNHPNVVKLIGYCSEDGETGIERLLVYEYMSNRSLEDHLFPRRSHTLPWKKRLEIMLGAAEGLTYLHDLKVIYRDFKSSNVLLDDQFCPKLSDFGLAREGPDGDNTHVTTARVGTHGYAAPEYVQTGHLRLKSDVYSFGVVLYEIITGRRTIERNKPVAERRLLDWVKEYPADSQRFSMIVDPRLRNNYPAAGARSLAKLADLCLKKNDKERPTMEIVVERLKKIIEESDSEDYPMATTTTKESSQVRRRQVAKPEKQSLRGVSVRG |
| AT4G17660 PBL20 | MNCLFLFMSKKPKSRGNMEKEKKNIRGREFLQKSAPELTTRKTTLSFNLPTPRSLPSPTSIKDLYTDREQNQNQNLRVFSFKELSDATCEFSRKLKIGEGGFGSVYKATINNPTVGDSHSSPLTVAVKKLNRQSLQGHKQWLAEVHFLGVVNHPNVVRLLGYCSEDRERLLVYELMSNRSLEDHLFTLRTLTLSWKQRLEIMLGAAQGLAYLHEIQVIYRDFKSSNVLLNEEFHPKLSDFGLAREGPEGDNTHVTTARVGTDGYAAPEYVITGHLKTHCDVYSFGVVLYEIITGRRTLERMKPLAEQKLLEWVKKYPINSKRFKMIVDSKLCNKYPIAMVRRVAKLADHCVNKIDKERPTMAFVVESLTNIIEESNSEDMGSSVGIRG |
| AT1G20650 PBL21 | MTCCFSCLNPRTKDIRVDIDNARCNSRYQTDSSVHGSDTTGTESISGILVNGKVNSPIPGGGARSFTFKELAAATRNFREVNLLGEGGFGRVYKGRLDSGQVVAIKQLNPDGLQGNREFIVEVLMLSLLHHPNLVTLIGYCTSGDQRLLVYEYMPMGSLEDHLFDLESNQEPLSWNTRMKIAVGAARGIEYLHCTANPPVIYRDLKSANILLDKEFSPKLSDFGLAKLGPVGDRTHVSTRVMGTYGYCAPEYAMSGKLTVKSDIYCFGVVLLELITGRKAIDLGQKQGEQNLVTWSRPYLKDQKKFGHLVDPSLRGKYPRRCLNYAIAIIAMCLNEEAHYRPFIGDIVVALEYLAAQSRSHEARNVSSPSPEISRTPRRDL |
| AT1G76370 PBL22 | MRCFSCLNTQTNDMRINIDTLSDLTDYASVATKIDPRGTGSKSGILVNGKVNSPKPGGGARSFTFKELAAATKNFREGNIIGKGGFGSVYKGRLDSGQVVAIKQLNPDGHQGNQEFIVEVCMLSVFHHPNLVTLIGYCTSGAQRLLVYEYMPMGSLEDHLFDLEPDQTPLSWYTRMKIAVGAARGIEYLHCKISPSVIYRDLKSANILLDKEFSVKLSDFGLAKVGPVGNRTHVSTRVMGTYGYCAPEYAMSGRLTIKSDIYSFGVVLLELISGRKAIDLSKPNGEQYLVAWARPYLKDPKKFGLLVDPLLRGKFSKRCLNYAISITEMCLNDEANHRPKIGDVVVAFEYIASQSKSYEDRRTARKSTDSNRLRRETKQSY |
| AT3G20530 PBL23 | MKINCLFCCMSHRRFNRRSSSRQSIKDCIDAKNNITTFDNISFKTDSSRRRYISEEIAKLGKGNISAHIFTFRELCVATKNFNPDNQLGEGGFGRVYKGQIETPEQVVAVKQLDRNGYQGNREFLVEVMMLSLLHHQNLVNLVGYCADGDQRILVYEYMQNGSLEDHLLELARNKKKPLDWDTRMKVAAGAARGLEYLHETADPPVIYRDFKASNILLDEEFNPKLSDFGLAKVGPTGGETHVSTRVMGTYGYCAPEYALTGQLTVKSDVYSFGVVFLEMITGRRVIDTTKPTEEQNLVTWASPLFKDRRKFTLMADPLLEGKYPIKGLYQALAVAAMCLQEEAATRPMMSDVVTALEYLAVTKTEEDGQTVEGEEEEEEDERSKL |
| AT4G13190 PBL24 | MSCFLGPSTNNKSRENEGSSMAAPYEQQNLPRNDRRQITTWEAVGTNKESPKNIKAKSFKFRELATATNSFRQEFLIGEGGFGRVYKGKMEKTGQVVAVKQLDRNGLQGNREFLVEIFRLSLLHHPNLANLIGYCLDGDQRLLVHEFMPLGSLEDHLLDVVVGQQPLDWNSRIRIALGAAKGLEYLHEKANPPVIYRDFKSSNILLNVDFDAKLSDFGLAKLGSVGDTQNVSSRVVGTYGYCAPEYHKTGQLTVKSDVYSFGVVLLELITGKRVIDTTRPCHEQNLVTWAQPIFREPNRFPELADPLLQGEFPEKSLNQAVAIAAMCLQEEPIVRPLISDVVTALSFMSTETGSPSGLTGTALNPLSPKTVEDQGWLQCESPRDVYSLL |
| AT3G24790 PBL25 | MPAPYKQPNSPKRTTGEVVAKNANGPSNNMGARIFTFRELATATKNFRQECLIGEGGFGRVYKGKLENPAQVVAVKQLDRNGLQGQREFLVEVLMLSLLHHRNLVNLIGYCADGDQRLLVYEYMPLGSLEDHLLDLEPGQKPLDWNTRIKIALGAAKGIEYLHDEADPPVIYRDLKSSNILLDPEYVAKLSDFGLAKLGPVGDTLHVSSRVMGTYGYCAPEYQRTGYLTNKSDVYSFGVVLLELISGRRVIDTMRPSHEQNLVTWALPIFRDPTRYWQLADPLLRGDYPEKSLNQAIAVAAMCLHEEPTVRPLMSDVITALSFLGASSNSSNTGSNHLQQNRSNKYQDAVQWDSSPRYANSQM |
| AT3G07070 PBL26 | MNCFSCFYFHEKKKVPRDSDNSYRRNGEVTGRDNNKTHPENPKTVNEQNKNNDEDKEVTNNIAAQTFSFRELATATKNFRQECLIGEGGFGRVYKGKLEKTGMIVAVKQLDRNGLQGNKEFIVEVLMLSLLHHKHLVNLIGYCADGDQRLLVYEYMSRGSLEDHLLDLTPDQIPLDWDTRIRIALGAAMGLEYLHDKANPPVIYRDLKAANILLDGEFNAKLSDFGLAKLGPVGDKQHVSSRVMGTYGYCAPEYQRTGQLTTKSDVYSFGVVLLELITGRRVIDTTRPKDEQNLVTWAQPVFKEPSRFPELADPSLEGVFPEKALNQAVAVAAMCLQEEATVRPLMSDVVTALGFLGTAPDGSISVPHYDDPPQPSDETSVEDSVAAEERERAVAEAMEWGVASRAHSRNPSAS |
| AT5G18610 PBL27 | MSGCLPCFGSSAKDAASKDSVKKELSAKDGSVTQSHHISLDKSKSRRGPEQKKELTAPKEGPTAHIAAQTFTFRELAAATKNFRPECLLGEGGFGRVYKGRLETTGQIVAVKQLDRNGLQGNREFLVEVLMLSLLHHPNLVNLIGYCADGDQRLLVYEYMPLGSLEDHLHDLPPDKEPLDWSTRMTIAAGAAKGLEYLHDKANPPVIYRDLKSSNILLGDGYHPKLSDFGLAKLGPVGDKTHVSTRVMGTYGYCAPEYAMTGQLTLKSDVYSFGVVFLELITGRKAIDNARAPGEHNLVAWARPLFKDRRKFPKMADPSLQGRYPMRGLYQALAVAAMCLQEQAATRPLIGDVVTALTYLASQTFDPNAPSGQNSRSGSGPPFIRTRDDRRSLGDGSSLDSPAETRSRLGSPATHKNSPDYRRRDMVREVNAGSEGGSETGGGSGRKWGLSDLEGQESQRGSPASVGRSSRGTPRNRDLDRERAVAEAKVWGENWRERKRATNGPGSFDSTND |
| AT1G24030 PBL28 | MHFPLVSAWNKRRRSKSYDTDPCTFLFSIIFARWHKRVYRTAECWQIEDQASQPRKRRFGSSVYTLKEMEEATSSFSDENLLGKGGFGRVYQGTLKTGEVVAIKKMDLPTFKKADGEREFRVEVDILSRLDHPNLVSLIGYCADGKHRFLVYEYMQNGNLQDHLNGIKEAKISWPIRLRIALGAAKGLAYLHSSSSVGIPIVHRDFKSTNVLLDSNYNAKISDFGLAKLMPEGKDTCVTARVLGTFGYFDPEYTSTGKLTLQSDIYAFGVVLLELLTGRRAVDLTQGPNEQNLVLQVRNILNDRKKLRKVIDVELPRNSYSMEAITMFADLASRCIRIESKERPSVMDCVKELQLIIYTNSKGGLGGTIPTFRRL |
| AT1G74490 PBL29 | MGNCFESCSKRNDNEDDNLDSSVNSKPFSRANSDTGRSSNLSYPWSLKPLITRKCEAISALPPPHKEGDIMHSQYLKSFTLDELKNATGNFCPESLIGEGGFGFVHKGCINGGPGIELAVAVKKLKTEGLQGHKEWLREVNYLGRLHHPNLVKLIGYSLENEHRLLVYEHLPNGSLENHLFERSSSVLSWSLRMKVAIGAARGLCFLHEANDQVIYRDFKAANILLDSGFNAKLSDFGLAKEGPKDNRSHVTTEVMGTEGYAAPEYLATGHLTTKCDVYSFGVVLLEILSGRRVIDKSKSREEENLVDWATPYLRDKRKVFRIMDTKLVGQYPQKAAFMMSFLALQCIGDVKVRPSMLEVVSLLEKVPIPRHRKSRSKGFACTNSASMPSKRFLRHPKD |
| AT4G35600 PBL30 | MGACISFFSSSSPSKTGLHSHAATTNNHSNGTEFSSTTGATTNSSVGQQSQFSDISTGIISDSGKLLESPNLKVYNFLDLKTATKNFKPDSMLGQGGFGKVYRGWVDATTLAPSRVGSGMIVAIKRLNSESVQGFAEWRSEVNFLGMLSHRNLVKLLGYCREDKELLLVYEFMPKGSLESHLFRRNDPFPWDLRIKIVIGAARGLAFLHSLQREVIYRDFKASNILLDSNYDAKLSDFGLAKLGPADEKSHVTTRIMGTYGYAAPEYMATGHLYVKSDVFAFGVVLLEIMTGLTAHNTKRPRGQESLVDWLRPELSNKHRVKQIMDKGIKGQYTTKVATEMARITLSCIEPDPKNRPHMKEVVEVLEHIQGLNVVPNRSSTKQAVANSSRSSPHHYRYKAGALGAERKRATPGRFGSVEK |
| AT1G76360 PBL31 | MGNICPGSGSSTVNHEPVSTKPLPVRRVQTPENKKFPPPLVAMPALHPTFPLPETGHKLRPAATPPREKPQHRTTRSVENPPREKPQEKTRSVENPPREKPQEKTRSVETPPQEKTRPVDNPPSKPVEKLGLGRKAVPPSGKIVTPNLKMFTLVELKTATKNFRPESVIGEGGFGQVFKGWVDEKTLAPSRAGVGIPVAVKKSNPDSEQGLHEWQCEVRFLGKFHHPNLVKLLGYCWEENQFLLVYEYLPKGSLENHLFSKGAEALPWDTRLKIAIEAAQGLTFLHNSEKSVIYRDFKASNILLDSNFHAKLSDFGLAKNGPINGFSHVTTRVMGTQGYAAPEYMATGHLYVRSDVYGFGVVLLELLTGLRALDPNRPSAQQNLVEWAKPGLNQKKKVQKMMDPRLEQKYPLLAVTKTAELILRCLEADPKNRPPMDDVLRELEVVRTIRDQPQEERRKRSSGPDTNRVRGNDSPYGRRTGRTG |
| AT2G17220 PBL32 | MGLCWGSPSDSPPTTTPSSTGNISSVGTFKSSNNTTTTGTSRGSNISSNSGFSVASGEDAYPDGQILPIPNLRIFSLAELRASTRNFRSENVLGEGGFGKVFKGWLEDKTPGKQSNGTVIAVKKLNAESFQGFEEWQCEVNFLGRVSHPNLVKLLGYCLEGEELLLVYEYMQKGSLENHLFRKGSAVQPLSWEIRLKIAIGAAKGLAFLHASEKQVIYRDFKASNILLDGSYNAKISDFGLAKLGPSASQSHITTRVMGTHGYAAPEYVATGHLYVKSDVYGFGVVLAEILTGLHALDPTRPTGQHNLTEWIKPHLSERRKLRSIMDPRLEGKYPFKSAFRVAQLALKCLGPEPKNRPSMKEVVESLELIEAANEKPLERRTTRASPSIRQQQGHYRPQQLSSFRPRQNVSRAH |
| AT1G72540 PBL33 | MRFSWKNICLPISCINNTNQKKTTTTNPPKEKLLLLSRQTSVPSRVYMSDFSNSTISLNDFSNSFFINIHIFTYEELKTITQGFSKYNFLGEGGFGEVYKGFVDDSLKTGLKDQPVAVKALKREGGQGHREWLAEVIILGQLKHPHLVNLVGYCCEDDERLLVYEYMERGNLEDHLFQKYGGALPWLTRVKILLGAAKGLEFLHKQEKPVIYRDFKPSNILLSSDFSSKLSDFGLATDGSEEEDSNFTKSVMGTEGYAAPEYISAGNLTTMSDVFSFGVVLLEMLTARKAVEKYRAQRGRNLVEWARPMLKDPNKLERIIDPSLEGKYSVEGIRKAAALAYQCLSHNPKSRPTMTTVVKTLEPILDLKDIQNGPFVYIVPVAGVSEVHEIKCKDDVKVVKEETEKDAKVFPRHRAGRRNRRKHKAMRSRAVYSDTALYKSLGTSLYNPAN |
| AT5G15080 PBL34 | MGLDAVKAKGNWKSEKPKETENKNHKKKNGDDNKSRNEEEEEGEASGCWVKFRFMIGCIPSKSDLDASSSSIYGSNCTVTTMESKSANEKSNDQPVGQVSSTTTTSNAESSSSTPVISEELNISSHLRKFTFNDLKLSTRNFRPESLLGEGGFGCVFKGWIEENGTAPVKPGTGLTVAVKTLNPDGLQGHKEWLAEINFLGNLLHPNLVKLVGYCIEDDQRLLVYEFMPRGSLENHLFRRSLPLPWSIRMKIALGAAKGLSFLHEEALKPVIYRDFKTSNILLDADYNAKLSDFGLAKDAPDEGKTHVSTRVMGTYGYAAPEYVMTGHLTSKSDVYSFGVVLLEMLTGRRSMDKNRPNGEHNLVEWARPHLLDKRRFYRLLDPRLEGHFSIKGAQKVTQLAAQCLSRDPKIRPKMSDVVEALKPLPHLKDMASSSYYFQTMQAERLKNGSGRSQGFGSRNGQHQPVFRTLSSPHGSSPYRHQIPSPKPKGATT |
| AT3G01300 PBL35 | MGFDSVKVMENWQSKTSNENEKKKKKRRRKKNNNVRNSEHYEEEANGCWVKFRYIVCCASSTSDVETSLTLSTSTVGSQSAIVQSNDQPVGPVSSTTTTSNAESSLSTPIISEELNIYSHLKKFSFIDLKLATRNFRPESLLGEGGFGCVFKGWVEENGTAPVKPGTGLTVAVKTLNPDGLQGHKEWLAEINYLGNLLHPNLVKLVGYCIEDDQRLLVYEFMPRGSLENHLFRRSLPLPWSIRMKIALGAAKGLSFLHEEALKPVIYRDFKTSNILLDGEYNAKLSDFGLAKDAPDEGKTHVSTRVMGTYGYAAPEYVMTGHLTSKSDVYSFGVVLLEMLTGRRSMDKNRPNGEHNLVEWARPHLLDKRRFYRLLDPRLEGHFSVKGAQKVTQLAAQCLSRDSKIRPKMSEVVEVLKPLPHLKDMASASYYFQTMQAERLKAGSGSGSGRGFGSRNGQPVFRTLSSPHGQAGSSPYRHQIPSPKPKGATT |
| AT3G28690 PBL36 | MATKLESFKVVEKLGVEKGEKGKMMSKKKNVKKDGDESESGFWFRFKFIFSCISSRSKVDSSMNATAVIAEPKKVIEKLEGHPAPTKDTGCAESGSSTPLMSGELKYSSKLRIFMFNDLKLATRNFRPESLLGEGGFGCVFKGWIEENGTAPVKPGTGLTVAVKTLNPDGLQGHKEWLAEINFLGNLVHPSLVKLVGYCMEEDQRLLVYEFMPRGSLENHLFRRTLPLPWSVRMKIALGAAKGLAFLHEEAEKPVIYRDFKTSNILLDGEYNAKLSDFGLAKDAPDEKKSHVSTRVMGTYGYAAPEYVMTGHLTTKSDVYSFGVVLLEILTGRRSVDKSRPNGEQNLVEWVRPHLLDKKRFYRLLDPRLEGHYSIKGAQKATQVAAQCLNRDSKARPKMSEVVEALKPLPNLKDFASSSSSFQTMQPVAKNGVRTQGGGFVSRNGPPMRSLSSLNLPQASPYRYARQSPKPKGKEP |
| AT2G28940 PBL37 | MKCFHFTNGDKRTTTTIEEGVGGGDSVVSRASRLSWARSLSVASSTTSDPTRRSEFDSDWSFSPERLTFPKPLSQRWIGGLVPENDLKVFTFKELKIATKGFNRGLLIGEGGFGCVYRGVVDVSDSNGFDSKINVAVKQLNRQGLQGHKEWINEVNFLGVVNHPNLVKLVGYCADDDERGMQRLLVYELMCNKSLEDHLVGRVVSVSLPWMMRLKIAQDAAQGLAYLHEEMDFQLIFRDFKSSNILLDERFGAKLSDFGLARQGPPEGLGHVSTSVVGTVGYAAPEYVQTGKLTAKSDVWSFGVVLYELITGRRAVDRNRPRGEQKLLEWVKPYVSDSKKFHLIVDPRLEGQYYCMKSVQRVAALANKCLMKQPKSRPKMSEVVSLLGRIIDEEAENVPPPVADETEEIIKAELNGESEPELKKQGSSYRKKVLDLRDKMNLNKSLSKLDWRNWTPGLVRTW |
| AT2G39110 PBL38 | MNNSTSVKRLVSSKAMKCFYFSKDKTQDDEAKTRKFGSATMARGGSGSEFNSDTSTATSITSSLHVLSETHSNNLKVFVLDDLKTATKNFSRSLMIGEGGFGGVFRGVIQNPQDSRKKIDIAVKQLSRRGLQGHKEWVTEVNVLGVVEHPNLVKLIGYCAEDDERGIQRLLVYEYVQNRSVQDHLSNRFIVTPLPWSTRLKIAQDTARGLAYLHQGMEFQIIFRDFKSSNILLDENWNAKLSDFGLARMGPSDGITHVSTAVVGTIGYAAPEYIQTGHLTAKSDVWSYGIFLYELITGRRPFDRNRPRNEQNILEWIRPHLSDIKKFKMIIDPRLEGNYYLKSALKLAAVANRCLMVKAKARPTMSQVSEMLERIVETSSDGAPSGLPLMKSLTPKDAFEASRRERVKRRFVELLIGENGCPNLPTWSHKLVTSI |
| AT3G09830 PBL39 | MKCFLFSGGDKRGEQKTPISVSLTSIFSDREINRSGSEFNSRDVSGTSTESSMGRKNSYPPVSTRASNLREFSITDLKSATKNFSRSVMIGEGGFGCVFRGTVRNLEDSSVKIEVAVKQLGKRGLQGHKEWVTEVNFLGIVEHTNLVKLLGYCAEDDERGIQRLLVYEYMPNRSVEFHLSPRSLTVLTWDLRLRIAQDAARGLTYLHEEMEFQIIFRDFKSSNILLDEDWKAKLSDFGLARLGPSEGLTHVSTDVVGTMGYAAPEYIQTGRLTSKSDVWGYGVFLYELITGRRPVDRNRPKGEQKLLEWVRPYLSDTRKFKLILDPRLEGKYPIKSVQKLAVVANRCLVRNSKARPKMSEVLEMVNKIVEASSGNGSPQLVPLNSVKASRDARGKNNGGGGEGGWFGKLWNPKTIRAC |
| AT5G03320 PBL40 | MKCFLFPLGDKKDEQRSPKPVSPTSNFSDVNKSGSDFSPRDVSGTSTVSSTGRNSNTSMSARENNLREFTIGDLKSATRNFSRSGMIGEGGFGCVFWGTIKNLEDPSKKIEVAVKQLGKRGLQGHKEWVTEVNFLGVVEHSNLVKLLGHCAEDDERGIQRLLVYEYMPNQSVEFHLSPRSPTVLTWDLRLRIAQDAARGLTYLHEEMDFQIIFRDFKSSNILLDENWTAKLSDFGLARLGPSPGSSHVSTDVVGTMGYAAPEYIQTGRLTSKSDVWGYGVFIYELITGRRPLDRNKPKGEQKLLEWVRPYLSDTRRFRLIVDPRLEGKYMIKSVQKLAVVANLCLTRNAKARPKMSEVLEMVTKIVEASSPGNGGKKPQLVPLKSQETSRVEEGKNKKVLDGAEGGWLEKLWNPKNVRAC |
| AT1G61860 PBL41 | MMKLCPCFINPHQLGPNSPRDSFDEGLTAYRGHSRKLFALFTFRSHRKGSCRQKYITEEIKKYGNVKNCGRIFKFKELIAATDNFSMDCMIGEGGFGRVYKGFLTSLNQVVAVKRLDRNGLQGTREFFAEVMVLSLAQHPNLVNLIGYCVEDEQRVLVYEFMPNGSLEDHLFDLPEGSPSLDWFTRMRIVHGAAKGLEYLHDYADPPVIYRDFKASNILLQSDFNSKLSDFGLARLGPTEGKDHVSTRVMGTYGYCAPEYAMTGQLTAKSDVYSFGVVLLEIISGRRAIDGDRPTEEQNLISWAEPLLKDRRMFAQIVDPNLDGNYPVKGLHQALAIAAMCLQEEAETRPLMGDVVTALEFLAKPIEVVDNTNTTPASPTQTSSSDSSN |
| AT3G02810 PBL42 | MHCFPCFSSPKNKKSSTTNETNDNNEPKPDDRRRAEETEEIEQSEGTSLKIFTFRELATATKNFRQECLLGEGGFGRVYKGTLKSTGQVVAVKQLDKHGLHGNKEFQAEVLSLGQLDHPNLVKLIGYCADGDQRLLVYDYISGGSLQDHLHEPKADSDPMDWTTRMQIAYAAAQGLDYLHDKANPPVIYRDLKASNILLDDDFSPKLSDFGLHKLGPGTGDKMMALSSRVMGTYGYSAPEYTRGGNLTLKSDVYSFGVVLLELITGRRALDTTRPNDEQNLVSWAQPIFRDPKRYPDMADPVLENKFSERGLNQAVAIASMCVQEEASARPLISDVMVALSFLSMPTEDGIPTTVPILSFKDKSMSIALSRHDSNLVSPPPELATEDDKSSTSSGEESSLESEKESVSKNEYKKKHEEEDSSMESDDESDSNSEHEKDQPPKPIDEKNQAQSLKIKYRYSWEDIDVNDERLSSKSSQKSNDESTSSRYDSDRDQDEKGKEEEEEEEAEEKHTHIEHIDSSKTDDDQSVYFDNDDDSGDDNGGSLHRIKSDVAIDSIKE |
| AT5G16500 PBL43 | MIIMMNCFPCFTSQKSRNAPCTTNETNDDNVEHDEFRPPVVATTKRTEEREPAEQQPPVKTFNFRELATATKNFRQECLLGEGGFGRVYKGTLQSTGQLVAVKQLDKHGLHGNKEFLAEVLSLAKLEHPNLVKLIGYCADGDQRLLVFEYVSGGSLQDHLYEQKPGQKPMDWITRMKIAFGAAQGLDYLHDKVTPAVIYRDLKASNILLDAEFYPKLCDFGLHNLEPGTGDSLFLSSRVMDTYGYSAPEYTRGDDLTVKSDVYSFGVVLLELITGRRAIDTTKPNDEQNLVAWAQPIFKDPKRYPDMADPLLRKNFSERGLNQAVAITSMCLQEEPTARPLISDVMVALSFLSMSTEDGIPATVPMESFRDKSMSIALSRHGSCSVTPFCISRKDVGNKSSSSSDSEDEEEEKEQKAEKEEESTSKKRQEQEETATDSDDESDSNSEKDQEEEQSQLEKARESSSSSSDSGSERRSIDETNATAQSLKISYSNYSSEEEDNEKLSSKSSCKSNEESTFSRYDSGRDHDDSSRNTSMRINSLAHDDKEEDEEENHETRSYSDHDDSPRNTSMRINSLSHDDDEEEEEENHQTRLEHIHSSKSEDQSVYSDDDAGESGESSLHRIEAKEEEHISSDHD |
| AT3G26940 CDG1 | MVSCLCFRPSRKTKLKDKSHKRSIRNQTSSSSAQPAGTAKEVDSSSSQTVVQDSSRYRCQIFSYRELAIATNSFRNESLIGRGGFGTVYKGRLSTGQNIAVKMLDQSGIQGDKEFLVEVLMLSLLHHRNLVHLFGYCAEGDQRLVVYEYMPLGSVEDHLYDLSEGQEALDWKTRMKIALGAAKGLAFLHNEAQPPVIYRDLKTSNILLDHDYKPKLSDFGLAKFGPSDDMSHVSTRVMGTHGYCAPEYANTGKLTLKSDIYSFGVVLLELISGRKALMPSSECVGNQSRYLVHWARPLFLNGRIRQIVDPRLARKGGFSNILLYRGIEVAFLCLAEEANARPSISQVVECLKYIIDHTIRKERRTRRRLLGGNKDGAGTSRSPDETMMRMLEEEEEYVTSEEAIERRRVIVDDARTWAGMNRRGATPPTPTP |
| AT5G13160 PBS1 | MGCFSCFDSSDDEKLNPVDESNHGQKKQSQPTVSNNISGLPSGGEKLSSKTNGGSKRELLLPRDGLGQIAAHTFAFRELAAATMNFHPDTFLGEGGFGRVYKGRLDSTGQVVAVKQLDRNGLQGNREFLVEVLMLSLLHHPNLVNLIGYCADGDQRLLVYEFMPLGSLEDHLHDLPPDKEALDWNMRMKIAAGAAKGLEFLHDKANPPVIYRDFKSSNILLDEGFHPKLSDFGLAKLGPTGDKSHVSTRVMGTYGYCAPEYAMTGQLTVKSDVYSFGVVFLELITGRKAIDSEMPHGEQNLVAWARPLFNDRRKFIKLADPRLKGRFPTRALYQALAVASMCIQEQAATRPLIADVVTALSYLANQAYDPSKDDSRRNRDERGARLITRNDDGGGSGSKFDLEGSEKEDSPRETARILNRDINRERAVAEAKMWGESLREKRRQSEQGTSESNSTG |
| AT2G39660 BIK1 | MGSCFSSRVKADIFHNGKSSDLYGLSLSSRKSSSTVAAAQKTEGEILSSTPVKSFTFNELKLATRNFRPDSVIGEGGFGCVFKGWLDESTLTPTKPGTGLVIAVKKLNQEGFQGHREWLTEINYLGQLSHPNLVKLIGYCLEDEHRLLVYEFMQKGSLENHLFRRGAYFKPLPWFLRVNVALDAAKGLAFLHSDPVKVIYRDIKASNILLDADYNAKLSDFGLARDGPMGDLSYVSTRVMGTYGYAAPEYMSSGHLNARSDVYSFGVLLLEILSGKRALDHNRPAKEENLVDWARPYLTSKRKVLLIVDNRLDTQYLPEEAVRMASVAVQCLSFEPKSRPTMDQVVRALQQLQDNLGKPSQTNPVKDTKKLGFKTGTTKSSEKRFTQKPFGRHLV |
| AT3G21630 AtCERK1 | MKLKISLIAPILLLFSFFFAVESKCRTSCPLALASYYLENGTTLSVINQNLNSSIAPYDQINFDPILRYNSNIKDKDRIQMGSRVLVPFPCECQPGDFLGHNFSYSVRQEDTYERVAISNYANLTTMESLQARNPFPATNIPLSATLNVLVNCSCGDESVSKDFGLFVTYPLRPEDSLSSIARSSGVSADILQRYNPGVNFNSGNGIVYVPGRDPNGAFPPFKSSKQDGVGAGVIAGIVIGVIVALLLILFIVYYAYRKNKSKGDSFSSSIPLSTKADHASSTSLQSGGLGGAGVSPGIAAISVDKSVEFSLEELAKATDNFNLSFKIGQGGFGAVYYAELRGEKAAIKKMDMEASKQFLAELKVLTRVHHVNLVRLIGYCVEGSLFLVYEYVENGNLGQHLHGSGREPLPWTKRVQIALDSARGLEYIHEHTVPVYVHRDIKSANILIDQKFRAKVADFGLTKLTEVGGSATRGAMGTFGYMAPETVYGEVSAKVDVYAFGVVLYELISAKGAVVKMTEAVGEFRGLVGVFEESFKETDKEEALRKIIDPRLGDSYPFDSVYKMAELGKACTQENAQLRPSMRYIVVALSTLFSSTGNWDVGNFQNEDLVSLMSGR |
| Solyc07g041940 SlRIPK | MKIGWESLVPSCIKSHENSKKNPKMVKVSVTKQISFHGIPVSDLSSSTISSDLSISLAGSNIHAFTQQELRVITQNFSTSNFIGEGGFGPVHKGFIDDKLRPNAIKAQPVAVKNLDLDGSQGHREWLTEVIFLGQLRHPHLVKLIGYCCEEDNRLLVYEYMPRGSLENQLFRRYSVSLPWSTRMKIAIGAAKGLAFLHEAKKPVIYRDFKASNILLDSDYTAKLSDFGLAKDGPEGDDTHVSTRVMGTHGYAAPEYIMTGHLTAASDVYSFGVVLLELLTGRRSVDKGRPHREQNLVDWARPQLKDPRKLRRIMDPRLEGMYSEEGVQKAALVAYQCLSHRPKARPDMSNVVTTLEPLKDYEDNSMVTFVYTAPTDDQQVKQITSASPHHHHQKQQHHNHKRRSTPSSPTIHSETTIHKRLTPNSPLQNGFKRS |
| Solyc06g062920 | MATCGIDWKSVLPNCFKGNNVRSEAKVMENSKQMNSDHHRLAFSDISTDSRSVLISLDDLSSNAVIGSNLHVFTYEELKLITSDFSSANFLGKGGFGPVHKGFIDDKIKPGLDAQPVAVKLLDLDGNQGHQEWLTEVVFLGQLRHHHLVKLIGYCWEEEQRLLVYEYMARGNLEDQLFSRYSSCLPWLTRIKIMVGAAKGLAFLHGEEKPVIYRDFKASNILLDSDYRAKLSDFGLAKDGPEGDDTHVSTRVMGTHGYAAPEYIMTGHLTSKSDVYSFGVVLLELITGRRAMDKKRPLKERILVDWARPMLRDPHKLDRIMDPRLEGQYSTQGAKKVAALAYQCLSHHPRSRPTMSNIVKILEPVLDMKDIPMGPFVYVVPSSKPDKGTEIGELKTKVNDENKAGVRENEVDNAGENREDGNAKQRRVGHRYKHRLKTDASVYSDTHLYHKTVKHERTNKLNSY |
| Solyc12g049360 | MAIKILLKSLLPNCFNVKINDAIIHPHHDKINGNSRLGVGDISDPESPSNCMSEISSNAIIGTNLHVFTYAQLKVITSNFSSHNFLGEGGFGPVFKGFIDDKIKYGLDAQPVAVKLLDLEGDQGHIEWLTEVVLLGQLRHPNLVKLIGYCWEDKQRLLVYEYMARGNLENQLFRRCSSSLPWLTRMKIALDAAKGLAFLHGEKKPVIYRDFKASNILLDSDYTAKLSDFGLAKDGPEGSDTHVTTRVMGTYGYAAPEYMMTGYLTTRSDVYSYGVVLLELLTGRQAIDKKRGSREQNLVEWAKPFLRDSHKLERIIDPRLEGEYSTQGAKKVATLAYQCLSHQPKSRPTMSNVVKTLEPILDLKDIPIGSFVYVVPSFDSKSGLKTKGNEENKMHIISDKNHDKENAREMNQQKSATNI |
| Solyc08g061250 | MTGERLSWKSMIPCCYKVDDEFVKSKKQVKKQTSFQRLTLLDFDDPSSPLSADELSNSFIGSSLINFTFTDLREVTHNFSSANFLGEGGFGPVYKGFVDDKVRPGLKAQVVAVKVLDTDGLQGHKEWLTEIIFLGQLRHPHLVKLIGYCWEDDNRLLVYEFLPRGSLENQLFGKFSITLSWSIRMKIALGAAKGLAFLHEGDKPVIYRDFKASNILIDSDYTAKLSDFGLAKDGPEGDDTHVSTRIMGTHGYAAPEYIMTGHLTTMSDVYSFGVVLLELLTGKRSLDKSRREGEHNLVEWLRPYLRDPKRIARVMDRRLEDEYPMKGAQTAALVAYKCLNHYPKPRPTMDDVVKILETLQDENNNIDTSISDPMITMTLSSDFSSGSEQNEDTAAPERNRNNKYLNNERNQGYGWKHRLNRQRMVASYSDTALYRRH |
| Solyc10g074710 | MGNCGTREESAVVSNAHHQVQQQQGLSLGSRIGIDSKKHSHSRSTSDLSDPSTPRNLEDFRKNAVLYTHIIAFTLFELETITKSFRSDYILGEGGFGTVYKGYIDENVRVGLKSLPVAVKVLNKEGLQGHREWLTEVNFLGQLRHSNLVKLIGYCCEDDHRLLVYEFMFRGSLENHLFRRATVSLSWATRMMIALGAAKGLAFLHNAERPVIYRDFKTSNILLDSDYTAKLSDFGLAKAGPQGDETHVSTRVMGTYGYAAPEYVMTGHLTARSDVYSFGVVLLELLTGRKSVDKTRPSKEQNLVDWARPKLNDKRKMLQIIDPRLDNQYSVRAAQKACSLAYYCLSQNPKARPLMSDVVETLEPLQSSGGSTNEASSTGTAVRFAIGKVPDYRTHHRYGSSLGTAAGCRSPNPNCSPGGPAACRVR |
| Solyc08g074980 | MTKDSKSWRPFTANCCSADDQTIFGNFSRCKTSKSNFSKNNIGPLPSFRRLSFSDLSRSSSTRINEDLAQTFGPDLFDFQVSELRAITQNFSTNYLLGEGGFGTVHKGYVDENLRSSLKSQAVAVKLLNIEGLQGHREWLAEVIFLGQLRHKNLVRLIGYCCEDEERLLVYEFMPKGSLENHLFKRLSMSLPWGTRLKIAIGAAKGLAFLHGAEKPVIYRDFKTSNILLDSDFTAKLSDFGLAHMGPEGSNTHVTTRVMGTYGYAAPEYVNTGHLTTRSDIYSFGVVLLELLTGRRAMDKTRPKNEQNLVDWTRPYLSSSRRLRCIMDPRFGGQYSVKGAKEMAHLASLCTSLNPKDRPKMPAIIETLEAIQPLRDMAVACGQWPPSPKSSNKYVVYAPKGNKDSKIVVIKNPRMGVNSKSK |
| Solyc12g009310 | MGSCQSVEVGKTGQRPESKTVVHGVGSYPTKRNPVSQPKLKTPEGHDSRRRSSIVVIPETVEDLQQNPGISDLDIFKYEEMKMATKHFRPKQVLGEGGFGIVYKGVIDEHVRPGYKTTYVAIKELDPEGLQGDREWLAEMNYLGQLRHPNLVKLIGYCCEDHHRLLVYEYMESGSLEKHLFPRMCATLTWSRRMKVALDAAKGLAFLHGAEMPVIYRDFKTSNILLDADFNAKLSDFGLAKDGPMGDQTHVSTRVMGTYGYAAPEYVMTGHLTARSDVYAYGVVLLEMLIGRRAIDKTRPSQEYNLVEWARPLLNHNKKLFKILDPRLKGQYSSKTVAKVASLAYQCLSQNPKGRPVMSQVVEILEALQVPQGKD |
| Solyc01g028830 | MGNCCCRGQPSIYRVSSNAKSESPKDKSPSQNARMDHTKMPSNPEEVEDLRRSSATNPLIAFSFDELKIITCNFRQDYMLGGGGFGNVYKGYITEDLREGLQPITVAVKVHDGDNSYQGHREWLAEVIFLGQLSHPNLVKLIGYCCEAEHRVLIYEYMARGSVENNLFSRVLLPLPWSIRMKIAFGAAKGLAFLHEAEKPVIYRDFKTSNILLDLEYNTKLSDFGLAKDGPVGDKSHVSTRIMGTYGYAAPEYIMTGHLTPRSDVYSFGVVLLELLTGRRSLDKSKPAREQNLTDWAVPLLREKKKLLNIIDPRLDGDYPIKSVHKAAMLAYHCLNRNPKARPLMRDIVDSLEPLQIPGEVPTSEKPTLTVITDTPNGVIKEKVQT |
| Solyc01g010780 | MGLGGDGVKGGSWDVEKSKGRKKKEGDEEETGCWMKLRFISSCISSRSKVDNSISGISTRCESKSTNDTKIYQPVAPIISSTTTSNAESNSSTSKLEEELKVFSQLRKFTFNDLKLATRNFRPESLLGEGGFGCVFKGWIEENGTAPVKPGTGLTVAVKTLNHDGLQGHKEWLAEVNFLGDLIHPNLVKLIGYCIEDDQRLLVYEFMPRGSLENHLFRRREYLFIVTFSCYGTIYGSATTIIVGRVLVILAKDKWLHQRLTFYWSWSLPLPWSIRMKIALGAAKGLAFLHEEAERPVIYRDFKTSNILLDADYNAKLSDFGLAKDAPEGDKTHVSTRVMGTYGYAAPEYVMTGHLTSKSDVYSFGVVLLEMMTGRRSMDKNRPNGEHNLVEWARPHLGERRRFYKLIDPRLEGHFSIKGAQKAAQLAARCLSRDPKVRPMMSDVVEALKPLPNLKDMASSSYYFQTTQADRVGSSPSPNTRNVLRTQGSFSRNGQQPPPRSLSIPNGSHASPYRHQLSQNSPNPNGKP |
| Solyc05g007050 | MGLGGDGEKGESWDVEKSKGKKKKEVVEEETGCWTKLWFIGSCISSRSKVDSSISGISTHCDKYAFVLTSCIALAESKSTNDTSRDQPVAPIISSTTTSNAESNSSTSKLEEELKVSSRLRKFAFNDLKLATRNFRPESLLGEGGFGCVFKGWIEENGTAPVKPGTGLTVAVKTLNHDGLQGHKEWLAEVNFLGDLVHPNLVKLIGYCIEDDQRLLVYEFMPRGSLENHLFRRSMPLPWSIRMKIALGAAKGLAFLHEEAERPVIYRDFKTSNILLDADYNAKLSDFGLAKDGPEGDKTHVSTRVMGTYGYAAPEYVMTGHLTSKSDVYSFGVVLLEMITGRRSMDKNRPNGEHNLVEWARPHLGERRRFYRLVDPRLEGHFSIKGAQKAAQLAARCLSRDPKVRPMMSEVVEALKPLPNLKDMASSSYYFQTMQADRVGSSPSTKNGVRTQGSFSRNGQQHPRSLSIPNGSHASPYHQQFPQNSPKPNGKT |
| Solyc07g007980 | MEKEKKKEKNCGCWAVLRLSNVIGGSSDSKHSVNSIPRTSLVYDAATETRYLNASNREMCVPDEARVSSDTPTDPPTQLPPGAENKVQRQLLQFTFHELKSATGNFRPDSILGEGGFGYVFKGWIEENGTAPAKPGSGVTVAVKSLKPDGLQGHREWVAEVDFLGQLHHPNLVKLIGYCIEDDQRLLVYEFMTRGSLENHLFRRTIPLPWSNRLKIALGAAKGLAFLHGGSEPVIYRDFKTSNILLDSEYNAKLSDFGLAKAGPQGDKTHVSTRVVGTYGYAAPEYVMTGHLTAKSDVFSFGVVLLEILTGRRSMDKKRPSGEQNLVAWAKPYLADKRKFYQLVDPRLELNYSVKGVQKISQLAYICLSRDSKSRPSMDEIVKALTPLQDLNDLAILSNHSRLTQSGRRKKKLDGMQQLSFNHSRSIRGSPLHSGRQHCK |
| Solyc06g005500 | MGICLSARIKAESPFHTGLNSRSVSIDGGDSNSSSRVPSTTRSEGEILQSPNLKSFSFSDLRTATRNFRPDSVLGEGGFGSVFKGWIDENTFAATKPGTGVIIAVKRLNQEGFQGHREWLAEVNYLGQFSHPHLVKLIGYCLEDEHRLLVYEFMPRGSLENHLFRRGSYFQPLSWKLRLKVALGAAKGLAFLHSAETKVIYRDFKTSNILLDSNYTAKLSDFGLAKDGPTGDKSHVSTRVMGTYGYAAPEYMATGHLTSKSDVYSFGVVLLEMLSGRRAIDKNRPSGEHNLVEWAKPYLGNKRKVFRVLDTRLEGQYSMEVASKVANLALRCLSKDPRFRPSMSDIVKELEQLYQQSKDSGNTRSHASNRPRPRRRSAGDVANRNPSVAYPRPSASPLYAK |
| Solyc01g010660 | MGNCLGSSARVDATLSSHNISASGASRIPSRTSHSSVLSSLSIPSYSRKSSADTLSTPRSEGEILFSPNVKSFSFNELKSATRNFRPDSLLGEGGFGYVFKGWIDEHTLTAAKPGSGMVIAVKKLKPEGFQGHKEWVTEVNYLGQLRHPNLVKLIGYCIEGDDRLLVYEFMPKGSLENHLFRRGPQPLTWLTRIKVAIGAARGLAFLHDAKEQVIYRDFKASNILLDGEFNAKLSDFGLAKAGPTGDRTHVSTQVIGTQGYAAPEYVATGRLTSKSDVYSFGVVLLELLSGRRALDKMKVGVEQNLVDWAKPYLGDKRRLFRIMDTKLEGQYPQKGAYTAANLAWQCLSNEPKLRPRMSEVLAALEQLQAPKGVNKISQIEHRATSSSVPASPFKHRSSLSVTPSASPLKAYHKSPRGR |
| Solyc05g007140 | MGNCVGSPARVEATLSSTTPSAYEASRFPDKKSNSSVPSSLSIPSYGRKSSSESLPTPRSESEILYSPNVKSFSFNELKNATRNFRPDSLLGEGGFGCVFKGWIDAQTLTASKPGSGIVIAVKKLKPEGFQGHKEWLTEVNYLGQLRHPNLVKLIGYCIDGDNHLLVYEFMPKGSLENHLFRRGPQPLNWATRIKVAIGAARGLAFLHDAKEQVIYRDFKASNILLDAEFNSKLSDFGLAKAGPTGDRTHVSTQVMGTHGYAAPEYVATGRLTAKSDVYSFGVVLLELLSGRRAVDNTKVGIEQNLVDWAKPYLGDKRKLFRIMDTKLEGQYPQKGAYTAANLAWQCLSNEPKLRPKMSEVLTALEELQSPKGLSKLSHTYHRAIPSPVAVSPMRHHRSPLHMTPSASPLQAYQKSPRGR |
| Solyc04g011520 | MGSCLSVRIKAESPLHHGASDGRELSSRHSYSSAPLTPRSQSEILESSNLKSFSFNELRVATRNFRPDSVLGEGGFGCVFKGWIDENTFKAARPGTGLVIAVKRLNQEGFQGHKEWLAEITYLSQLSHPNLVKLIGYCLEDEHRLLVYEFMPRGSLENHLFRRSTYFQPLSWNLRMKVALEAAKGLTYLHSPEAKVIYRDFKSSNILLDANYNAKLSDFGLAKDGPIDGKSHVSTRVMGTFGYAAPEYMATGHITTRSDVYSFGVVLLEMLTGRRVMDKNRPHGEHNLIEWAKPFLTSKRKVLHIMDHRIEGQYSVEGALRAALLAVKCLALEPKFRPKMPEVVKALEQLQNLNESGSLKRETTQRKHHRTSTDEASGRKTTSYPRPAASPLVT |
| Solyc09g010850 | MGICLSNEIKAETTIFTGTGVGSRNVSGNGTENSNSNSKVSSGSVPPTPRSEGEILQSSNLRSFTFNELRASTRNFRPDSVLGEGGFGSVFKGWVDEQTLLASKPGAGIVIAVKKLNQEGLQGHREWLAEINYLGQLRHPNLVRLLGYCLEDDHRLLVYEFMSKGSMENHLFRSKRLSRGSYFEPLSWSLRMKVALGAARGLAFLHNAETSVIYRDFKTANILLDSNYNAKLSDFGLARDGPTGDKSHVSTRVMGTYGYAAPEYLSTGHLTAKSDVYSFGVVLLEILSGKKAIDKNRPTGEHNLVECSRAYLTSKRRVFRVLDSRLEGQYSLTRALKVASVALQCLAMDPKSRPTMDEVVTALEQLQESKDRVKNDKNKDQQLNRLSSQSSGELNKSFRSNSEETPRVASYPRPSASLRSI |
| Solyc05g053930 | MGICLSNQIKAETTFYTVSGKFALSLEISSDFFLLLDVLLILLLTVFCWHCKWIVLGLDSRNVSGNGTDISNSNSKHSPTTPRSEGEILQSSNLRSFTLSELRSATRNFHRDSVVVEGGFGSVFKGWVDEHTLAASMPGTGIVIAVKNLDQEGWQGHREWLAEINYLGQLHHPNLENLIGYCLEEDHRLLVYEYRPEGSMENHLFRRGSYYQPLSWSLRMKVALGAARGLAFLHNAEIKVIYRDFKTSNILLDSDYNAKLSDFGLARDGPVGDQSHVSTRVVGTYGYAAPEYLATGLSPCYPIPFSSSQSDGHLTAKSDVYSFGVVLLEILSGKKAIDKNRPMGEHNLVDWAKPYLTSKLRVSRVLDARLEGQYSLSHALKVAILSFQCISTEPKSRPTMDEVVTALEQLQQSKDVAKNDKKVRQVNQHSQSSFAFKKSCKSSTEETPAESNYPRPSLSFLS |
| Solyc02g087830 | MYSKSCKNHYQSQMDLISSFAVIQVSDETKETSGGPSAETARTTPDRGMVQEIASHPRRFSFNELRLATRNFRREDFLGMGGFGPVYKGWINENPVKPGTGLAVAVKILNRYGVQGHREWLAEVHFLQNLHHQNLVKLVGYCMEGHQRLIVYEFMARGSLENHLFRSVVLPWCTRIRIALGAAQGLAYLHEETQKPVIYRDFKASNILLDADYNAKLSDFGLARDGPEGDQTHVSTRVMGTFGYAAPEYLMTGHLTVKSDVYSFGVVLLEILSGRKAMDKNQRMGEHYLVSWTQPYLGNKHHFWRIIDPRLGGNFSKKGALKCTEIASLCLRNNPKLRPQMSEIVEMLMHLPSTSEFRDADDNSSSNNLEAKDKHVAGNLNSPTGPNASPASYTLSNKQKGKRPVRS |
| Solyc10g084770 | MGICLSNQIKAETTFYTVSGLDSRNVSGNGTDISNSNSKRSSASIPPTPRSEGEILQSSNLRSFTFNELRSATRNFRPDSVVGEGGFGSVFKGWVDEHTLAASKPGTGIVIAVKKLNQEGWQGHREWLAEINYLGQLHHPNLVNLIGYCLEEDHRLLVYEFMPKGSMENHLFRRGSYYQPLSWSLRMKVALGAARGLAFLHNAETKVIYRDFKTSNILLDSVCEYHLKGSLLQFTLSRRLETWDYNAKLSDFGLARDGPVGDQSHVSTRVMGTYGYAAPEYLSTGLSPCYPIPFSSSQSDGHLTAKSDVYSFGVVLLEILSGKKAIDKNRPMGEHSLVDWAKPYLTSKRRVFRVLDARLEGQYSLSHALKVAILSLQCISMDPKSRPTMDEVVTALEQLQQSKDVAKNDKKVRQVNQHSRSSFAFKKSCKSSTEETPAKSKYPRPSLSLLS |
| Solyc03g032150 | MGNCFGAKLSNSKSSSTYPSFSARPSTPDTSKSYSTGVGYSATSGSIGCSNFSAAASEDICLNGEILPIPNLKIYSFSDLKLSTKNFKSDSVLGIGGFGTVYKGWVDEKTLAPTKAGTGMIVAIKKLNSESTQGFEEWQSEVNFLGRLSHPNLVKLLGYCHEDKELLLVYEFMPKGSLENHLFRRSAAIEPLSWDLRLKIAIGAARGLAFLHTSEKKVIYRDFKASNILLDGNYNAKISDFGLAKLGPSGSNSHVTTRVMGTYGYAAPEYVETGHLYVKSDVYGFGVVVLELLTGLRALDTKRPNRQEKLVDWVKPMLSNKRKLKSIMDARMEGQYSSKAATLAAQITLKCLEVDPKNRPSMKEVMNVLEQIEAMKENPKESKSKSEHSSSHRHRQSPRSRQSPRQTNGQGFRSGSGK |
| Solyc12g077390 | MGNWITCCVRARDQVAGIPNAGTLTFPTSTEFSYYSGGTDWSHPLPVPNQNCTRKKRTGIVPSQRSESEILSSPYLRALLLSELTKATTNFHSDCLLGEGGFGYVYKGWLCKNTLTAAAPGSGLGVAVKKLKPRGLQGHKEWLSEVNYLGQLHHPNLVKLIGFCLEGENRLLVYEFMPRGSLENHLFPKSAPVLPWATRIKVAIAAARGLSFLHDAEPQVIYRDFKASNILLDSEFNAKLSDFGLAKSGPTGDHSHVSTQVMGTQGYTAPEYLATGKLTAKCDVYSFGVVLLELLTGRRVMDKRKAGAEQNLIEWATPYLHDKKKLFRIMDTKLEGQYPRKAATIAATLALHCVHPEAKGRPDMSFVLSALEQLPSKYMSGRQYGDQKKMSKSRHKSESTFPGSVRTKHASRVSEGTKSPPER |
| Solyc04g082500 | MGNCWPKPVDVVPPTSNTPPPVIMKKPINTSPGSNTRSVVAQPSGDGGDSRKMEVPASGKIITPNLKMFTLAELKSATRNFRPDTVLGEGGFGTVFKGWVDDKTFAPSRVGVGMPVAVKKSNADSEQGLKEWQAEVKFLGKLSHPNLVKLIGYCWEDKTFLLVYEHMQKGSLESHLFRKGAEALSWGTRLKIAIGAAKGLDFLHTIEKQVIYRDFKTANILLDSDYNAKLSDFGLAKMGPVNGDSHVTTKIVGTYGYAAPEYMATGHVYVKSDVYGFGVVLLEILSGRRVLDLNRPNGEHNLVDWAKPMLPDKKKLRKLMDPRLEAQYPSKAAFQIAEIILRCLEPDPKNRPSMEEILECLEQCNGIQKKPRAKNTTRNHNHRGPRSPLHVKKTGSGNVIGNQGYNAPKNRSY |
| Solyc00g007060 | MGNCFGSENLDPNPSNKSNSSTRPSTPDTSKNYSGGVGFSTSSSTGRSRFSAAASEDSCSNGEILPTPNLKTYSFSDLKAATRNFKSDTVLGVGGFGTVFKGWVDEKTLTPTKVGTGMVVAIKKLNSESMQGFEEWQAEVNFLGRLSHPNLVKLLGYCWEDKELLLVYEFMPKGSLENHLFRRSTAIEPLSWELRLKIAIGAARGLAFLHSSEKQIIYRDFKASNILLDGSYHAKLSDFGLAKVGPSAGNSHVTTRIMGTYGYAAPEYIATGHLYVKSDVYGFGVVLLELLTGLRALDTKRPGGQHNLVDWMKPMLSNKRKLKSIMDARMEGQYSSKAAILTAQLTIKCLEGEPKKRPSMKEVVEVLEQVELIKEKSKPSKSKSESSSHRYKQSPRSYPSPRGASNHGSGAGSGR |
| Solyc01g008870 | MGNCSGKSSKLAHASSSLIFDSNSGSSRSVKREQFHSHKQSFHNSFRANDSFVPSNLKSFTFNELKNATRNFRADSLLGEGGFGYVFKGWIDETTFAPCKPGSGMVVAVKKLKPESFQGHREWLAEVNYLGLLHHENLVRLIGYCAEFDNRLLVYEVMSKGSLENHLFKKGVQTIPWATRMCIAVDVARGLSFLHGLEANVIYRDLKASNILLDSVSSPKTEMDFNAKLSDFGLARDGPSGDRTHVSTRIVGTTGYAAPEYLASGHLTPKNDVYSFGVVLLELLSGKRATSAENAGGADEKLVEWAKPFLCDSRRVLRIMDTRLGGQYSKKGAQTAASLVLRCLNVDPRLRPTMDEVLATLELVQAPKDVMKSSQ |
| Solyc11g062400 | MGICFGKPVKLAHASSSLMSDSRTGPGSERKESSHSCRQTLQGSSGNALKSCKGDLSATCNLKSFTFNDLKNATRNFRADSLLGEGGFGYVFKGWLDENTLAPCKSGTGMVVAVKKLKSESCQGHREWLAEVNYLGQLHHENLVTLIGYCVESDNRLLVYEFMTKGSLENHLFRKGVQLMTWGKRMRIAVDVARGLSFLHSLDANVIYRDLKASNILLDSEFNAKLSDFGLARDGPDGDRTHVSTRVIGTRGYAAPEYIATGHLTPKNDVYSFGVVLLELLSGKRATGEENPGGAEETLVEWARPFLSDSRRVLRIMDTRLGGQYSKKGAQAAAALALRCLHVDPKLRPTMDEVLATLELLPTPKDNTKTSPPQANYNAPNDKICHPQKRYVTHSR |
| Solyc10g006340 | MKFGSKNKMKMKMKMNCFRCCVANEDHKKTLKKNNQEHKNNKSQSSFDNLSLKTDSNRRKYIAEEIAKLGKGNISAEIFSYLELKIATQNFNNDRLLGEGGFGRVYKGHIESKNLDVAVKQLDRNGFQGTREFLVEVLILSLLHHPNLVNLVGYCSDGDQRILVYEYMPNGSLEDHLLETSPDRKPLDWDTRMKIAEGAAKGLEYLHEKANPPVIYRDFKASNILLDENFDPKLSDFGLAKLGPTGDKSHVSTRVMGTYGYCAPEYASTGQLTTKSDVYSFGVVFLEMITGRRVIDTSKPSEEQDLVLWAQPLFRNKKKFHLMVDPLLEGNYPRKGLCQALAIAAMCLQNDASVRPLISDVVTALAFLAGNKKKDEDEEEAALDTPKTPLQSNTENIGTNEASVDT |
| Solyc09g075720 | MKCFQFSNDDHNKTRKSTSGQSSASVFTDSDLKGSEYSSQNVSTDISTVSFSCLSNKKACHLKVFTVEELKSATKNFSRSLMLGEGGFGGVYKGVLKDTNIAVKQLSQRGLQGHKEWVTEVNVLGVVEHQNLVKLIGYCAEDDERGIQRLLVYEFLPNRSVQDHLISRYMSPLPWETRLNIARDAARGLAYLHEGMEFQIIFRDFKSSNILLDEKWNAKLSDFGLARLGPSDGLSHVSTAVVGTVGYAAPEYIQTGRLTSKSDVWSYGVFLYELITGRRPLDRNKPKNEQKLLEWVRPHLSDLKKFEQILDPRLDGKYSIKSAQKLAAIANRCLVKHPRNRPKMSEVLEMVNQVVKATEAKIPQTPIDESSTPNVDDDMFMVRCLTASRRLVEHTSRENKLLVWKLWKPKLVSSN |
| Solyc01g112220 | MGVCFSSNSQSPSHFSSGISSQTTTTTSSSTVSNVSGNSQFSAASGVDEVYGQILPHPNLRTFSFTELKTATRNFRSDTVLGEGGFGKVYKGWLDDRASSRTASATGTVIAVKKLNSESLQGLEEWQCEVNFLGTLSHPNLVKLLGYCWEDKELLLVYEFMQKGSLENHLFGRGSAVQPLPWNIRLQIVIGAARGLAFLHASEKQVIYRDFKASNILLDGVRFFFFSPGTLSCLSQSYNAKISDFGLAKLGPSASQSHVTTRVMGTYGYAAPEYVQTGHLYVKSDVYGFGVVLVEMLTGLRALDTNRPSNQHNLVEWIKPHLSDRRKLKDKMDSRLEGRYPSRAAVQIAQLALSCLGPEPKTRPGMKEVVEKLEQIEAANERPKEPRITSRHQTAYRYGQPPLHHRSSLHPRNDVNRAYPLPKRAS |
| Solyc11g072660 | MGCFPCSGDPSMKKREKKRIQANNQYKRDDQPKPKSDTPKVSENKVVKKEEPENANQSTTRMDGNCSSAPRDGETDGIKINGVNRARAFKFNELVAATENFKAAYFLGEGGFGKVYKGFLADTGQVVAIKQLNPDGCQGNREFIVEVLTLSMADHPNLVKLIGYCVEGHHRVLVYEYMPLGSLEDHLHDPWSDKKRLDWNTRMMIAAGAARGLEYLHDKMKPPVIYRDLKGSNILLGEGYHPKLSDFGLAKVGPLGDKTHVSTRVMGTYGYCAPDYAMTGQLTFKSDIYSFGVVLLEIITGRKAIDNTRSAAEQNLVAWARPLFKDRKKFHQMADPTLEGHYPVRGLYQALAIAAMCVQEQPNMRPLIADIVTALNYLASQKYDPEAQPPQGPRKNSSSRKSRSIDDQKSPIKIEGHNRTRSIDGQKSPVNIEGHNRTRSIDDKKSPVIIEGHNRTRSIDYQKSPINVEGHNRRNSEGLN |
| Solyc04g082510 | MGCFSCMRFNRKDVRDFDEDMGSRSIKSSGKGIKGRSFGGKGGDSNNHKGNVARSFTFKELALATQNFREANLIGEGGFGSVYKGRLESGLVVAIKQLNLDGLQGHQEFIVEVLMLSLLHHKNLVNLTGYCTDGDQRLLIYEFMPMGSLENHLFDVEPGKKPLSWSTRLKIASGAAHGLEYLHCKANPPVIYRDLKSSNILLDNDFNPKLSDFGLAKLGPVGENTHVSTRVMGTYGYCAPEYAMSGKLTLKSDIYSLGVVLLELITGRKAYDNSKEAGEQNLVVWSRPFLKDRRKFVHMVDPLLNGQFSVRSLHHAVAITAMCIQEQASFRPIISDIVVALDYLVSQAESSDSQGGGSQTVKQTHDHKDN |
| Solyc01g067400 | MGGCFPCFGSSNKETGKDEVKKESFKDASSAAQSIHLTKVNSDKSKSRGSHDPKKDPAIAKDGPTAHIAAQTFTFRELAAATKNFRPESLLGEGGFGRVYKGRLESTGQVVAVKQLDRNGLQGNREFLVEVLMLSLLHHPNLVNLIGYCADGDQRLLVYEFMPLGSLEDHLHDLPPDKEPLDWNTRMKIAAGAAKGLEYLHDKANPPVIYRDLKSSNILLDEGYHPKLSDFGLAKLGPVGDKTHVSTRVMGTYGYCAPEYAMTGQLTLKSDVYSFGVVFLELITGRKAIDNTRSHGEHNLVAWARPLFKDRRKFPKMADPLLQGRYPMRGLYQALAVAAMCLQEQASTRPLIGDVVTALTYLASQTYDPNAVGAQSNRVGSSTPRSREDRLHSVDGVDSPEYTSAHHGSPSIQRNSPDSRKRDSARDFNTGIELRKIATSGGSGRKWGVDESERPDSQRNSPVSAGRTRETPRNRDLDRERAVAEAKVWGENWREKKKTNARGSSFDGIND |
| Solyc12g094680 | MSFHARGNSSDSGSGNGRKEGFFRSKKSGESNNQNNNVARSFAFKELAIATQSFRETNLIGEGGFGSIVAIKQLNLEGLQGNQEFVVEVLMLSLMHHNNLVNLIGYCTHGEQRLLVYEFMPLGSLENHLFDLEPGTTPLSWKTRLKIAAGAAHGLEYLHKANPPVIYRDLKSSNILLDNDFNPKLSDFGLAKLGPVGDNTHVSTRVMGTYGYCAPEYAMTGKLTLKSDIYSFGVVLLELITGRKAYDRTKKQGEQNLVVWSNPFLKDRRKYIHLVDPMLDGQFSSRCLHHAVAVTAMCLQEQASFRPSITDIVTALDYLLLQAQHSGTSRGGSQSDKHIPPPSTEEFNVSSRNRSYDNMAITF |
| Solyc05g024290 | MGCFSCFDSKEDEKLNPQKDRDDSDRKQPPPSNISRLSSGADRLKIRSSNGSKREFLGLKDAPDVQIAAHTFTFRELAAATNNFRPESFIGEGGFGRVYKGQLPSGQVVAVKQLDRNGLQGNREFLVEVLMLSLLHHPNLVNLIGYCADGDQRLLVYEFMPLGSLEDHLHDLPPDKEPLDWNTRMKIASGAAKGLEHLHDKANPPVIYRDFKSSNILLKENFFPKLSDFGLAKLGPTGDKSHVSTRVMGTYGYCAPEYAMTGQLTVKSDVYSFGVVFLELITGRKAIDSTKPQGEQNLVAWARPLFNDRRKFAKLADPSLQGQFPMRGLYQALAVASMCIQEQAAGRPLIGDVVTALSYLANQSYDPGTVPGQIHRFGADSVDRRNKDDRVGRILRSEDGEGGGSGRKWDVDGGSEKEDSPRETARMLNRDLDRERAVAEAKMWGENWRDKRRQNGQGSFDGGNE |
| Solyc01g088690 | MKCFFYFKERNRNRERKSAPVILQDQSKSDISGGAERVTKSSCSTSSARSFSDVYEGKGQNLRVFTFPELKQATNNFNRLLKIGEGGFGCVYKGNIKPADGKGESIIVAIKKLNRDGYQGHKQWVAEVQFLGVVDHPNLVKLIGYCAVDGERGIQRLLVYEFMSNRSLEDHLFNTAFPVLSWQRRLQMALGAAQGLAYLHEELEVQVIYRDFKSSNVLLDDDFKPKLSDFGLAREGPTGMHTHVSTAVVGTWGYAAPDYIETGHLTAKSDVWSFGVVLYEILTGRRSLERNRPKSEHKLLDWIKRYPADGKKFGMLMDPRLENQYSISAARKMAKLADTCLLKSAKDRPKMSQVVETLKQIIQISGENSSSTDTSFQGVDDVPVVEEKPKQMGATESAKRRMAHLAKLSEHVGGISRRRFLIMQKGKTT |
| Solyc08g077560 | MLKCFYIFKDKSRSRRRGESAPELSNESRRNGNSEGNRVTRSTGSVSSPRSIPEMYREREQNLRVFTLSELKEATRNFNRLLKIGEGGFGSVYKGSIQPSNGKGDPVVVAVKKLNTLGLQGHRQWIAEVQFLGVLEHPNLVKLLGYCATDGERGIQRLLVYEYMQNRSLEDHLFNKAVPVVPWRTRLKIILGAAQGMAYLHEGLEVQVIYRDFKSSNVLLDENFCARLSDFGLAREGPAGDRSHVSTAPVGTLGYAAPEYVETGHLSVKSDVWSFGVVLYEILSGRRTLERSRPVNEQKLLDWVKQFPADSRRFSMIIDSRLRNDFSIIAAKRIAKLADSCLNKNAKERPKMSEVVEILTQAVQESQGTTSTEATGAGPSRPTQLPLPNQLKKKTISETARPMIPLAQASLLKYGLYNLLEIS |
| Solyc09g008010 | MKCFPFYNAEKKEEPKTTKSNPVLSSSFGLFDSEFRHSHRESSSQNVSDTSSESRGRSQIPSLSDRPSDLRAFTFSELKAATKNFNRTTKIGEGGFGCVYKATVKTGEDSSKKIDVAIKQLGRRGLQGHKEWVTEVNVLGVAEHKNLVKLVGYCAEDDERGIQRLLVYEYMPNRSVENHLSARSETPLSWAMRLKIAQDAARGLAYLHEEMDVQIIFRDFKSSNILLDEQWNAKLSDFGLARLGPPEGLTHVSTAVVGTMGYAAPEYVQTGRLTSKSDVWSYGVFLYELITGRRPLDRNRPRSEQKLLEWVKPYISDSKKFQQIIDPRLDGKISRSAQKLSIVANRCLVRHAKTRPKMSEVLEMVNKVVETSTGIGNPGPPVRIAEPTSPESTRKGKRKVDTKLGDGSRLVRIWSTKLTNTC |
| Solyc06g005520 | MEKDEEEEGGTLSSRESKVSWVRSLSVASSSVDTRKSRSDLDSESRDFTETFEFYEFLTQRRANDLRVFSFSELKMATKGFSRGLMIGEGGFGCVYRGIVSAPCSDLKMEVAVKRLNRHGFQACFRLAMKIGHKEWINEVNFLGVVKHPNLVKLIGYCAEDDERGMQRLLVYELMRNKSLEDHLLARAAAPLSWTLRLKIAQDAARGLAYLHEEMDFQLIFRDFKPSNILLDEDFIAKLSDFGLARQGPTAGLTHVSTSVVGTVGYAAPEYVQTGRLTAKSDVWSFGVVLYELITGRRVLERNLPRAEQKLLEWVRPYVSDTKKFHFILDPRLEGHDCIKSAQRLASLANKCLAKNARSRPRMSEVVDMLENIIIDAEAVPETLKETENVNEEVDDTKEEGREEVELGKPESNSQKWGFDFKEMVSFRNKSIGKLDWRNWTAGLVKTS |
| Solyc10g085990 | MGWFPCSGPSKQTSKKKNSVDSGRHSTDKLKSKNSLRSKEVDKDAGSNQIAAKTFIFRDLAAATKNFRGDYLLGEGGFGRVYKGVIESNQVVAIKQLDRNGLQGNREFLVEVLMLSLLHHPNLVNLIGYCADGDQRLLVYEYMPLGSLEDHLHVGLNLELKYCSADPTPGKERLDWNTRMKIAAGAAKGLEYLHSASPPVIYRDLKCSNILLGEGYHAKLSDFGLAKLGPVGDNTHVSTRVMGTYGYCAPEYAMTGQLTLKSDVYSFGVVLLEIITGRKAIESSKTGGDFNLARPLFKDRRKFSQMADPMLQGHYPVRGMYQALAVAAMCVQEQPNMRPVIADVVTALTYLASQKFDHETRGGIQTSRPGPATSPRMKRW |
| Solyc06g075550 | MGCFPCSGDGDTSVKKPEKKIFQTISNHKYKRVEQPQPKQVVTPVASQFKVVKNEDAKVANRSMSKKDGGYSNISPTDGKTGGAKAQRFKFDQLIAATEDFKEDYFLGEGGFGKVYKGHLEDTGEIVAIKQLDPNGCQGVREFVVEVQTLSKADHANLVKLIGCCAEGDQRLLVYEYMSLGSLEDHLFDPWPNQKPLDWNVRMKIAAGAARGLEYLHDKMNPPIIYRDLKCSNILLGEGFHPKLSDFGLAKVGPSGDKTHVSTRVMGTYGYCAPDYAMTGQLTFKSDIYSFGVVLLEIITGRRAIDYTKSATEQNLVSWARPLFKDRKKFYKMADPALDGHYPIRSLYQALAIAAMCVQEQPNMRPPIVDIVTALSYLASLKYDPEIEPPIRKSYKSQSPQKSIKDDDETW |
| Solyc09g007170 | MAAMIRLVEPKAKSVQWVFLGCPGVGKGTYASRLSTLLGVPHIATGDLVRDELKSSGPLSKQLAEIVNQGKLVSDEIILNLLSKRLESGEAKGEAGFILDGFPRTVRQANCDKTSDFSLVSRGIGTGKESEDPLQQFATFLSMKRYPPPYVASGEILTEVTDIDLVVNLKLPERVLVEKCLGRRICSECGKNFNVASIDVAGENGAPRISMAPLNPPSQCISKLITRADDTEAIVKERLSIYWDKSQPVEDFYRSQGKLLEFDLPGGIPESWPKLLEVLNLDEQEHKLASSFNDSVKWACCVFVKFLSLWMIFGASVLHYCFSFTLFREILEELVLWLKLADIALKYGSDKLKAKTLLGSKEYVKDGESGQIGAKIFKFRELAVATRNFRGDCLLGEGGFGRVYKGRLDSNQIVAIKQLDRNGLQGNREFLVEVLMLSLLHHPNLVNLTGYCADGDQRLLVYEYMPLGSLDDHLHDLTPGKKVLDWNTRMKIAAGAAKGLEYLHDRASPPVIYRDLKCSNILLGEGYHPKLSDFGLAKLGPVGDNTHVSTRVMGTYGYCAPEYAMTGQLTLKSDVYSFGVVLLEIITGRKAIEFSKTGGEHNLVAWAKPLFRDRRKFSQMADPMLQGQYPERGLYQALAVAAMCVQEQPNMRPVIADVVTALSYLAAQKFDSDTEGVQNPRWTPATPPRTRRDADKRQKGGNHEPRW |
| Solyc01g008860 | MGICSGKISKLDHASSSLFFDSNSVSSSSVKKEQFHSHKHSFHSSFCSNDSFVPSNLKSFTYDELKNATKNFQADSLLDEGRFGYVFKGWIDETTFAPCKPGSGMVVAVKKLKAGRFQGHREWLVRRKELKNSNYLGLIVKAEVNYLGLLYHENLVNLIGYCAELDNRLLVYEVMSKGSLENHLFKKGDKVIPWATRMCIAVEVARALSFLHGLDASVVYRDLKASNILLDSDFSAKLSAFGLARDGPRGDRTRFPTRVCCTRGYAAPEYLASGNLTPQIDVYSFGVVLLELLSGRRATSAENAGGADEKLVEWAKPFLCDSRRVLRIMDIRLGGKYSKKGAQTAASLVLRCVDVDPRLRPTMDEVLATLELVKAPKVVHNRLNKADDKLVSREDKGARSISRGRNQDFH |
| Solyc06g083500 | MNCFSCFSFNENKTSNNKKKRKETSTSVHPHRENLSPEQAQPFSKTKTQHKPHSQPPQRTHPENIINQTVGNYKKDEENKNIAAQTFTFRELATATKNFRQECLIGEGGFGRVYKGHLDRTGQVIAVKQLDRNGLQGNREFLVEVLMLSLLHHNNLVNLIGYCADGEQRLLVYEYMQLGALEDHLFNVSGEGSPLDWFTRMKIASNAAKGLEYLHHKANPPVIYRDLKSSNILLDKEYNAKLSDFGLAKLGPMGDNSHVSSRVMGTYGYCAPEYQRTGQLSVKSDVYSFGVVLLELITGKRAVDPTKNGHEQILVAWAEPIFKDTSRYSELADPLFKEDVPKRSFNQAVAIAAMCLQEDPTVRPSISDVVTALTFLWAESGKGFGSPVSPVSTSLPVSSSDNEESHEEKKIIEREKAVAEAIEWGSNSRTQNERLP |
| Solyc09g061330 | MGCFSCFSSQEKKAFKRIHSKSKETTDVVRQRETLLNQQQQQRPHSLSRPKPQQDHRSHSQPLSVILYGYRLVFAFLIHKSSAENTRKVPAETPHQKSSNKGKGQNIAAQTFTFRELATATKNFRSECLIGEGGFGRVYKGHLNKTGQTVAVKQLDRNGLQGNREFLVEVLMLSLLHHSNLVNLIGYCADGDQRLLVYEYMSLGSLEDHLLDLERDKLPLDWFTRMKIALHAAKGLEYLHHRANPPVIYRDLKSSNILLDKEHNAKLSDFGLAKVGPTGENTHVSSRVMGTYGYCAPEYQQTGKLTIKSDVYSFGVVLLELITGRRAVDITRFGHQQNLVTWAEPIFKDTKRHIELADPLLGANFPRKSFSQAVAIASMCLQDDPVVRPLISDVVTALSTLSTPETELVSPFASPTRAPSPMHMSTEENIISTRECQDEVAEAIEWGSNSRKQNARSLASCGSSV |
| Solyc10g005300 | MRKTHLKSSRSSVYETRTTSKYDDSEGLAATMLRSATMHPGVSKQNMMAEDILGYGNVNISAEVFTFRELAHATDNFNPEFLVGEGGFGRVYKGHLKRTDQVVAVKQLDRNGVQGNREFLAEVLTLSLIKHPNLVNLIGYCADGNQRILVYEFMHNGSLEDHLLDFPSNKKPLDWYTRMKIAKGAAQGLEYLHDIANPPIIYRDFKASNILLDECLIPKLSDFGLAKLGPAEGEDHVSTRVMGTYGYCAPEYSMTEQLTSRSDVYSFGVVLLELISGRRVIDNTRPPEEQNLISWAKPLFKDKNMLTEMADPLLEGNYPVKELHQALAIANMCIQDEDYTRPLISDVVIALEYLAMPRDDEVTISKTEVEYSADELCLKDLTTETNCVS |
| Solyc07g064340 | MILGRLVKRDWLIWFPCCMTMDDNNQISSLEDSIIHQYKSANSQAQFANISLKTDSSRRRYIASEIEKFGKGNISAQAFTFRELCLATENFDSECLLGSGGFGKVYKGHIKSKNMAVAVKQLDRNGFQGTKEFLVEVLLLSLLRHSNLVNLIGYCSDGDQRILVYELMSNDSLEVHLLELGPDQKPLDWYTRMKIATGAARGLAYLHETANPPVIYRDFKSSNILLDENFDPKLSDFGLAKLGPTGDSSHVSTRVMGTYGYCAPDYACTGKLTIKSDVYSFGVVLLEIISGRRVIDSSRPSEEKNLIKWARPLISDKKLHLIADPLLRGNYPRRGLDKALTVATMCLQEDASTRPLMSEVVTALDYILNIKKHDDDHKEETADDTYKSPPALQTITSHVDRIASNKPNCVRERY |
| Solyc07g055180 | MEAENDEYHKKERAIAVTILVFASLAIASLFVAFSYYCYIRNKVAKRLKNRTYTESACEDKGNSFSNLEVIAEKGLQVFTFKQLHSATGGFGKSNVIGNGAFGSVYRGVLQDGRKVAIKLMDQAGKQGEEEFKVEVELLCRLRSPYLLSLIGYCSESSHKLLVYEFMANGGLQEHLYPIKGSNNFCPKLDWKTRLRIALEAAKGLEYLHEHVNPPVIHRDLKSSNILLDKNFHAKVSDFGLAKLGSDKAGGHVSTRVLGTQGYVAPEYALTGHLTTKSDVYSYGVVLLELLTGRVPVDMKRSPGEGVLVSWALPRLTDREKVVEIMDPALEGQYSMKEVVQVAAIAAMCVQPEADYRPLMADVVQSLVPLVKQPRPTVKPGSSSSFHATQSPSPHATQSPKA |
| Solyc12g005160 | MFCFSCCMSSSSDQDKINHTNSLAKSIHHQEQQKDTKSIASFANISIKTDSSRRKYIVEEIEKYGKGNISAQTITYNDLCLATNNFDSECLLGEGGFGKVYKGHIQSKNKDVAVKQLDRNGFQGNREFLVEVLLLSLLHHPNLVTLEAYCSDGDQRVLVYEFMSNGSLEDHLLEIGPEQKPLDWITRMKIAEGAAKGLEYLHETANPSVIYRDFKASNILLDEKFNPKLSDFGLAKLGPTGDKTHVSTRVMGTYGYCAPEYASTGQLTTKSDVYSFGVVFLEIITGRRVIDNSLPSEEQNLVVWATPLFRDKTKFHLMADPLMGEDYPMKALYQALAIAAMCLQEEAGTRPLMSDVVTALEFLSGNKKELDAEDEDEDEDEEGTCKSPPALQSFTSRLERADTNGIRERD |
| Solyc02g094380 | MGKPTTPHLPPDNAIKHSSLLEPTQQVLISKHDSPPRSNLPTFSYRDIATATNNFRRQSIIGEGGFGPVFKGKLNTNQVVAVKKLNHSGLQGDKEFFVEVHMLSLMRHPNLVNLIGYCSEGEQRLLIYEFMPLGSLEYHLHDITPDMKPLDWDTRMVIASGAAKGLEYLHNHADRPVIYRDLKSANILLGEGFHAKLSDFGLAKFGPIADNTHVSTRVMGTHGYCAPEYAGTGKLTMKSDIYSFGVLLLELITGCRAMDDSHEHGKEMLVDWARPMLKDRMNYVQLADPMLRGKFPQSVFRRVVELVLMCVQDDPHARPHMKDIVLALSYFASQKHDSPAAQIGSHGGEGTNGSSVDFDGAQMDITEIRASNKDQERERAVAEAKKWGETWREKGKQNADDDLDYKSRW |
| Solyc04g050970 | MSCLPCFQSKKTNEPPVQDKPVPVARPANDHPSSSPHFENNYKASCENGNNNNNRTDHGSSPVENGDSSNAKTFTFRELASATKNFRQECLIGEGGFGRVFKGTLQGGEVVAVKQLDRTGTQGNKDFQVEVLLLSLLNHQNLVNLIGYCADGDQRMLVYEYRPMGSLADHLIDIKEDQKPLDWQNRMKIASGAAEGLEYLHEKANPPIIYRDLRTTNILLDEDFTPRLSDYGLAKLAGGGNKSHISPRVMGTYGYSAPEYERSGELSFKSDVYSFGVVLLEIITGRRAVDTTRLTEEQNLVAWAQPIFRNPKRFREMADPLLKNKFPERSLNQAVGVAAMCLQEEPSVRPLISDVVAALTTLNVDEPIPESPQSPEKDNTDEYEQKSSDNEALSNKNEDESSEDDQDNVHYNKKIDINKNVFDSDEDDGASSDYGYGSTSGSSENEKEDISLEPGGGIPTKFVKWSSESKRKSKIKSSSRAIRSTSRRKSKVKRSESIISNDDTEKDTFNLKDNNNHRQQKNAKSKTVSFSGFSSQSSDDAESDGENDIGSNQSRHVQFRS |
| Solyc07g042590 | MACAVEVCATLSWSRRMRIALDAAKGLAFLHGAERPIIYRDFKTSNILLNAEFNAKLSDFGLAKDGPMGDQTHVSTRVMGTYGYAAPEYVMTGHLTARSDVYGFGVVLLEMLIGRRAMDKSKPSREHNLVEFARPLLNHGKKLFRIIDPRLDGQYSSKTALKVANLAYQCLSQNPKGRPVMSQVVEILEGLQPQDKGEDAILMTGGGSVTLYEAKPNPLKCESGREADASKSNQTNGRSKSELPKECDLYSPSPDLVLDVGSVSSRS |
| Solyc09g064270 | MPTLMLLFLLSLLTLLTTTTLGITLPHIYLSPTLPPNKPYFASKLFLGDARLVSTHVSFAPISAPHRHHFKPHMIPSRAPAPSPASQGLAAAPIASRVVGHHRHRHNRPRARVSPSPAVGSGCGEVCAEPFASVPFVTPCTCVFPMKVRLLLDKSLYSIFPVVRDLGIEVAKGTYLRPSQVVVVGASADNQNQERTIVDINLVPLEDKFDNTTAMLIYERFWKKKMPLNRTMFGDYDVMHIMYPGLPSSPPSGIGSGNGPTGSAIDQQFPITADFGNKSQKMNPRVIFLIASSALVLLVVCCGALVVLLNCRRTSRPSNAVGPVFTSSMHKRSGKGIGSTISSSTASSTSVSLISAMPASILSVKTFTLAELEKATDKFSLKRVLGEGGFGRVYHGILEDRTEVAVKVLTRDNNQNGDREFIAEVEMLSRLHHRNLVKLIGICSEERTRSLIYEIVRNGSVESHLHGKDRIKGPLDWDVRLKIALGAARGLAYLHEDSNPRVIHRDFKASNVLLEEDFTPKVSDFGLAREATEGSDHVSTRVMGTFGYVAPEYAMTGHLLVKSDVYSYGVVLLELLSGRKPVDMSQPPGQENLVTWARPLLTTREGLEQLVDPSLAGTYDFDDMAKVAAIASMCVHPEVTQRPFMGEVVQALKLIYNDNDETCADGCSQKESSLPDSDFKGVPSDSSWWNAGGLTPRLTYGQASNFMTMDYSSGPLEEFENRPFSASSFNLGGEAGLLSHGNRSGPLRTVRSKPALYRLRGSMSEHGALLPRHAWKDGTNYDASF |
| Solyc01g079340 | MLDLSKLLFFPCCARLSKRGQSLKLKNLTILFFFLCITNAELPDEPFEQQPVTPVNKPVEAPGLPDLPLPANVPGFHRQHRKHSPHGAPWLGLAPAQPPDYGPLVTAAHAPSSSSLSKPSMKKNGLVPPSAGLAPPQSSPSTLPTGLVQPPLSPHTSTDCCGQDMVLKRGSLDCECVYPLKIDLLLLNVSSNPNWKLFLNEFASQLGLKVSQIELINFYMVDLSKLNISMDITPYKGVSLSSDEASAVNASLSMHKIQLDPTLVGGYQLLNITLFKPPVSSQAPRSAMSPVLAAPHLPSAPAVTVSSHKGRHPSLILIVGIVAGILIITIISTLFICFCGSNHGQKKGSHKEAEKPMRVETVPAQGSFPHPTSTRFLPYEELKEATNNFAPASILGEGGFGRVYKGVLSDGTAVAIKRLTSGGQQGGKEFLVEVEMLSRLHHRNLVKLVGYYSSRESSQNLLCYELVSNGSLEAWLHGHLGLNCPLDWDTRMKIALDAARGLAYLHEDSQPCVIHRDFKASNILLENNFHAKVADFGLAKQAPEGQANYLSTRVMGTFGYVAPEYAMTGHLLVKSDVYSYGVVLLELLTGRRPVDMAQPSGQENLVTWARPILRDKDRLEELADPRLEGKYPKEDFVRVCTIAAACVAPEASQRPTMGEVVQSLKMVQRVTEYQDTTTINSGARPNLRQSSTTFESDGTSSMFSSGPYSGLSVFENDVSRAAVFSEDLHEGR |
| Solyc05g055860 | MSCLPCFKKKTESSEDDVPVAQSKGISTTPPNPGITYVHFLHAFWIKRPAEPFHKGDNAGGNVNSNAKTYTFRELASATKNFKQECLIGEGGFGRVFKGTIQGGEIVAVKQLDRSGTQGNQEFIVEVSKLTLLKHQNLVNLLGYCADGDQRILVYEYMPMGCLNDHLLDLEEDKKPLGWLSRMKIALGAANGLEYLHETTNPPIIYRDLKGTNILLDKDFNPRLSDYGLSKLAGGGSRTNMSPMMMGTGYCAPEFEKNGEHTLKSDVYCFGIVLLELITGRRAVDTTRPADEQNLVAWAQPYFKDPKKFHELADPRLGKSFPVKGLNQAVGVTAMCLQDEPMVRPLIGDVVAALTFLTMPQPDDPIPSSPPAPTPTSNNEDQASSENEDQDYSDDEDQGYSDEEYEDSENDEQNDEKVHDKQRSQKVSDKRKSQKNRDSQEDEKASSEYGYGSASGSSEYEDSGDGEKPKITAKSAKYASHSRHKSKVKSRTKSTNSGTSNR |
| Solyc05g025820 | MKITWESLVPRCCISEKPKTSKLVSKQSTFHRISASDFSNSTISEDLSISLAGSNLHVFSVQELKVITQNFSSSNFLGEGGFGPVHKGFIDDKLRPGLKPQPVAVKFLDLDGTQGHREWLTEVIFLGQLRHPHLVKLIGYCCEEEHRLLVYEYMPRGSLENQLFRRYSVSLPWSTRMKIALGAAKGLAFLHEAEKPVIYRDFKASNILLDSDYNAKLSDFGLAKDGPQGDDTHVSTRVMGTQGYAAPEYLMTGHLTAASDIYSFGVVLLELLTGRRSVDKTRPNREQNLADWARPQLKDARKLARVIDPRLEGLYSSEGVQKAAYVAYQCLSHRPKARPDMTTVVKTLETLKDYKDISTMTFVYIAPVLDQHKRSPQRELLNKTNTNTKSLHKKLTPNSPLHNDFHRA |
| Solyc03g121610 | MGLLQSFMLLRFLVILSVLSIQLSAGFNLHSLKTAASAFLKDGRIDLSVQRNAGSRKSLLQEDLFPTPTSRSKRHAMFAAAPHLEAFHSAPHPYHHPTKATYQHKILEPSNYADAPLTSTHFRNSGGKQVHSSASAPIISSIRHHHRRNKHSDSTAKPNDRLHPPSSRWSGPSISPFMSPVPSSISWAPVSSPITQPSHTEIPMSTPTISPTSSSIKRKKLRPPPLPVMTLPPPPPNHDCSSLTCTEPLTYTPPGSPCGCVWPIQVAMCLNVTLYTFFPLVSELAKEIAAGVLLNTSQVRIMGANAADQQLEKTIVHINLVPTDGKFDGTTALTIYQKFWKRAVFIKTTDFGAYEMVYVRYPGLPPSPPSRHSSSATIDDLPAYPGNENNGMTIKPLGVDVSSRMRKKGIPRNMIIVIVISSITAFVVCMGLIWLLLFKRGCYAQSPEQPPHILVSSQGKTSGDAGSMILASKPSSKSMSFSSSILAYTGTAKIFSTNDIERATNNFDISRVLGEGGFGLVYSGTLDDGRKVAVKVLKRDDRQGGREFLAEVEMLSRLHHRNLVKLIGICTEENCRCLVYELVPNGSVESHLHGIDKEASPLDWYARMKIALGAARGLAYLHEDSSPRVIHRDFKSSNILLEHDFTPKVSDFGLARTALEEGNRHISTHVMGTFGYLAPEYAMTGHLLVKSDVYSYGVVLLELLSGRKPVDLSQPQGQENLVAWARPLLTTKEGLEIIIDKAMESDIPIDSISKVAAIASMCVQPEVSHRPFMGEVVQALKLVCDEFDDTRGPMSRSCSQEDLSMTDTSLVHKSIPGFDSPLNVQMELSASELKSASARYGTVESESFRRQFNSAPLKMGRKRNFWQRLRVLSSGSMSEHSFSSKT |
| Solyc05g005877 | MPFGLVSAWNKRRRSKSEDMLNPWIYKPVEYWQIEDQNPPAAKRRHGSSVFTLREMEEATNSFSDDNLLGKGGFGRVYKGTLRSGEIVAIKKMDLPSFKEAEGEREFRVEVDILSRLDHPNLVSLIGYCADGKHRFLVYEYMHKGNLQDHLNGIAEVKMDWPLRLKVALGAARGLAYLHSSSAVGIPIIHRDFKSTNILLNTNYDAKISDFGLAKLMPEGQQSCVTSRVLGTFGYFDPEYTLTGKLTLQSDVYAFGVVMLELLTGRRAVDLTLGPSDQNLVLQVRHILNDKKKLRKVIDPEISRNSYTMESITMFANLASRCVRTDSSERPLMVDCIKELQLILHTNAKGLNMTMHTFRMI |
| Solyc01g098740 | MEDNYSQNHHHSESQSHKNHQHNDILPSTSVLLIIVPIIIIILLIAISLLIVMLKRIQSAKHNGTNNSSKSVINKNNCMFVAHSTIDIHLSPDVKGGCLPGHGGNSGRMPETKLRGVQVFTYKQLEMSTDKFSEANVIGNGGYGVVYRGVLIDGTVAAIKVLQREGKQWERSFRLEVDLLSRLHSPYLVELLGYCADQHHRLLIFDYMPNGSLQQHLHNAHKQSTNSLNWGIRLRIALDCARALEYLHEHTTPSVIHRDFKCSNVLLDQNFRAKVSDFGLAKIGSDKLNGLISTRVLGTTGYLAPEYASTGKLTTKSDVYSYGVVLLELLTGRVPIDTKRPPGEHVLVSWALPRLTNREKVVEMVDPTLQGQYTKKDLIQVAAIAAMCVQTEADYRPLMTDVVQSLVPLVQTYSSSCSANSFRSYNHTASPRS |
| Solyc12g007110 | MSTPTPVTALAPSNATNSPPPSTPATTPPPASPTPPATAPPPASPTPPATAPPPSTPAQAPPPARTPTPPAATPPPDASASPPPPTTPAPSSNPPPSTTPAPSGSPSPPSTTPSTPSPPARGNSPPSPSGGSPSPPSGGRPSPPAVSSPPSDEGSSGISTGVVVGIAIGGVLILAILSLLFIFCKKKKRRNHEPVNYYVPPPPPPLGVKADPHGGQMHHWQQNAPPSADHFVAMPPKPSPPPVGGLLPSHAPPRAPSPQPQPYMNSSGASSNYSGSEIALPPPSPPMSLGFSQSTFTYEELVRATDGFSDANLLGQGGFGYVHKGILPNGKEVAVKQLKAGSGQGEREFQAEVEIISRVHHKHLVSLVGYCITGAQRLLVYEFVPNNTLEFHLHGKGRPPLDWPIRLKIALGSAKGLAYLHEDCQPKIIHRDIKAANILVDFNFEAKVADFGLAKLTSDVNTHVSTRVMGTFGYLAPEYASSGKLTEKSDVFSFGVMLLELITGRRPVDSTQSYIEDSLVDWARPLLTRALEDEKFDGLVDRRLENDYNHNEMARMVACCAACVRHSAKRRPRMTQVLRALEGDVSLSDLNEGIKPGHSTVYSSYTSSDYDTLQYNEDMKKFRKMALATSQEYASSDQYSNPTSEYGLNPSGSSSEGHQTAEMETGRMRKDSRGFSGSKGFSGTS |
| Solyc05g010140 | MSVVVSPPPSLAPIPVPPIVLSPPPQLSSPPPASQPNATAPPVSSLPPTLPPQSSPPPALPTLPQSPTPSNVTSPPPSNVTSPPPMASPPTESAPPTVSPPPIPPASSPPPVSSPPPSSSPPPQSSPPPASAPPPTSSPPVSSSPPPEVEPPPVSPPPQPTVPTSSPPPPPKDDPPPASPPPQPTTPPSSPSPPPKVDSPPFSPPPPAQNPDPTAPPQSPEPPKGSPPVNSPPSPASVPPRGSPPTPASDPPANTPPSPAFTPPQGSPPTPSLEPPKNTPPSPSVPSGGTTTDRPSDNAAGSANSSSSSGIGTGGTVAIGVIVAVLLLGIVGLVGWCLWKRKKKAFRPSGGNVMPTPSGSTPNSDSVLLKIQESTPDTRNGTGNKFLNSPGGSGGFGNPKIWFTYEELVKATGDFSAENLLGAGGFGSVYKGCLPDGRDVAVKQLDIGGRQGDREFRAEVEIISRVHHRHLVSLVGYCISENRRLLVYEYVPNNTLYFHLHAEGRPVMDWTTRVKIAVGAARGIAYLHEDCNPRIIHRDIKSSNILLDINFEARVSDFGLAKLAQDAKTHVTTRVVGTFGYMAPEYASTGKLTEKSDIYSFGVVLLELITGRKPVDTSQPLGDESLVEWARPLLSHALEKVEFDQLVDPRLERNYVIPEMFQLIEAAAACVRHSAAKRPGMGQIMRAFDNMSASDLTNGMKVGESTIYNSADQSAEIRLFRRMAFGSPDFSSDFFSQGTQHSGESAEDRV |
| Solyc03g120760 | MSTGLPGVPLVPPLPFPPADNATTPTSPVNPSTTPPSPDVASQPSPATLLNPPPSSINGSSPTSAPSNGSSNSTTLVALGVGIGIGGALVLICVCIFALWYKRRKRRLGLNGFGHDPSQAPKDNLFGGQHHHWQQNAPPSTENMVRIPINPSPPPSITSVPQVTAADGCTPSLPPSTGSNLSTDKPFLQTTPDSSSSFSRTTFTYQELAFATDYFSVSNLLGQGGFGYVYKGVFRDGKEVAIKQLKAGSGQGEREFQAEVEIISHVHHKHLVSLVGHCISGVQRLLVYEFVPNKTLEFHLHGDITAGKEHPPLSWETRMRVTLCSARGLAYLHEECHPKIIHRDIKASNILLDDNFDAKVADFGLARLNYDSDTHVSTRVMGTFGYLAPEYALTGKLTEKSDVFSFGVMLLEIITGRRPIDKAQPYLDDNIVDWARPLLTQALDDGNFDTLADPRLDKNYDLTEMTRMVTCAAVCVRHLARRRPRMSQIVRALEGNLPLDELNGLRPGHSGIHESYGSSDFDAVQYKEDLKKFRKMALESQAHNSSECSGPTSEFGPHPSGSSSEGLRTTQGTRSSVEGHREIRID |
| Solyc02g062790 | MDSSPGTASSNDTDSKGTPSSNGTPPSSSSSDSKNDSPSSSSSSPSPKNESPPSSSPPPQSPPPSSNASPPPKNDSPPPSSPPPSSPGSDASPSKDNNNDNNNNNDNNNNNNNNNNTNNQSPNPSSNTPSQSPPPSSTSNNPSTPNSPKNPPSFLSFSPPPPVLTPAPPHAHLSPPPRSGSNEPSPANNDSSSTVGIVAGIAVGGIVIVALIILCVWCSRRKKKQRYYMAPGPKGGDPYYNNNNQWNRRQSMDHVMKVPQSEMGTQSGWAGATPHQQGANSSGEFGSGYSGQAAPLPSQSPNMGLGGFSQSQFTYEELAKATDGFSQANLLGQGGFGYVHKGVLNDGRVVAIKSLKAGSGQGEREFQAEVEIISRVHHRHLVSLVGYCIANGQRMLVYEYVDNKTLEFHLHGKGQPVMDWETRLKIALGSAKGLAYLHEDCQHRIIHRDIKAANILLDLNYEALVADFGLAKLTSDNNTHVSTRVMGTFGYLAPEYASSGKLTEKSDVFSYGVMLLELITAKKPVDPSNMMEDSLVDWARPLLTRALEEEKYDGLVDARLEGNYDTDELHRMIGCAASSIRHSAKRRPKMSQIVRALEGNSSLEDLNDNPKPSKVASFAGAPTQSYDTGMYNADMMKFRKMIMPTQEFSSSPSE |
| Solyc01g101100 | MAGSLNDELSQKTAVFGLKVWQLIGIFVGIFIVVILLLLTLYLTLRRKSRRGTDNLPVSQIPSVSKEIKEVRVEQVSTNDFSPRDGILLTIHDKTSDKESDKVLVHLGMGKKNGDNSSQSGSFHHVDKEGCGSQSGEEGSSCKSAMYKAYNSHPITAPSPLTGLPEFSHLGWGHWFTLRDLELATGRFSKENILGEGGYGIVYRGNLINGTPVAIKKLLNNLGQAEKEFQVEVEAIGHVRHKNLVRLLGYCIEGTHRMLVYEYVNNGNLEQWLHGAMRHHGYLTWEARMKVLLGTAKALAYLHENIEPKVVHRDIKSSNILIDDDFNAKVSDFGLAKLLGAGKSHITTRVMGTFGYVAPEYANTGLLNEKSDVYSFGVVLLESITGRDPVDYGRPAQEVNLVDWLKMMVGSRRSEEVVDPNIETRPPTRALKRALLTALRCVDPDSDKRPKMSQVVRMLESEEYPIPREDRRQRRSQAGNGESDSQNYDTDKSDNPDPRSESRRSHQV |
| Solyc03g034060 | MGTPQEGGRGWTPPPPPPGANTSSEFSSGYSSHVPGGGPATIPSPNYGGLSKIQFTYADLATATGGFSDANVLGQGGFGFVHKGVLTDGNVVAVKSLKSGSGQGEREFQAEVEIISRVHHRHLVSLVGYCIADGQRMLVYEFVPNGTLEYHLHGKGRPVMDWGLRLKIALGSAKGLAYLHEDCHPRIIHRDIKGANILLDNNYEAMVADFGLARLTEDNNTHVSTRVMGTFGYLAPEYASSGKLSEKSDVFSFGVMLLELITGRRPLDTTNKLMDDSLVDWARPFLTKALEENNYDELVDPRLEGNYDPDELQRMVACAAASIVRALDGDSSLEDLNEKAGKNNTANFGGASGPASDIYDTRAYNADMVKFRQMVMTNQDMNSSEYGNTSDYGLHPSDTSSDFSSDNNHSGPNKQSK |
| Solyc03g121440 | MMSREMKKGKQDMSSDAAEKVMVAVKASKEIPKTALVWSLTHVVQPGDCITLLVVVPSQSSGRKLWGFPRFAGDCASGHWKLHSGNSSEHKSDITDYCSQMILQLHDVYDPNKASIQILFISTAINVKIKIVSGTPHGAVAAEAKKSQANWVVLDKHLKHEKKRCMEELQCNIVVMKRSQPKVLRLNLVGSPKKEPDVTGTLSSEQTQICGKESNKKDSLDSSRGPLVTPSSSPEMFSTTEAGTSSVSSSDPGTSPFFVSEVNRDLKKANLSSAQEDVDESSSESESENLSASSSLRFQPWIADIINSHSELSQIKGKSSLRTHDRPQDSTNKTLLRKFSKLDEESDFGSPSYRADLDYSGNVREAVALSRSAPLGPPPLCSICQHKAPVFGKPPRWFTYAELELATGGFSQANFLAEGGYGSVHRGVLPDGQVVAVKQHKLASSQGDQEFCSEVEVLSCAQHRNVVMLIGFCIEDSRRLLVYEYICNGSLDSHLYGRTRDPLEWSARQKIAVGAARGLRYLHEECRVGCIVHRDMRPNNILITHDFEPLVWKTLSPPLKVGDFGLARWQPDGDTGVETRVIGTFGYLAPEYAQSGQITEKADVYSFGVVLVELVTGRKAVDLTRPKGQQCLTEWARPLLQECAVDELIDPRLENCYSEHEIYCMLHAASLCIRRDPQARPRMSQVLRILEGDLIMESGKLSTTPGYDVGNHSGRIWSDAQQQCQRFSGSSDGSEEFSAKLSFDKRNPSNVWDRSTY |
| Solyc03g116760 | MDFSSIFCCVKGSDRKGQAKKQPTWRVFSLKELHSATNNFNYDNKLGEGGFGSVYWGQLWDGSQIAVKRLKVWSNKAEMEFAVEVEILARVRHKNLLSLRGYCAEGQERLIVYDYMPNLSLLSHLHGQHSAESLLDWKRRMNVAIGSAEGIAYLHHHATPHIIHRDIKASNVLLDSDFQAQVADFGFAKFIPDGATHVTTRVKGTLGYLAPEYAMLGKASESCDVYSFGILLLELASGKKPIEKVNLTVKRTITDWALPLVCEGKLSELADPRLSGNYVEEEFKRVVLVALVCAQNRPEKRPTMLDVIELLKGEAKEKFTALENDDMFKIPPVVDDDVLSGTEGNADSTASEEKEPKPEIEKVEVQA |
| Solyc07g056270 | MSSGDLKSDLSRKTSFLGLKLWVVMGLCVGVFIVGILCMLSIWVTCRRKSRRTLDGYSHCQIPHVSKDIKVDRVGAANVHDHPESLFLTIHDKPNETTSEKMLVHLGRSKSSDADNISQCSSIYHHERGFSSQSGEEGSSGTVRKQPSYGIAMPSPLIGLPEVSQLGWGHWFTLRDLELATNRFSAENVLGEGGYGVVYKGRLINGTEVAVKKLLNNLGQAEKEFRVEVEAIGHVRHKNLVRLLGYCIEGVHRMLVYEYVNNGNLEQWLHGAMRHHGTLTWEARVKVLLGTAKALAYLHEAIEPKVIHRDIKSSNILIDDAFNAKVSDFGLAKLLDSGESHITTRVMGTFGYVAPEYANTGLLNEKSDIYSFGVLVLEAVTGRDPVDYSRSANEVNLVEWLKMMVGNRRAEEVVDSDIEVRPSTRALKRALLVALRCVDPDSEKRPRMSQVVRMLETEEVPYREDRRNRRSRTASMEIESVKESCSSSADVESKVGRADSSTSDTILG |
| Solyc04g006930 | MDSSLLYEGSAPPTDNAPPTSSPPPTPSSPPVSSPPPPSSSPPPSSPPPASSSPPPSSPPPESTSPPPSSPPPKSSSPPPASPPPAPTPPPPVSSPPPTSSPPPALSPPPHVQNPEPPPAPQSHGNSSPPQSPEPKKGSSSSSPPSPPSSNSPGDGGRGGDTPPSPTSKSSENSPPSPALGPSKDSPPSPATLSPSNSPSSDSPSNSSTPTSVIQWSPPPPSSNGAQSSLSPPSSQVPSNHNSGNRSTESPKPAGGNSSGTAESASIGIVIVLLLVGIIGAVVWCIRKRKKKNSVHCSGYVKPISTCSSPKSADPGGFNNAKTWFTYQELVEATNDFSAKNELGKGGFGSVYKGYLADGRYVAVKQLNIGGSQGEREFRAEVEIISRIHHRHLVSLVGYCISENKRLLVYDYVSNNTLYFHLHAQGRPVMNWPTRVKIAVGAARGIAYLHEDCYPRIIHRDIKSSNILLDDNFEAHVADFGLAKLAQDAESHITTRVVGTFGYMAPEYASTGKLTEKSDVYSFGVVLLELITGRKSVDTSQPSGQENLVEWARPLLSRALQKEEFDLLADPCLEKNYVGTEMFRMIEAAASCVRHSSAKRPAMGQIMRAFDGMAIHDLSNGMKVGESAIHSAALQSAEISWFRKMLKDIFMRFDLNGDGSLTQLELAALLRSLGLKPGCGDQLHVLLSKIDHNGNGSVEFDELVDAIMPDMNEDILINQDQLMELFQSFDRDGNGYITAAELAGQMSKMGHPLTYRELSNLMQEADTNGDGVISFNEFANILGKSATDFLGLNTVSESVA |
| Solyc01g010030 | MSIVSPPLSSSTPFAAPPIALNSPAPIYQPNENSSSIASPQSAPLVATSPLPESPPPPTNITLPPPASSPQPQPPISTPTPPAESAPPIISPPPTPSGPPASSLTPPPSSSPPASPTESPSSSTPSSPPPLPPQPEPTRPASSPPPPSTHPPALSPPHSSIRPPALSPPPPVQNPRTPPAPRSPGNPSPEPPKRSPPSSKPPANAPPSKPPRNTPDSPALVPPKNSPPSPDALPPINSASSSSPPTNSSPPQSTNGAQPSLTPPSPSFPSGNTTTSSNSSAVKSKDIGDGGIGIGGSVAIGIILVFLLLGIVGAAGWCICKRKKKDSGLSGGYILPTTLISSPKSGSASLKFRESKPEIGNGAGSNIANTTGNTGGLGQSKPWFTYQELLEATNEFSEHNLLGEGGFGSVYKGCLANGRDIAVKKLNIYGSQGEREFRAEVEIISRIHHRHLVSLVGYCIYENGRLLVYDYVSNDTLYFHLHEQGRPVMDWATRVKIAVGAARGIAYLHEDCCPRIIHRDIKSSNILLDNNFDARVADFGLAKLAQDAKSHVTTRVMGTFGYMAPEYASSGKLTEKSDVFSFGVVLLELITGRKSVDASQPLGEESLVEWARPLLGNALEKEEFDQLTDPRLGTNYIDSEMFQMIEIAAACVRHSAAKRPGMGQIMRAFDSMLMSDLTNGMKVGESAIYNSAEQSAQIRLFRRMAFPSQDVNSDFSSQSTNYSRELSEHV |
| Solyc12g044840 | MSKEVKKGKQEGKSSCDVAEKVMVAVKASNEIPKNALVWALTHVVQPGDYITLLVVVPSQNSGRKIWGFPMFAGDCANGPRKSHSSEQKSDLTYSYSQMILQLQDVYDPTKINLKIKIVSGSPHGAVAAEAKKSQANWVVLDKHLKHEKKCCMQELHCNLVVMRRSQPKVLRLNLVGSPKKEPDVSGSLSSEKAQSCGPELEKNYSLASSRGPLVTPTSSPEIFSVTEAGTSSVSSSDPGTSPFFVTEVNRDLHKAELLALKEDQDVDDSSSESESENLSCSSSRFQPWVVDSVSSHCQLSQHQQISSMRSLDRPQTSTTNSLSEKFTKLDKEGDSGSLSYRDDMDYNRNMRGSGTLTRHAPLGPPPLCSICQHKAPVFGKPPRWFTFTELELATGGFSQANFLAEGGYGSVHRGVLPDGQVVAVKQHKLASSQGDQEFCSEVEVLSCAQHRNVVMLIGFCIEDRRRLLVYEYICNGSLDSHLYGRNREPLEWSARQKIAVGAARGLRYLHEECRVGCIVHRDMRPNNILITHDFEPLVGDFGLARWQPDGDTCVETRVLGTFGYLAPEYTQSGQITEKADVYSFGVVLVELVTGRKAVDLNRPKGQQCLTEWALPLLEECAIVELIDARLGSSYKEHEVYCMVHAASLCIRQDPQTRPRMSQVLRILEGDLMMDSGKMSTTQPRYDVGSQSGRILYERYSGSIRKDELEGLSPKLSFDKRSPSIIRDRDSSHRTAFSDHL |
| Solyc12g098960 | MASDLNFELSKKTHIFHLKVWVLLAIFVGFFIVVILLLLPFCSSRKKSRKSHDTLPISKIPSVCKEIRVDQNSASNYGSHYRNPLAFQDTYCEKDSDKLLAHSNIDKMKDFDNNSQSGSFNYLDKDGTFESGEKEVAGTLNHPSHPANVPSPLSGLPEFSHLGWGHWFTLRDLETATNKFAKDNIIGEGGYGIVYRGQLINGTEVAVKKLLNNLGQAEKEFQVEVEAIGHVRHKNLVRLLGYCIEGTHRLLVYECVNNGNLEQWLHGAMQQHGYLTWEARMKILLGTAKALAYLHEAIEPKVVHRDIKSSNILIDEDFNAKISDFGLAKLLGAGKSHITTRVMGTFGYVAPEYANSGLLNEKSDVYSYGVVLLEAITGRDPVDYGRPAPEVNLVDWLKMMVGSKRSEEVVDPTIETRPSTTALKRALLTTLRCLDPDSDKRPTMSQVVRMLESEEYPIPREGRRRRKNQAGAVETESHNRKSTDSSDRPILMTKTRSDHRTNA |
| Solyc07g062620 | MTCFGLCLGGKASPRHNHVVEIDEEISTIENARVYSYRELRAATEDFCPVNKIGKGGFGSVYKGRLRDGKMAAIKVLSVESKQGVKEFLTEIKVISNIEHENLVKLYGCCAEGDHRILVYNYLENNSLSQTLLGGAHSSLQFSWITRTKICIGVARGLAFLHEEVQPYIVHRDIKASNILLDKDLTPKISDFGLAKLIPADLTHVSTRVAGTLGYLAPEYAMRGQLTRKADVYSFGILLLEIVSGRCNTNKRLPIEEQYLLERAWRLFKKGELIQLVDALLGDDFDVVDACKFLKVSLLCTQVMPKSRPSMSTVVKLLTGEMQVNDEEISEPGMLSDLVSLRNQKNTSSDTLSAGSGKQVDSSSSANTTATHGTMTFTTIDDRKS |
| Solyc12g088750.2 | MSAFLNDEMSKKTSIFGLHLWVVVGICVGAAIVIVLFLISLWYTSKRNSSNPQIKNISAEIKEIRIDPSRTLPENPNSVLVADPLPEPEKETTQNSGGYQRIQIEMGKEHMVKPDRVGSGGGSGHGSGEVRSGEQGGLTAPEVSHLGWGHWYTLRELEVATNFFAHENVIGEGGYGIVYRGVMEDNSYVAVKNLLNNRGQAEREFKVEVEAIGRVRHKNLVRLLGYCAEGAHRMLVYEYVDNGNLEQWLHGDVGPYSPLTWEIRMNIILGTAKGLTYLHEGLEPKVVHRDIKSSNILIDKQWNSKVSDFGLAKLLGSERSYITTRVMGTFGYVAPEYASTGMLNDRSDVYSFGILLMEIISGRNPVDYSRAPGEVNLVDWLKTMVSNRNSEGVLDPKMREKPSSRALKRALLVALRCVDPNAQKRPKMGHVIHMLEVDDFPFRDERRTVRENGRSHHDEMKEKVMDKRIAETGDSSGFETTVETNRSLLPKKEIDDDE |
| Solyc10g051330 | MRNLHLPPPPIIQILNLLCITRIHYPIHYLEIVTTLLLMITTTTIIVLNCRIHLKKDHNPLHLRHHHLHPHHHPLHHPLPALIHLPLMDTSHHHYQKIDRDSSNNNNHKPLFIGIGVSVGLLLLLMLVFLISLCKKKRRRRPHDQMGLYMDNSYRHKRNDYDDNYSVRSSEHVVKIPPTTTSGRVSSEYNWPLAPPPPPPSMSNTSSSNFSSNQQQTFNVASPSNLSSGFINQRHYSYDDLANATGGFSKSNLLGQGGFGYVHKGILPNGKEIAVKSLKSNSGQGEREFQTEVETISRVNHRHLVPLVGYCIAGSQRMLVYDFIPNYTLEYHLHGSGNPVMNFPTRLKIATGTAKGFAYIHEDCHPRIIHRDIKGANILLDNNFEAKVADFGLAKLAADNFTHVSTRIMGTFGYLAPEYASTGKLTEKSDVYSYGVMLLELITGHRPTDVNSDGDNLVDWARPILNRAMEGGNYDELIDPRLEGKFDRQQMLCMVTCAAASIRHSSKRRPKMSQIVRTLEGDVALLNDLNRESTPGIYGSGESSECDGAGSSYGNIKKSKKSEISSEEYTSSEHGSTGEYVQSKAQLQTQNIPLY |
| Solyc11g044460 | MLVFLISCCKRKRRRPRDQMGYYRDNSHGGNSTDYYNSGPYGNWHNNNNIQSTDHMVKMPNPQAPSTNVSSELSWPIAPPPPPPMMSSSEMSSAAFSGPQQPPLPPPHPSMALGFNQSSFTYDDLSTATGGFVKSNLIGQGGFGFVHKGVLPNGKDIAVKSLKANSGQGEREFQAEVEIISRVHHRHLVSLVGYCIAGSQRLLVYEFVPNGTLEYHLHGTGRPVMDFPTRLKIALGSAKGFAYLHEDCHPRIIHRDIKAANILLDHNCEAKVADFGLAKLSNDTNTHVSTRIMGTFGYLAPEYASSGKLTEKSDVYSYGVMLLELITGRRPIDISSDDDTLVEWARPILIRATEGGDYDELIDPRLEGNFDAQEMLCMVACAAASIRHSARRRPKMSQIVRALEGDVSLDALNEGMKKSPSSAMFGSSESSEYDGGSYDLKKFKKSGLSSQEFTSSEHGTGEFVHSDGESQEHGQKRRTP |
| Solyc02g014070 | MERSTNNFDPQLVIGSGGYGTVYKGNIDGGETTVAVKRLKPGSSQGEKELWTEINMLSMHRHENLLSLIGYCIEGHEMLLVYDYMPRGSLADNRYKMDRNSSSLSWERRLKIAIGAACGLDFLHTCQNRDIKSSNILLDENWESKISDFGLSKMGPGNESATHVSTQVKGTTWTKQCIKEGEINKLIDENLLGSISSTCLKAFIGISAKCFYGLPQERSAMFEVVKSLELALVFQKNEGEGIISFDDTSTSSHSRIEAESAATRKFSNATVLGQGSFGKVYKRCLAESPFSKDGRTLIAVHKLNSESSEGFKEWQSEVSILGRLSHPNLVELLGYFQEDKEVLLVYEFMQKGSLNNHLFGSKRLWVCRSAALSLPWNVRVQIVIAAGRGLAFLHASEKQVIYRNFKASIILLDGSYNAKISGFGLAIQGTSDSQPHVSTEIIVRDGYAAPEYVATGHLYVKSDVYPFGVFLVEMLTGLRALDTNRPSNQHYLVDWIKPHLSDKRKLKEKMDSPLGGKYPSRAAVQIA |
| Solyc01g109530 | MSSSSNGDSPSSNSSSSSSPNNSNNNSPPEKDNSSSSNGNDNNNSSNNNNNNNNNNNSNSQSPPSNNDSSSGKDNNSSSSNNNNNNNSNNNNNDNNNNKNNNSNSQSPPSNNNNSSSGKDNNSSSNNNNNGNNNNGNNNNGNNNNDNNNNGNNNDNDNDNNNNNDNKSRSPPNSSKGGSSLAPPFSPPSHFSPNSRPLAPPKPQSDKSDSNDNKTALRIGVAAGAGLLFIVMLVFLISCCKRKKKRKHGQMGYYRDNSHGAMNNHYYNNTGQRTNNWQTNNKLQSTDQFHKMPPPGSGQVSSEHSWPIAPPPPPPMMSSSDMSSSAFSGPHQPAQPPPHPAMALGYNQSSFTYDDLAVATGGFTKDKLLGQGGFGYVHKGVLPNGKEIAVKSLKSNSGQGEREFQAEVEIISRVHHRHLVSLVGYCIAGSQRMLVYEFVANSTLEDHLHGSGRPTMDFNTRHRIALGAAKGFAYLHEDCHPKIIHRDIKAANILLDENFEAKVADFGLAKLSSDNHTHVSTRIMGTFGYLAPEYASSGKLTEKSDVYSYGVMLLELITGRRPIDMDGDDDTLVEWARPILIRATEGGNYDELIDPRLEGNFDAQQMLCMVACAAATIRHSAKRRPKMSQVVRALEDDVTLDDLNEGGKLGHSATLSSGSSEQDGGSYDLKKFRKSSMSSQEYSSSENGESREFRQNKK |
| Solyc07g006610 | MGSSFSCCGDEKVDEGQNNELVGENSWRIFTYKELYAATNGFSENYKLGEGGFGSVYWGKTSDGLQIAVKKLKSMNSKAEMEFAVEVEVLGRVRHKNLLGLRGYCAGNEQRLIVYDYMPNLSLLSHLHGHLSREVQLDWKKRMKIAIGSAEGLLYLHHEVSPHIIHRDIKASNVLLDSNFEPLVADFGFAKLIPEGVSHMTTRVKGTLGYLAPEYAMWGKVSESCDVYSFGVLLLELITGRKPIEKLPNGVKRTITEWMEPMIAKEKFKDLVDPRLKGNFDEIQLKQSIRVAALCVQSEPDKRPTMKEVVCILKGEEVNNVVKANHENLRIQSVRYGDDLLALDQTSDQEDNESSSDKGNESSVYGVFGAMEMKKMQDPYKRFGDK |
| Solyc10g005630 | MTCFPLFHRKHQSSTRHASEFDDELSGVKNVTLFSYKQLRIATDDFSTLNKIGEGGFGSVYKGRLKSGKMAAIKVLSSESKQGVREFLTEIKVISDVDHENLVKLYGCCIEDDHRILVYNYLENNSLAQTLLGGGHSSIQFSWRTRTKICIGVAKGLAYLHEQVKPHIIHRDIKASNILLDKDLTPKISDFGLAKLIPPNATHVSTRVAGTIGYLAPEYAIRGQATRKSDVYSYGVLLIEIVTGRCNTNSRLPIDEQYLLERTWQLYERNELVMLVDTSLDGDFDAEQACRYLKIGLLCTQDALKLRPSMSTILKMLNGEMEVNDNKITKPGLISDFMDLKIKSSAPEQINAAYDYVLSDNTTLPSTSSSQGNSTFTTAAYDQSI |
| Solyc08g068250 | MKQLQYQHHLGLKHNVFMPMAMPLSTPPLIPLPTLALKLKTSLITPISSTRLLCKFNDSDMTSQLEILKPEGKKPDKGVNGIFWILLLNLGIYVADHVFQLVLNMHPTLVSWTRAGKLVEEEEGNFGLWLSYILTGAGANLVSWLILPRNAVSVGASGAVFGLFAISVLVKIDVRRQYTVALDWNLTSVRAGTEYIQVMEAAQASTGLAGGIHGGSALQNVNHIAHLSGALIGVALIWMLSGISSEPDAQNNKKAIPKPPTTVVNFTHSPPPPSPSSPNASLKPIVKPIDDKVALHPPPRRPIFTQPLPKLTSPPPPVTALMFPLNASSAPASRDETSSFKVQSPLSSASIGSVIGISVGVTGAFFIVAIILFVCCRNKLKCGKLRDRESPLPKDDLYRILRQSFRQQNVADGSPVSPKQATPFHPLHCFENNAHSNSLNSEADNHFSPKDHIVDLAFSGGNFTYEELWLATSGFSTSNLLGEGGFGYVHKGVLPTGREIAVKQLKVGSHQGEREFQAEVETISRVHHKHLVSLVGYCMNGTKRLLVYEFVSNRTLEYHLHGEAQSTMEWASRMKIAIGSAKGLAYLHEDCNPTIIHRDIKAANILLDSNFEAKVADFGLAKFLSDSDHHVSHISTRVVGTFGYLAPEYAQSGKTSDKSDIFSFGVMLLELITGRPPIISTESSACSSLAIWAKPFLRSALDGGKLDALVDPCLGQNYNIEEMANMIACAAACVRHSSRKRPRMSQVVRALEGDAYVLDLDEGNRPGQSTICDFDSDGSSYFRKFKKIQRNTLKSKEGW |
| Solyc04g078700 | MSGFLTDELSKRTSIFGLRLWVVLGICVGAAIVLVLFLISIWFTCRRNTSKKTLSVSAKKNPNIPKVSKEIQEIRVDPIRTLPENPKLLPAPAPAPIPEPDSFEEKTQNSDDYQRTLIEIGKGQKIANPGRVGQGGGSSHGSEEARTNEQAIIALPEVSYLGWGHWYTLRELEISTNYFAEENVIGEGGYGIVYRGVMEDNSKVAVKNLLNNRGQAEKEFKVEVEAIGRVRHKNLVRLLGYCAEGAHRMLVYEYVDNGNLEQWLHGDVGPCSPLTWEIRMDIILGTAKGLTYLHEGLEPKVVHRDIKSSNILLDTQWNPKVSDFGLAKLLGAEKSYITTRVMGTFGYVAPEYASTGMLNERSDVYSFGILIMEIISGRNPVDYSRPPGEVTLVDWLKIMVSNRNAEGVVDPKIPEKPSSRALKRVLLVALRCVDPNAQKRPKMGQVIHMLEADDFPFRDERRASREHGGRLYRDGAKERVMDKRVIESGDSSGYESSVQTNWSLVKKQETDDEH |
| Solyc07g027025 | MRKMMLLNKSSPADPPPLCSICQHKAPLFGKPPRWFTYSELEHATSRFSQANFLAEGGYGSVHRGHLPDGQVIAVKQYKSASSQGDLEFCSEVEVLSCAQHRNVVMLIGFCVEDGRRLLVYEYICNGSLDSHLYGRNGHPLNWSARQKIAVGAARGLRYLHEECRVGCIVHRDMRPSNILLTHDFEPLVGDFGLARWQPEGNLGVDTRVIGTFGYLAPEYAQSGQITEKADAYSFGIVLLELVTGRKAIDINRPKGQQSLSEWFILIIRIEQARPLLRKSAISELIDPCLVNCCLEQEVRGMLHCASLCIRRDPNSRPRMSQVLRMLEGDVLVS |
| Solyc03g006890 | MFKRCFNCFGGEQKLEISGQIKNRDYPWDIYTLKELVNATNNFHNDNKIGEGGFGSVYWGRTSKGIEIAVKRLKAMSAKAEMEFAIEVEILGRVRHKNLLGLRGFYAGEDERLIVYDYMPNHSLITHLHGTLSADCLLDWPRRMKIAMGSADGLCYLHHEANPHIIHRDIKASNVLLDSNFQAKVADFGFAKLIPDGVTHLTTRVKGTLGYLAPEYAMWGKVSESCDVYSFGILLLEIISARKPLEKLANGVKRDIVQWALPYIQKGDFNHIADPRLKGKFNLQQLKNTILIAIKCTDGNPENRPSMLQVVDWLNNIIVMEKRKKDIKITAFDALSLLQRTIDL |
| Solyc12g014350 | MFMLFMSIADTSLFINCGGPRTSFEGNNYEADTTNRGPSYFSSPSDRWAFSSSGVYVGLQAASYIASNTFSLDVSGPDFYNTARLAPNSLKYYGLCLQGGSYRVRLHFAEIMFSNDSTYSSLGRRIFDVAIQGRVVLRDFNIMEEANGVGKVITKDFPDVTVSSTLEIHLYWTGKGTNAIPDRGVYGPLISAITVTPNFRVDTGSGLPVGAVIGIVLASIVVLLLVLFALWKKGIFGGKNNQEELELRALDLQTGHFRLRQIKAATNNFDPANKIGEGGFGPVYKGVLADGAIIAVKQLSSKSKQGNREFVNEIGMISALQHPNLVKLYGCCIEGNQLLVIYEYMENNCLARALFGRDDQRLNIDWATRKRICSGIAKGLAYLHEESRLKIVHRDIKCTNVLLDKDLNAKISDFGLAKLDEEENTHISTRIAGTVGYMAPEYAMRGYLTDKADVYSFGVVLLEIVSGKSNTNYRPKEEFVYLLDWAYVLQEQGNLLELVDPRLGTNYSKKEAMRMINVSLLCTNPSPTLRPSMSSVVSMLEGKLPVQAPIIKRTTSDDEMRFKSFEKLSHDSQTTQVSTYSQDSQGQNMNAPWSDSSVSVSVPGKDENVTSTSRLLPDLYNVNLD |
| Solyc10g084110 | MKFSFAFSNCFNPTSEIMEINQIIPYEKDGQRVQKFSIFSYKELKVATHGFGASNRIGEGGFGSVYKGRLEDGSFVAVKVLLVDLESMRGEREFISEIAALSNIRHENLVTLRGYCVDGTKRLLVYDYMENNCLSQTLLGEEQNRSKFTWELRRKISKGIAKGLSYLHEEVNPHVVHRDIKASNIVLDHNFTPKIGDFGLSRLFSKNISHITTRVAGTLGYLSPEYAISGHLTRKSDVYSFGVLLLEIISGCPVIAFDIERGEHFLVNKAWEMYNSGKLLELVDPILNGEFRDDEAVRFLKIGLLCVQEIASLRPKMSSVFEMLNSANYMELDDINIIQPGILADLGDVKIGQKQSSNSFLSNVVTLVKKMLVQSVYFSHTKLA |
| Solyc02g071800 | MGLQCMCCIICGSSISIALTWHGWWCFRNLADNKLNGSVPKSFLNLTSLEALELYENCFTGDLLPFQMNNLQVLYVDANQLSGSIPQQLGNLTQLEELDLRANYFVGELPPSFMKLVNLEYFGAQGNNLSGKFPTFIANWTQLETLDLLGNNFEGSWPKEISSLRSLGYLSLSNVVTRGGAYDFPDLSRMTSLEYLILRKCSLRGPIPGYIWELKELQYLDLSFNELSGQLPNSISTSLTSIFLRENKLNGSLPGWLTKRKNVKPHRYVDVSENLFNITNSEFNAASDDPDVNTFPGCSSNLPYTKDTCDHYCPNNLKYDELYINCGGKEVTVKGHHYYADENPNGSSTFSRDEKGGWGYSSMGLTKFVNKRPESSIIKDTCDLSTTAAVLVETARVAPISLKYYGFCFSSGNYTVKLKFYDIGSSNKQVYPITHTRVFDIDIQRKNVRKNYNIETAEINADGDKIVEYNTSITSHLEIHLYWSGYGSYPESNGPLISAISVIKVVQPPPKHQLSPALKAVIAVSSLSFLALLLLLLRKLGYLGGKRSSKEELKTTELFPGGVYTFRQIKDATQNFNVVNKLGEGGFGPVYKGVLPDGTTIAVKQLSGKSKQGIREFVNEIGTISALQHPNLVKLMGCCAEDNELLLIYEYMENNSLEHALFGPDEEIKSRLNWPTRVKIILGIAKGLTFLHEESKLKIIHRDIKPTNILLDKDLNAKITDFGYAKLNEGEHTHVITRIAGTLGYMAPEYAMRGYLTPKADIYSFGVVTLEIVSGRNSTSCRPSDQTVYLLDSAYVLQEQGNLMDLVDPKLGTDYSWTEAKTILELAMMCTNPSPTLRPTTSEVVKVIEGKTKIKTTSSTVRRSTDEIALTKAMAALSQPSPSESYSTAGPSEATPPISNSNNITNEI |
| Solyc10g074890 | MEGVRYCGYVFTITAIYCCFVIKFTVAQITDPAEVSALVSIKKGLVDNMNYLEDWEKGDPCTSNWTGIHCFNKVGANGYFHVKELRMMAMNLSGSLTPELGQLSQLHFLNFMWNDLTGSIPEEIGNIKSLKLLLLTGNKLSGSLSDKLGYLPNLRIFQIDENQISGRIPKSFSNLNSVQHIHFNNNTLIGQIPPELSNLSTLLHMLMDNNNLSGYLPPEFSILPRLRIIQLDNNNFSRSEIPASYGNMSSLVKLSLRNCTLQGSIPDLSRVPSLHYLDLSWNQLSGSLPDKLSNNMTAIILSHNRLDGSIPKSFSSLPLLQKLTMDNNLLNGSFSTDIWQNKSTSTSSLLIDLRNNSLSDISGTLEPPLNVTLRLQGNPVCRNVNVRNIVKFCGSEAGAEHKKNNSVIVTGACPIAACPKDNYYEYVPNSPVPCTCASPLIVGWRLKSPSFSYFDPYVRHFEQYMTRDLRLDLYQMLIESNYWEEGPRLRMQLKLFPVVGVSTFNKSEVIRISDILQYWEISVVDLFGPYELLSFTLEGPYSYLNPDIQAKHKNKGAIIAIVVSGVFAAFVSAILTVLIKKRHTKYQSILSRKRLSAKLSIKLDGLRSFTFREMTLATNNFNHSNQVGEGGYGAVFKGILADKTIVAIKRAKEGSVQGQKEFLTEISLLSRVHHRNLVSLLGYCDEEGEQMLVYEFMCNGTLRNWLSANCKGALSFATRLEIVLGAAKGILYLHTEAHPPIFHRDIKASNILLDSKMTAKVADFGLSRLAPVQDDEGVLPNHVSTIVKGTPGYLDPEYFLTRKLTDKSDVYSLGVVFLEILTGMRPISHGKNIVREVNLAHGSEKMFSVMDSTMGSYPSECVEKFVALALKCCEDKPEDRPSMLDVVRELETIQSILNMMPDIDADSVDSKAKFNEPKTSSSFSDCTSKDAFLSSSNVSGAYSISGVSLTMPR |
| Solyc01g109590 | MAVPSENHSSGNSLSSFFTSRTPILGLQLYIVIAATVIIMVVVLFLIFLLLRLNQSSKRRRSGGKKSAGLLPLVADVIRDSRTTDLNEIGKVNHLLKKENETIAILRKEDQEVIEIESDGLKGSSGSNESSTSRSDTSSAISGSTESTNIGWGRWYSLKELEMATKGFRAENVIGEGGYGVVFRGVLQDGSVVAVKKLLNNKGQAEKEFRVEVEAIGKVRHKNLAGLLGYCSEGVHRILVYEYIDNGNLEQWLHGDVGSVSPLTWEIRLRIAIGTARGLAYLHEGLEPKVVHRDVKSSNILLDRKWNPKVSDFGLAKLLGPEKSYVTTRVMGTFGYVSPDYASTGMLNEGSDVYSFGVMLMEIITGRSPVDYSRPPGEMNLVDWFKGMVSNRRGEELVDPLIEVHPPPRSLKRVLLVCLRCIDMDANKRPKMGQIVHMLEADEFPFRSEPRLVQEKDPLNPRSAGTNRLQLATKDGAGGDEQKPRGR |
| Solyc02g078530 | MSCCFFGDRKKGHDLVHHDTRDAGGDSIASTKNFSFNELRLATNNFHQINKIGRGGFGTVYKGTLKQGKDVAVKTLAAESRQGLREFLTEIETISNVKHPNLVEIIGCCADGNNRILVYEYLENRSLDRALFGSRTSIKLEWEKRTTICLGTARGLAYLHEELVPHIVHRDIKASNILLDKDYTPKIGDFGLAKLFPDNITHISTKIAGTTGYLAPEYVLGGQLTMKADVYSFGVLILEAVSGKSSSSRIWQGDKRSLLEWSWRLYQEEKLLELVDEELDEFPEREVVRYIKTALFCTQANANRRPMMSQVIEMLSRDIQLNEKELTPPGFFEDSEGSVQSRLKISRGNTSQQSSVHITITQVTPR |
| Solyc12g098980 | MSCFSCCDDDDMHRATDNGPFMAHNSAGNNGGQRATESAQRETQTVNIQPIAVPSIAVDELKDITDNFGSKALIGEGSYGRVYHGVLKSGRAAAIKKLDSSKQPDREFLAQVSMVSRLKDENVVELLGYCVDGGFRVLAYEYAPNGSLHDILHGRKGVKGAQPGPVLSWAQRVKIAVGAAKGLEYLHEKAQPHIIHRDIKSSNILLFDDDVAKIADFDLSNQAPDMAARLHSTRVLGTFGYHAPEYAMTGQLSSKSDVYSFGVVLLELLTGRKPVDHTLPRGQQSLVTWATPRLSEDKVKQCVDARLNTDYPPKAIAKMAAVAALCVQYEADFRPNMSIVVKALQPLLPRPVPS |
| Solyc01g094940 | MSVYDAAFVNSELSKNTSIFGLKLWVVIGIFVGAVFVLIVFLLSLCITASRRRTTTQKGKLHRPINSELTPVVSKEIQEIVHHDTAQDHRPIVLQAVPEIQINMGKVEHRVVFSDKMPGASSGESRATSGAETGSLGNSGQLPEVSHLGWGRWYTLRELEAATNCLSDENVIGEGGYGIVYQGVLTDGTRVAVKNLLNNRGQAEKEFKVEVEAIGRVRHKNLVRLLGYCVEGAYRMLVYEYVDNGTLEQWLHGDVGDVSPLTWDIRMTIIVGTAKALAYLHEGLEPKVVHRDVKSSNVLLDRQWHPKLSDFGLAKLLNAERSYVTTRVMGTFGSVNSAFEINFSLYVAPEYACTGMLNEKSDIYSFGILIMEIITGRTPVDYGRPKGETNLVEWLKMMVGNRQSEEVVDPKLPEMPSSKGLKRALLVALRCVDPDAQKRPKMGHIIHMLEADDLLVRDERRIGRERESSNSHRDYKQDNQAGPKLARKQYGDGAPETSEGNSSRNHNLPSSWR |
| Solyc02g089090 | MATITLLFFLFLQLITATIATTSPPYNATVFVLLNCGAQSAITDDTGRRWDTDTHFPNFLPSDFSSISTTATALEQDPSVNRIPYTTGIRIMRSQFTYTFRVTPGTIFLRLYFYPANYSGFNKADSFFSVTANNLTLLSNFSAFFTVSASSTKAVQKEYVINVDETQMLKLTFSPSPNSYAFVNGIEILSMPTDLYIHGDVKLTGNTIPYNINNSTALETLYRLNVGGNLVESTEDTGMYRVWDSDNAFVVGLGYQTPHFPDANITYTSETPNYTAPTIVYTTSRIMDNYSSGLYWEFPLNSGFLYLFRLHFCEIQPEVKEINDRSFSISIGNQTAQREADVIQWSEGWRIPVYKDYVVRNLDGAQNLTLNLSPNPDSAYQNAILNGLEIFKLNDSNGNLSVPNPEVFFPNNSPPNNNKKKKSSHIIAVITAVAVISGIALFSILCFLIFRRWRRGKDLHTSVTKSSWIPLSITSDSTQRTGGSRSSSLPSDLCRHFLLEEIKTATGNFDEKFVIGYGGFGNVYKGYIDNGATIVAVKRLNPSSKQGVREFETEIHMLSKLRHVHLVSLIGYCDDKNEMILVYDYMANGTLRDHLYKTDNAPLPWKKRLEICIGAAKGLHYLHTGKKDIIIHRDVKSTNILLDDKWVAKVSDFGLSKIGPLSGSGKTHVSTVVKGSFGYLDPEYYKRQQLTEKSDVYSFGVVLFEVLCARPALIPNMPKGQVNLADWACRSCKKGNLQQIIDPNLEGQIAPECLNKFAEAAYNCLKDQGVQRPSMNDVVWNLEFILKLQEAADNRGHKMELNSYPTSPSFPLIMNDHTNISTDEGFEEFSGSNEVGEKYTSSASSMTTTSDDKLKSETIFSEILNPSGR |
| Solyc04g063370 | MASSMGNNSSHTDVDSLITGRIVIALDATRDHPEQEIKRIIEEIRSQGNILHAGDTIIVLGVLHKYLHPMGYQMEAEPLSMFGTHKREIEKEVTKKIDAYVNMLMQSAQDCEGEGVDIEVKITAGTPVRKVVVQEVTTINTTCVVLDRHLRRDLTYYLKHIPCKVALIGDNLFVKVVRPYSIVDADNIEQKLYFSMSKQVPLAPPPTAENTEQSVVSINYSGFVESSEIPENEQVVYNQLEHSTSHEDLSFNSMQERSDRTARGDMKHPIPPVFQKERRLPTSWRSSNTPFLCIACGAKTELYIKDSMRFSFSEIQLATDDFSKDNLLGEGGYGHVYKGRLKDGQVIAAKVRKEESTQGFSEFHSEVYVLSFARHKNIVMLLGYCCKENVNILVYEYICNNSLEWHLFENTENILEWHRRYAIAIGTAKGLRFLHEECRGGPIIHRDLRPSNILLTHDYVPMLGDFGLAKWKTNEDNIYTRILGTLGYLAPEYAENGIVSVRTDVYAFGIVLIQLMSGRKAIDAMREDSHHSLRQWAIPLIERLALHELIDPRIGDSYDTFEVYHMARTAFLCVQNDPELRPSMGEQFAFRSLLLDSVQPKILLLIKRQAVDGLASSSRGVRPSPPDRGAGNWKELYKLCLQEIY |
| Solyc04g050940 | MINEWDSFPTTRCCQNALNFFSHALANQAIQQGNLFLHRDQWGRCTSGSFKHQPSVSISKCGLDHLYQESSRCSTLSLTSVVQNQNFKNVWYNCSRFNSSNFDDACRDCTSAIKSTRDHFLEQLNAKDNGTERAICLVAVVISAATTKLNDPSLIDDFFRCLPGLNTLDKSSEHYIKIRCKYEIFKKEDSLAKALVAIVLATFGMMMVIFLVKYVTRNARAGRKLLRSKPKMFPSCPGLYTFSKAEIENAINYGDEKKFLGRGSAGQVFKGVLPSGQVVAIKQIYRSNTSDSFSRELENLSRVRHPNLVCLFGCCIEDGEQYLVYEYCSAGNLAQHLLRKDMVLSWEQRVKILRDCALALRYLHSYIDGFIVHRDIKLTNILLTEDLDAKLSDFGLAKMLGMEESKVFTDVRGTIGYMDPEYMSNAKLTCASDIYSFGIVALQVLSGQKVIELDLDARDQLTRKAKDVSMGKRGLKDFEDPRLKGEVNSVDFESILQIAVLCVAKSSKGRPTIDVVVEEMEKVLKNTLSENKAAEQSASAALRRSHSVGHVIPV |
| Solyc05g005660 | MNCFKAQVLIMVFSISFDLVFSQTNSTSFNKCVLDFNISSSSSSSRESCTTLDNNWDGFLTHPCCGSPFNRYLRALARWTNQTRLIFLNSTQQMDCLTLMNHNSTDIFSCGIEKLTSGAGGCSDYSEIDVLNELGSRLNSLRDDCRLMDSGGGLTKGCNKCLKTWREITYTSKNDSMKLEDDICRFSMLIFLTSERVADVSWIDKIFHCLGDNSLPLESSADESGNETIRSSKFKTDLSILIGGVVGMVLVVIVALWIYVKRKAEVKPSSERYNESYSEESSYRRLSLKEIYSATDNLSMSNFIGQGIAGKVYKGILAGRQHVAIKHIIKDEQMETFVREVTSLSHIKHPNLVSLLGHYDAPNECFLVYELCHNGNLSEWLFGKSKYLSWKRRLEIALDCARGLLFLHSYPQGSIVHRDIKPANILLSASFEAKLSDFGLSKIISIGHSYASSEVRGTYGYVDPEYQKNRHVNSYGDVYSFGIVLLQLLSGQRVINLDLKNPMPLSKMARNLTKGGNIKEFADPKLEGKFSMKAFELVLKLALTSIGLKQQRPSMEQVVVKLEEALDLSTRVESVDP |
| Solyc02g090110 | MGFHHIIIIRFVFSILLGWLSSSVEATNSTKCNQYCGAAGSYSPRVSYPFGFSEGCGIRLDCTESTGEIRIGEYIIQNVTSETLMVNFSMNCSRPIEDLQQFDRTNFGMTWRNGLLLHNCKVPKSECTILSEILSTRLNIQSCDSKKENVSCYSEARADYLDYKKLKNTGCGTVISSILIGMDNDTMKSSAMFIEFQTMELAWGLEGDCACHNDANCTNVSLPGNRKGFRCRCKDGFVGDGFSDGDGCRKVSRCNPSRYLSGRCGGTTRIGVLVGGIIAGAGLMAALAVLCYCIRRRSASLKKRMSARRLLSEAAGSNSVHVFQYKEIERATNSFSEKQRLGIGAYGTVYAGKLHSDEWVAIKKLRHRDPDGVEQVMNEVKLLSSVSHPNLVRLLGCCIENGEQILVYEFMPNGTLAQHLQRERSSGLPWTIRLTIATETAHAIAHLHSAMNPPIYHRDIKSSNILLDYNFNSKVADFGLSRFGMTDDSHISTAPQGTPGYVDPQYHQNYHLSDKSDVYSFGVVLVEIITAMKVVDFSRSHSEINLAALAIDRIGKGRVDEIIDPFLEPHRDAWTLSSVHRVAELAFRCLAFHRDMRPSMTEVADELEQIRLSSWASLEDNVCMTSSVNSSCSSPRSMSETSFRSTTTKKGVGSRRLIVPQKIANSLAIMEEIKDSSPVSVQDPWLSEESPPSTNRLLGNSGR |
| Solyc02g086590 | MKIEMNISSSNFPLVNWMVILLLNFALICEADPRISESGLICGTNRTTAAIIIPQFVKLMEVVSQRVTDNNWGNHGVNSTNISIYALANCYQDLPHQDCLLCYAASRTRLPRCLPGKSGRIYLDGCFLRYDHYNFFNETTDSAEDRVNCSSSNGVATGQEVATLNASAGNLIDELTKTAVANGGYAAANLNGVYGLAQCWRTLSTSGCKKCLDKASRDIKGCLPSRDARALIAGCYLRYSTQKFLNDPSGNSSGGISKGVIVAIVLGVTAFTMLALSAAYTARKRSLRRKRARINLGKISNSYKKSSLNFKYENLEKATNYFDPSTKVGQGGNGSVYKGTLPNGNVIAVKRLFFNTRQWVDDFFNEVNLIHGIEHKNLVKLLGCSIEGPESLLVYEFVTNKSLDQYLFDKDKVKILRWEERFRIIVGTAQGIDFLHGGSEIRIIHRDIKSSNVLLDENLEAKIADFGLARCFGADKTHLSTGIAGTLGYMAPEYLVKGQLTEKADVYSYGVLVLEIVSGRKSIAFAEDSGSLLQTVWKLYTTNQVTEALDPLLKGDFPREEASKVLKVGLLCTQASVALRPSMSEVVQMLTCEGYQIPEPCQPPFLNSNLLAGGSIKSSIRSLVSNALYKLDESSSYATTTESLSMQSSSTGPHKSDEFLLKESENTK |
| Solyc01g101100 | MAGSLNDELSQKTAVFGLKVWQLIGIFVGIFIVVILLLLTLYLTLRRKSRRGTDNLPVSQIPSVSKEIKEVRVEQVSTNDFSPRDGILLTIHDKTSDKESDKVLVHLGMGKKNGDNSSQSGSFHHVDKEGCGSQSGEEGSSCKSAMYKAYNSHPITAPSPLTGLPEFSHLGWGHWFTLRDLELATGRFSKENILGEGGYGIVYRGNLINGTPVAIKKLLNNLGQAEKEFQVEVEAIGHVRHKNLVRLLGYCIEGTHRMLVYEYVNNGNLEQWLHGAMRHHGYLTWEARMKVLLGTAKALAYLHENIEPKVVHRDIKSSNILIDDDFNAKVSDFGLAKLLGAGKSHITTRVMGTFGYVAPEYANTGLLNEKSDVYSFGVVLLESITGRDPVDYGRPAQEVNLVDWLKMMVGSRRSEEVVDPNIETRPPTRALKRALLTALRCVDPDSDKRPKMSQVVRMLESEEYPIPREDRRQRRSQAGNGESDSQNYDTDKSDNPDPRSESRRSHQV |
| Solyc01g099620  SlRBOHA | MEIENTTDSVRGSRVGFSGSLVSGKKSARFKDDESYVEITLDVRDDSVLVQNIKGADHEAALLASKLEKRPNHTLGSQLSFHLKQVSKELKRMTSSNKFQKIDRSKSGAARALRGLQFMNRNVGTEGWSEVESRFDQLAVDGMLAKTLFGQCIGMKESSEFAEELFDALARKRCITSPAVTKDELHEFWEQITDTSFDARLQTFFDMVDKDADGRITEEEVKEIISLSASANKLSKIEDNSDEYAALIMEELDPGNVGYIELYNLETLLLQAPSHSMNLSTNSRVLSQMLSQKLKPTKERNPFKRCKRRLDYFIEDNWKRIWVMVLWLSICAGLFTWKFIQYKRRAVFDVMGYCVSVAKGGAETTKFNMALVLLPVCRNTITWLRSRTKLGKIIPFDDNINFHKVIAFGVAVGVGLHAISHLTCDFPRLLHATDEEYEPMKPFFGDERPNNYWWFVKGTEGWTGVVMVVLMIIAYVLAQPWFRRNRLNLPSTIKKLTGFNAFWYSHHLFVIVYVLFIIHGYFLYLSKKWYKKTTWMYIAVPMILYACERLLRAFRSGYKAVRILKVAVYPGNVMAVHMSKPQGFKYTSGQYIFVNCSDVSSFQWHPFTISSAPGDDYLSVHIRTLGDWTSQLKTLFSKVCEPPTGDQSGLLRADIGKADYKPRLPKLLIDGPYGAPAQDYKKYDVVLLVGLGIGATPLISIVKDVLNNINQQKDIEDGTKGSKKSPFATKRAYFYWVTREQGSFEWFKGVMDEVSENDQEGLIELHNYCTSVYEEGDARSALITMLQSIHQAKSGVDIVSGTRVKTHFARPNWRQVFKRVTINHPDQKIGVFYCGPQGLVGELRHLSQDFSHKTDTKFEFHKENF |
| Solyc03g117980  SlRBOHB | MQNSENHHPHHHHHHSDTEVIGNDRASYSGPLSGPLNKRGGKKSARFNIPESTDIGTSAGAGAKSNDDAYVEITLDVREDSVAVHSVKTAGGADVEDPELALLAKGLEKKSTLGASLVRNASSRIRQVSQELKRLASLNKRPIPTGRFDRNKSAAAHALKGLKFISKTDGGAGWAAVEKRFDEITASTTGLLPRAKFGECIGMNKESKEFAGELYDALARRRNITTDSINKAQLKEFWDQVADQSFDTRLQTFFDMVDKDADGRITEEEVREIIGLSASANRLSTIQKQSDEYAAMIMEELDPNNLGYIMIENLEMLLLQAPNQTVQRGGESRNLSQMLSQKLKHTQEPNPLVRWYKSFKYFLLDNWQRVWVLLLWIGIMAGLFTWKYIQYKQKAAYGVMGPCVCLAKGAAETIKLNMAIILLPVCRNTITWLRNKTRLGSAVPFDDNLNFHKVIAVAVALGVAIHGLAHLTCDFPRLLNASEEAYEPMIYYFGEQPESYWWFVKGVEGVTGIIMVILMAIAFTLATPWFRRGRVSFPKPFHKLTGFNAFWYSHHLFIIVYTLLIVHGEKLYITKTWYKRTTWMYLTVPLALYAGERLLRAFRSSIKAVKILKVAVYPGNVLALHMSKPQGYKYKSGQYMFVNCAAVSPFEWHPFSITSAPGDDYLSVHIRTLGDWTRQLKTVFSEVCQPPPNGKSGLLRADYLQGENNPNFPRVLIDGPYGAPAQDYKQYEVVLLVGLGIGATPMISIVKDIVNNMKAMDEEENSLENGHGMSNAAQNASPNMAQKRGKSGSASGRNSFNTRRAYFYWVTREQGSFDWFKGIMNEAAEMDHKGVIEMHNYCTSVYEEGDARSALITMLQSLHHAKNGVDIVSGTRVKSHFAKPNWRNVYKRIALNHPEAKVGVFYCGAPALTKELKQHALDFSHKTSTKFDFHKENF |
| Solyc05g025690+ Solyc05g025680^a^  SlRBOHC | MQLMSLFRSSPRSCSNYSRRFDLPEAETSEHVGGAMLPIFFQNNLQNTNNYERDLVEVTLQLDKNSFVLCSVDHKIQIEEENSPPYWLRSPPEKEMIWKAVRNKSSAERALGGLRFISKTSGECDSNNNDIWGKVESRFNALAKNGLLRREDFGECIGMGDSKEFAVGVFDALVRRRRQKMARINKSEFHEFWLQISDQSFHARLEIFFDMADSNGDGKITKEEVQEVRLSLVQLSQSKQIR |
| Solyc06g068680  SlRBOHD | MQNPEDHHSDREIISPSYTTKSNDDKYVEVTLDIRDDTVAVHSVKNATKTKAEEAEIEALGKNLQKKRSFGATIVRNLSKRLRSQPHPPRTIDRSSTAAQNVLKGFKFISRTDGGSGWDTVQQRFDELTANSDSLLPKAKFGECIGMNKESEGFALELFDALARRRNMTSGCISKEQLKEFWEQIANQSFDSRLQTFFDMVDKDADGRLTEEEVREIICLSASANKLSNIQKQAAEYAALIMEELDRERKGYIMLENLKMLLLQAPIQSDGGKGLHRKLSHMLSMKLKPTLETNPIKRWYNNLTYFLLDNWRRVWVLLLWISVMAGLFGYKYVQYRNKAAFDVMGHCVCVAKGAAEVLKLNMALILLPVCRNTITWLRNKTKLGGAVPFDDNINFHKMVAGAIGLAVGIHILAHMTCDFPRLLNASPEKYKPMKPYFGDQPRNYWHFVKGVEGVSGIIMVVLMSIAFTLASQRFRRNKIRLPRPLNKLTGFNAFWYSHHLFVIVYSLLIVHGIELYLTKEWYKKTTWMYLAIPIILYSGERLLRAFRSSVKDVKILKVAMYPGNVLTLQMSKPQGFNYKSGQYMFVNCAAVSPFEWHPFSITSAPGDEYLSVHIRTVGDWTTKLRDVFSEPSQTGRSGLVKAAYMQDNINYYPKVLIDGPYGAPAQDYKEYEVLLLVGLGIGATPMISIVKDIVNNMKEEEYDHDLESTEQKKKSGSGSNFKRVYFYWVTREQGSFDWFKGLMNELAEMDCAEIIEMHNYCTSVYEEGDARSALIAMLQSINHAKNGVDIVSGTRVKTHFARPNWRDVYKRIALNHTDARVGVFYCGAPALTKQLGQLALDFSHKTSTKFDFHKENF |
| Solyc06g075570  SlRBOHE | MVPIDDGDLSRDNSVKWILENAEKDSTSDIHENRNGANGLQELANQKSFKKNFSISRRRNGVVPRMGRMESGATRGLKSLRFLDRSTTGKEGDAWRSVEKRFNQNAVNGRIFREKFGTCIGMGESKEFAGELFDTLARRRKINTENGITIDEVKGFWEDISTQSLDARLHIFFDMCDKNGDGKLSEEEVKEVLVMSASANKLSKFKQHAPTYAALIMEELDPDHLGYIEMWQLEALLRGMVGSEEGEKTLKRSQTLAKTMIPKEYRTPVSKFFYKTSEKIQENWKRIWVLTLWLCINMILFTWKFQQFKRKSAFQIMGYCVCIAKGAGETLKFNMALVLFPVCRRTLTKLRETFLGSIFPFDDNINFHKIIALGIAVATFIHALFHTSCNFVKLTTCPQSKFMTFLGSNFDYHQPSYLDLVASIPGVTGILMTLFMLFSFTLATHSFRRNVIKLPWPFHHLAGFNAFWYAHHLLVLVYILLVLHGYFIYLTKEWYKKTTWMYLAVPVLAYATERTLIVYEHSYNVNIIKAVTYTGNVLALYMSKPPGFKYKSGMYLFVKCPDISTFEWHPFSITSAPDDNYLSVHIRTLGDWTTELKTRFEKACEPDQVAQSRKGSLVRMETKAYSDVEQAQSEFPKIMIKGPYGAPAQNYKKYDILLLIGLGIGATPFISILKDLLNNESQSNGELSSNRRGPDRAYFYWVTREQGSFDWFKGVMDDIAEYDHNEMMEMHNYLTSVYEEGDARSALIAMVQSLQHAKNGVDVVSDSRIRTHFARPNWRKVFSRLAAAHPSSRIGVFYCGSPTLTKPLRRLCQEFSLNSSTRFNFHKENF |
| Solyc07g042460  SlRBOHF | MNEGSERGEEEAPPNGFLARSASAASKLRRKFSWIRSPSVMSRTSAAASEVSDDNYQLHHTSNTLSAREEMKSKLKLVRSKSTAQRALGGLRFISKTTGESDTNVLWKKVEARFDALAKDGLLAREDFGECIGMEDSKEFAVGVFDALIRRRRQKAAKITKIELHDFWLQISDQSFDARLQIFFDMADSNGDGKITRDEVQELIMLSASANKLSKLKERAAEYASLIMEELDPECLGYIELWQLETLLLQRDNYMTYSRPLSTTSVGWGQNLGTLNKTKNLVKRASYAFKCLVLDNWQRGWILLLWVMVMAVLFTWKFLQYRQRAAFQVMGYCLATAKGAAETLKLNMALILLPVCRNILTWLRSTRAKLLLPFDDNINFHKVKCTQTEYIVQHPRIFLTMLFQLQIIAYAIGVGILLHAGNHLACDFPRLINSSPEKFALIASDFDNVKPTYKSLLTGIEGVTGIAMVILMAIVFTLATRTFRRNVLKLPPPFSRLTGFNAFWYSHHLLAVVYVLLLVHGTFLFLVHQWWQKTTWMYISMPLLLYVAERSLRTCRSEHYAAKILKVSVLPGDVFSLTMSKPNSFKYKSGQYIFLQCPTISSFEWHPFSITSAPGDDYLSVHIRMVGDWTNELKRVFTEDDSSACEIGRAKFRERGNVDQRGLPRLLVDGPYGAPAQDYQNYDVLLLVGLGIGATPFISILKDLLNNSRSEELDSTTETSASDDSWTSLASSSMASTGKKKSLRTKSAHFYWVTREPGSFEWFKGVMNEMAEIDHKGLIEMHNYLTSVYEEGDARSTLITMVQALNHAKHGVDILSGTQVRTHFARPNWKEVFNKIASKHPYSTVGVFYCGLPALAKELKKLSQELTYKTSTRFEFHKEYF |
| Solyc08g081690  SlRBOHG | MRGLPGHERRWTSDTVSSGKDLSGESSPGTDSGNISGFASEEFVEVILDLQDDDTIILRSVEPATVINIDGSDPASGVGIGGASIETPASVTSTSETRSPMMRRSTSNKFRQFSQELKAEAVAKAKHFSQELKAELRRFSWSHGHASRAFSPASFFQNAVVGTGNGVDSALAARALRRQRAQLDRTRSSAHKALRGLKFISNNKTNGWNEVENNFAKLAKDGYLYRSDFAQCIGMKDSKEFALELFDALSRRRRLKVDKISKEELYEYWSQITDQSFDSRLQIFFDMVDKNEDGRIGEEEVKEIIMLSASANKLSRLKEQAEEYAALIMEELDPERLGYIELWQLETLLLQKDTYLNYSQALSYTSQALSQNLQGLRKRSPIRRMSTKLVYSLQENWKRIWVLVLWILIMIGLFLWKFYQYKQKSAFQVMGYCLLTAKGAAETLKFNMALILLPVCRNTITFLRSTKLSCFVPFDDNINFHKTVAAAIVTGIILHAGNHLVCDFPKLIHANSTNYQKYLVNDFGPSQPQYIDLVKGVEGVTGIVMVILMAIAFTLATRWFRRSLIKLPKPFDRLTGFNAFWYSHHLLIIVYIVLIIHGTFLYLVHNWYSKTTWMYIAVPVLLYAGERTLRFFRSGLYSVRLLKVAIYPGNVLTLQMSKPPQFRYKSGQYMFVQCPAVSPFEWHPFSITSAPGDDYLSIHIRQLGDWTQELKRVFSEACEQPEAGKSGLLRADENTKTSLPKLLIDGPYGAPAQDYRKYDVLLLVGLGIGATPFISILKDLLKNIVAMEEQADLVSDFSGNSDMSAATSEQPALNKISPKKRKSTLKTTNAYFYWVTREQGSFDWFKGVMNEVAELDQRGVIEMHNYLTSVYEEGDARSALITMVQALNHAKNGVDIVSGTSVRTHFARPNWRKVFSKTLTKHANARIGVFYCGAPILAKELSQLCKEFNQKGTTKFEFHKEHF |
| Solyc11g072800  SlRBOHH | MGETPIENGGGGRTKMVRIESGAARGFKSLHFLDNTTGKEADAWKNVEKRFHINAVNGILFKDKFGPSIGMKSFFFKKNKIGARGKKGFLSTELLNLCDIVFLFFSEMKGMESKEFAEELFDTMARRKKINAENGITIEELKEFWDDISTHCPDTRLHIFFDMCDKNGDGKLSEEEVKEVLVMSASANKLTNFKKHAATYAALIMEEFDPDHLGYIEIWQLESLLRGMVGSEDGQNNMKRSQTLAKTMIPRQYRTPVSKFLSKTSEKIYDNWKRIWVVTLWLSINFILFIWKIEQFKRREAFKIMGYCVCLAKAAGETLKFNMALILIPVCRRTLTQIRESFLGYIIPFDDNINFHKMVAVGITVGAFIHILFHTSCNFVKLASCPESKFMTALGSNFGYQQPTYMDLVVSVPGVTGILMTLFMLFSFTLATHSFRRNIIKLPGSFHHLAGFNAFWYAHHLLIFVYVLLILHGCFIFLTNEWYKKTTWMYLAVPVVLYSTERVLIINENRYHVNIQKAVIYAGNVLALYMTKPPGFKYKSGMYLFVKCPDISNFEWHPFSITSAPDEDYLSVHIRTLGDWTTELRTRFEKACQEAPSKTRNLVRMETKAYNQKDAEQSQAENPMIIIKGPYGAPAQNYKKYDILLLVGLGIGATPFISIIKDILNQKSGYNQQDDGKKGPQRAYFYWVTREQGSFDWFKGVMDDIAEHDENEVIEMHNYLTSVYEEGDARSALITMVQSLQHAKNGVDVVSESRIRTHFSRPNWKRVFAQLAVTHPSSRIGVFYCGSPTLTKPLKKLCQEFSLNSSTRFNFHKENF |
| AT5G47910  AtRBOHD | MKMRRGNSSNDHELGILRGANSDTNSDTESIASDRGAFSGPLGRPKRASKKNARFADDLPKRSNSVAGGRGDDDEYVEITLDIRDDSVAVHSVQQAAGGGGHLEDPELALLTKKTLESSLNNTTSLSFFRSTSSRIKNASRELRRVFSRRPSPAVRRFDRTSSAAIHALKGLKFIATKTAAWPAVDQRFDKLSADSNGLLLSAKFWECLGMNKESKDFADQLFRALARRNNVSGDAITKEQLRIFWEQISDESFDAKLQVFFDMVDKDEDGRVTEEEVAEIISLSASANKLSNIQKQAKEYAALIMEELDPDNAGFIMIENLEMLLLQAPNQSVRMGDSRILSQMLSQKLRPAKESNPLVRWSEKIKYFILDNWQRLWIMMLWLGICGGLFTYKFIQYKNKAAYGVMGYCVCVAKGGAETLKFNMALILLPVCRNTITWLRNKTKLGTVVPFDDSLNFHKVIASGIVVGVLLHAGAHLTCDFPRLIAADEDTYEPMEKYFGDQPTSYWWFVKGVEGWTGIVMVVLMAIAFTLATPWFRRNKLNLPNFLKKLTGFNAFWYTHHLFIIVYALLIVHGIKLYLTKIWYQKTTWMYLAVPILLYASERLLRAFRSSIKPVKMIKVAVYPGNVLSLHMTKPQGFKYKSGQFMLVNCRAVSPFEWHPFSITSAPGDDYLSVHIRTLGDWTRKLRTVFSEVCKPPTAGKSGLLRADGGDGNLPFPKVLIDGPYGAPAQDYKKYDVVLLVGLGIGATPMISILKDIINNMKGPDRDSDIENNNSNNNSKGFKTRKAYFYWVTREQGSFEWFKGIMDEISELDEEGIIELHNYCTSVYEEGDARVALIAMLQSLQHAKNGVDVVSGTRVKSHFAKPNWRQVYKKIAVQHPGKRIGVFYCGMPGMIKELKNLALDFSRKTTTKFDFHKENF |
